# Centromere and kinetochore gene misexpression predicts cancer patient survival and response to radiotherapy and chemotherapy

**DOI:** 10.1038/ncomms12619

**Published:** 2016-08-31

**Authors:** Weiguo Zhang, Jian-Hua Mao, Wei Zhu, Anshu K. Jain, Ke Liu, James B. Brown, Gary H. Karpen

**Affiliations:** 1 Biological Systems and Engineering Division, Lawrence Berkeley National Laboratory, One Cyclotron Road, Mailstop 977, Berkeley, California 94720, USA; 2 Department of Molecular and Cell Biology, University of California, Berkeley, California 94720, USA; 3 Department of Translational Bioinformatics, Cellular Biomedicine Group, Inc., Level 5, Building 1, 333 Guiping Road, Shanghai 200233, the People's Republic of China; 4 Department of Therapeutic Radiology, Yale School of Medicine, Yale University, New Haven, Connecticut 06510, USA; 5 Ashland Bellefonte Cancer Center, 122 St Christopher Drive, Ashland, Kentucky 41101, USA; 6 Environmental Genomics and Systems Biology Division, Lawrence Berkeley National Laboratory, One Cyclotron Road, Mailstop 977, Berkeley, California 94720, USA; 7 Department of Statistics, University of California, Berkeley, California 94720, USA; 8 Department of Environmental Bioinformatics, University of Birmingham, Birmingham, B15 2TT, UK

## Abstract

Chromosomal instability (CIN) is a hallmark of cancer that contributes to tumour heterogeneity and other malignant properties. Aberrant centromere and kinetochore function causes CIN through chromosome missegregation, leading to aneuploidy, rearrangements and micronucleus formation. Here we develop a Centromere and kinetochore gene Expression Score (CES) signature that quantifies the centromere and kinetochore gene misexpression in cancers. High CES values correlate with increased levels of genomic instability and several specific adverse tumour properties, and prognosticate poor patient survival for breast and lung cancers, especially early-stage tumours. They also signify high levels of genomic instability that sensitize cancer cells to additional genotoxicity. Thus, the CES signature forecasts patient response to adjuvant chemotherapy or radiotherapy. Our results demonstrate the prognostic and predictive power of the CES, suggest a role for centromere misregulation in cancer progression, and support the idea that tumours with extremely high CIN are less tolerant to specific genotoxic therapies.

## Abstract (web summary)

Centromeres and kinetochores are important in maintaining chromosomal stability. Here, the authors show that overexpression of a subset of centromere and kinetochore genes is associated with chromosomal instability and mutation burden in cancer, and predict patient survival and response to genotoxic therapies.

## Graphical abstract

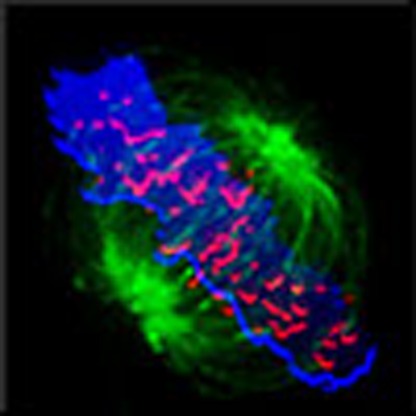

Genomic instability is characteristic of most human cancers and is believed to promote other cancer hallmarks^1^. The major type of genomic instability is chromosomal instability (CIN), which is observed in both pre-cancerous lesions and malignant growths^2^. CIN is characterized by an increased frequency of chromosome abnormalities, including gain/loss of whole chromosomes or large segments (aneuploidy), structural rearrangements and focal aberrations (for example, amplifications and deletions)^3^,^4^. These changes can interfere with normal genome structure and function, increase mutation frequencies and epigenetically modify gene activity^5^,^6^,^7^.

CIN can allow the rapid accumulation of changes that promote cancer progression, growth and heterogeneity, and contribute to intrinsic and acquired drug resistance^8^,^9^,^10^. For example, chromosomal translocations can generate oncogenes that encode fused or misregulated signalling molecules^11^. Moreover, amplification of the epidermal growth factor receptor locus contributes to an acquired resistance to epidermal growth factor receptor inhibitors in glioblastoma cells^9^. Paradoxically, extreme CIN can also hinder cell growth or sensitize cancer cells to therapeutic agents, presumably due to excess genotoxicity and proteotoxicity^5^,^12^. These opposing effects, and the possibility of selectively killing cancer cells displaying CIN, suggest that CIN is both a challenge to and a potential opportunity for cancer treatment^13^,^14^.

The exact causes of CIN in most sporadic cancers remain unclear. Proposed mechanisms include oncogene-induced replication stress, breakage–fusion–bridge cycles induced by telomere dysfunction or translocations, and aberrant mitosis^6^,^15^,^16^,^17^. Another possible mechanism involves centromeres and their associated kinetochores. These structures are required for proper spindle attachment, chromosome congression, mitotic checkpoint activity and separation of sister chromatids during mitosis^18^,^19^. Consequently, their misregulation results in chromosome abnormalities and DNA damage through various pathways, and thus may be an important potential cause of CIN in human cancers^20^,^21^.

Centromeres and kinetochores consist of centromeric chromatin, as well as inner and outer kinetochore structures (Fig. 1a). A key epigenetic mark that determines centromere identity is CENP-A, a histone H3 variant enriched only at active centromeres^22^,^23^,^24^,^25^. CENP-A chromatin and the outer kinetochore are connected by the Constitutive Centromere Associated Network (CCAN) that contains several subcomplexes^26^. These include the CENP-T/-W/-S/-X complex, which resides within the H3 domains interspersed between blocks of CENP-A nucleosomes^27^,^28^,^29^. CENP-C and CENP-N/-L/-M regulate the localization of CENP-H/-I/-K, which in turn is required for CENP-O/-P/-Q/-R/-U recruitment. The CCAN recruits the KMN network (KNL1 complex, MIS12 complex and NDC80 complex) to the outer kinetochore, where NDC80 and other components interact with spindle microtubules to ensure proper chromosome segregation^30^,^31^. All these centromere and kinetochore proteins ultimately require CENP-A for their localization^22^.

Maintaining centromere identity requires CENP-A nucleosome assembly at centromeres in each cell cycle. CENP-A assembly relies on the HJURP chaperone and assembly factor^32^,^33^ that is recruited to the centromere by the MIS18 complex, composed of MIS18A, MIS18B and M18BP1 subunits^34^,^35^,^36^. This assembly also requires several CCAN components such as CENP-C, and the CENP-H/-I/-K and CENP-N/-L/-M complexes^35^,^37^,^38^. Defects in CENP-A deposition cause centromere propagation failures, ultimately producing chromosome segregation errors and aneuploidy^32^,^33^.

The levels of centromere and kinetochore proteins are tightly regulated, and both depletion and overexpression of these proteins can result in chromosome abnormalities and cell death^22^. Reduced levels cause missegregation and chromosome gains and losses^25^. Conversely, overexpression or ectopic tethering of CENP-A or HJURP results in their mislocalization to non-centromeric chromatin, generating neo-centromeres, dicentric behaviour and chromosome bridges that drive aneuploidy, genome rearrangements and micronucleus formation^34^,^39^,^40^,^41^. Interestingly, co-overexpression of CENP-A and HJURP produces more severe chromosome missegregation and micronuclei phenotypes than single overexpression^41^, suggesting synergistic effects among individual centromere and kinetochore protein genes (hereafter CEN/KT genes). Importantly, individual overexpression of several centromeric proteins, including CENP-A, HJURP and others correlates with poor prognosis for several cancers, suggesting roles for these proteins in cancer aetiology^42^,^43^.

Here we test the hypothesis that misregulation of CEN/KT genes causes chromosomal abnormalities that contribute to tumorigenesis, and can be used as a biomarker for predicting patient prognosis and response to therapy. We show that overexpression of 14 CEN/KT genes is observed consistently in a wide spectrum of cancer types, and correlates with the level of genomic instability in diverse tumours and with adverse tumour properties in a cancer-type-specific manner. The Centromere and kinetochore gene Expression Score (CES) signature based on the expression levels of the 14 CEN/KT genes not only prognosticates cancer patient survival independently from established clinicopathological factors, especially for patients with early-stage lesions, but also predicts patient outcome after adjuvant chemotherapy or radiotherapy. Importantly, these results suggest that although a high CES value is correlated with reduced cancer cell tolerance to specific genotoxic therapies, it does not confer a growth or survival disadvantage for untreated tumours. We conclude that the CES signature is an effective prognostic and predictive biomarker, and could be used in clinical applications to choose effective therapeutic regimens. In addition, these results demonstrate the importance of applying knowledge of basic biological functions to cancer research, and suggest that further investigations into centromere misregulation will reveal new mechanisms involved in cancer progression.

## Results

### A subset of CEN/KT genes is misregulated in human cancers

**Figure 1:**
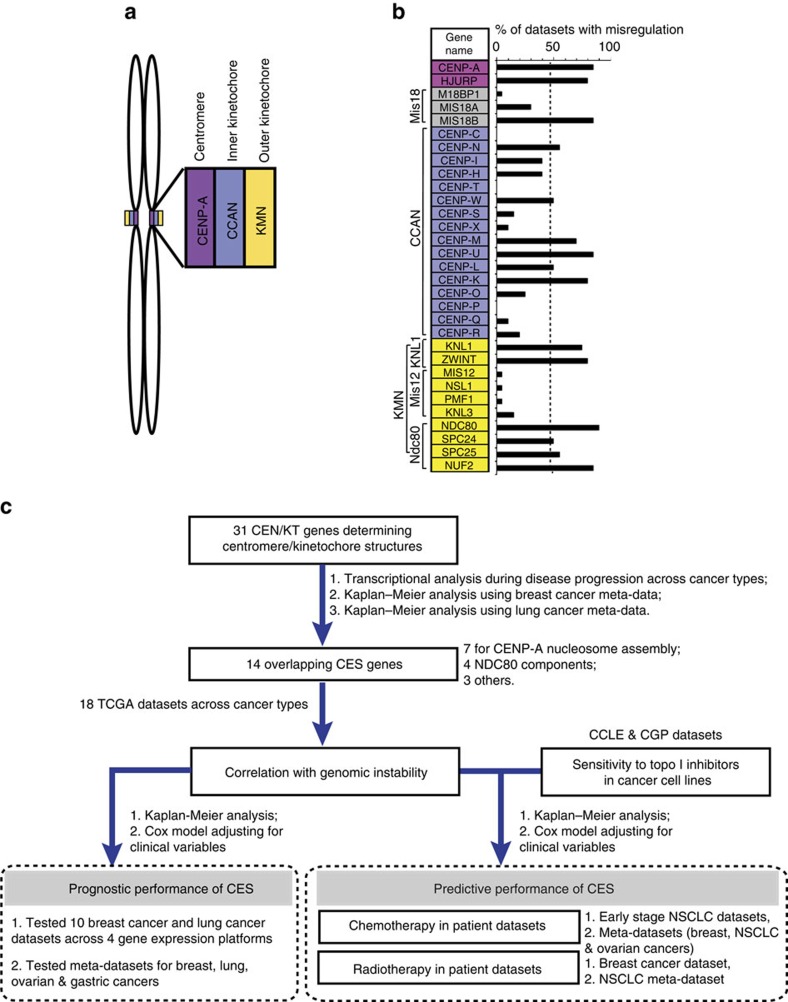
Summary of the approach and transcriptional misregulation of CEN/KT genes across cancer types. (**a**) Schematic overview of the centromere and kinetochore on replicated mitotic sister chromatids. CENP-A nucleosomes (purple) are the structural base for centromeric chromatin and kinetochore formation, and the CCAN network (blue) in the inner-kinetochore connects CENP-A chromatin to the KMN network (yellow) at the outer kinetochore. (**b**) The list of 31 CEN/KT genes. Cells are highlighted with colours matching (A) except for *HJURP* (purple) and Mis18 complex members (gray), which transiently localize to centromeres for new CENP-A assembly. The Affymetrix probes for *CENP-P* did not pass the specificity qualifier filter, and *CENP-P* was indicated with no value and subsequently removed from all other analysis. The graph to the right shows that 15 out of 31 CEN/KT genes are misexpressed (fold change ⩾2-fold, FDR-adjusted *P*<0.05) in >50% of data sets for nine cancer types, specifically breast, cervical, head and neck (including nasopharyngeal), colon, gastric, brain and CNS, liver, lung, and pancreatic cancers, compared with their corresponding normal tissues, or between late- and early-stage lesions. Also see Supplementary Data 1. (**c**) A flow chart showing the overall research strategy.

Motivated by the potential relevance of centromere and kinetochore function to genome stability and cancer aetiology, we manually compiled a list of 31 centromere and kinetochore (CEN/KT) protein genes (Fig. 1b) to investigate their potential roles in cancer prognosis using the research strategy illustrated in Fig. 1c. This list was restricted to proteins known to have an impact on centromere or kinetochore structure and function^23^, including CENP-A, downstream CCAN and KMN components, and factors required for CENP-A nucleosome assembly and centromere propagation (for example, HJURP and MIS18)^32^,^33^,^36^.

Using Gene Expression Omnibus (GEO) databases, we analysed CEN/KT gene expression levels in 13 data sets from 12 different types of human cancers, including breast, lung, liver and prostate (Supplementary Table 1). Each data set contains both normal and tumour samples. Compared with the corresponding normal tissues, diverse cancer types displayed misregulation of many CEN/KT genes (Supplementary Fig. 1). Breast, prostate and liver cancers displayed progressively increasing CEN/KT expression during disease progression (Supplementary Fig. 1B,H,I). Notably, misregulation was not present in mitotically active liver dysplasia and many breast ductal carcinoma *in situ* (DCIS) (Supplementary Fig. 1B,I), suggesting that defective CEN/KT gene expression is not simply a result of over-proliferation. Furthermore, we performed expression correlation network analysis using large TCGA data sets and found that CEN/KT gene expression levels across cancer types, and among individuals within the same type, can differ significantly (Supplementary Fig. 2; Supplementary Note 1).

To understand the potential role of CEN/KT genes in cancer progression, we analysed 13 Affymetrix gene expression microarray data sets from nine cancer types (Supplementary Data 1). Comparing the expression levels in tumours with those in corresponding normal tissues, and in late- versus early-stage tumours, revealed that 15 out of 31 CEN/KT genes were significantly misregulated (all upregulated; false discovery rate (FDR)-adjusted *P*<0.05, at least twofold difference, and in at least 50% (as empirical prevalence cutoff) data sets examined; Fig. 1b; Supplementary Data 1). Their significant overexpression in individual data sets was further confirmed by a permutation test, where we computed the probability of getting CEN/KT gene overexpression that is higher than the observed fold changes after data randomization (Supplementary Table 2). These results indicate that defective CEN/KT gene regulation is present among a wide array of cancers, and thus may play an important role in aetiology and disease progression.

### A subset of CEN/KT genes are prognostic for patient survival

**Figure 2:**
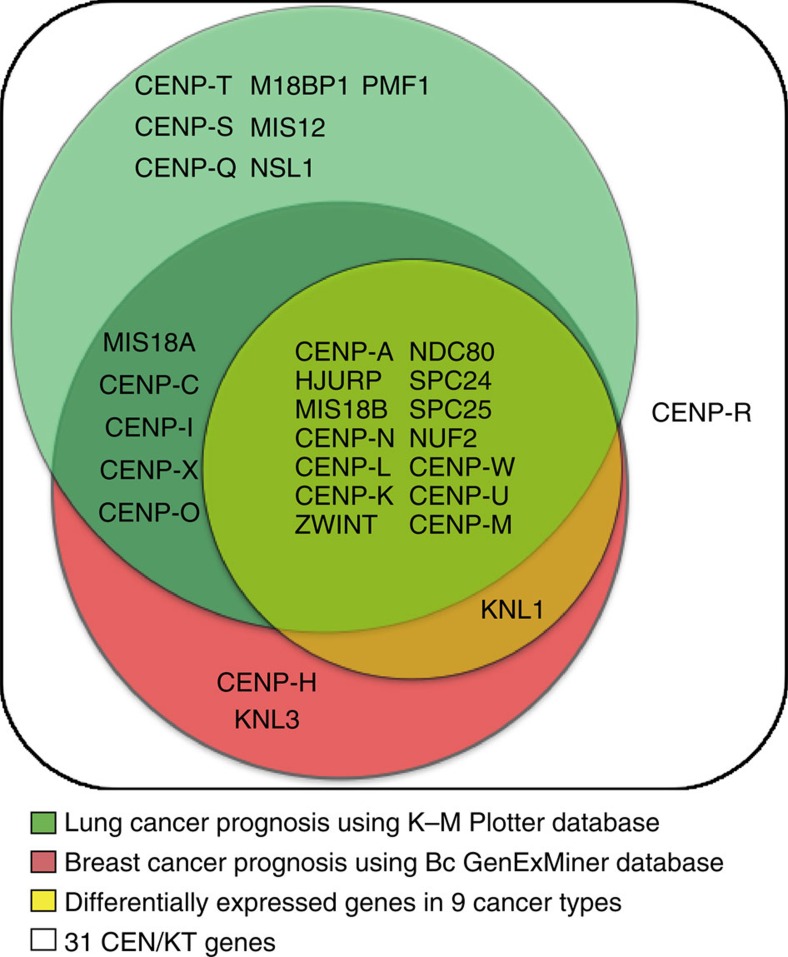
Venn diagram identifies 14 CEN/KT genes whose expression levels correlate with cancer progression. White: All 31 CEN/KT genes. Yellow: CEN/KT genes that are differentially expressed in nine cancer types relative to their corresponding normal tissues as shown in Fig. 1b. Red: CEN/KT genes with significant prognostic values for breast cancer patients using BC-GenExMiner, and (green) those with significant prognostic values for lung cancer patients using K–M Plotter based on Affymetrix HG-U133A platform. Note that inclusion of five genes (*CENP-W*, *CENP-K*, *CENP-L*, *NUF2* and *SPC24*) in the final core CEN/KT gene list was based on the upregulated gene list and BC-GenExMiner results (also see Methods).

Next, we identified a subset of CEN/KT genes whose misregulation offers prognostic value for cancer patients by performing meta-analysis for multiple cancer types using multiple databases. The analyses included over 3,000 human breast cancer clinical samples (using Breast Cancer Gene-Expression Miner v3.0 (BC-GenExMiner 3.0))^44^, and hundreds to thousands of breast, lung, ovarian and gastric cancer patients (using the Kaplan–Meier (K–M) Plotter database)^45^ (Supplementary Tables 3–7; Supplementary Note 2). Combined with the list of 15 CEN/KT genes upregulated in multiple cancers, these results identified 14 out of 15 CEN/KT genes whose expression levels were significantly associated with poor patient survival and higher risk of disease progression (Fig. 2; Supplementary Table 8). *CENP-W*, *-L*, *-K*, *SPC24* and *NUF2* were included in the final core CEN/KT gene list because they were identified by both differentially expressed gene analysis across cancer types and BC-GenExMiner analysis, even though the Affymetrix HG-U133A platform used by K–M Plotter is lack of probes to evaluate these five genes (also see Methods). Altogether, the list of 14 core genes contains seven genes involved in CENP-A assembly (*CENP-A*, *-N*, *-M*, *-K*, *-L*, *HJURP* and *MIS18B*), implying an important role for this biological process in cancer progression. It also includes all four NDC80 subunits and several other CCAN and KMN network components, but no MIS12 complex members. These 14 genes are infrequently mutated in cancer patients, as would be expected for essential genes. The mutation frequencies for all 14 genes combined range from 0 to <14% for different cancer types (Supplementary Table 9). We find no evidence for recurrent cancer mutations using the COSMIC database (http://cancer.sanger.ac.uk/cosmic), and none of these 14 CEN/KT genes have been identified as putative cancer mutation driver genes in recent comprehensive analyses using TCGA and other data sets^46^,^47^. We conclude that expression levels of many but not all CEN/KT genes are effective prognostic factors for multiple cancers, and different CEN/KT genes may have distinct roles in cancer aetiology.

### CES correlates with genomic instability in human cancers

To facilitate further analysis of the impact of CEN/KT misexpression on cancer progression and outcomes, we summarized the extent of overall pathway misregulation in samples using the CES, calculated as the sum of the log_2_(mRNA expression level) for each of the 14 CEN/KT genes (Fig. 2). Since defects in centromere and kinetochore function lead to CIN in experimental systems^22^, we analysed TCGA data sets^48^ for 18 different cancer types to determine if CEN/KT gene misregulation (represented by the CES value) correlates with the extent of genome instability (genome fraction with copy-number alterations (CNA) and mutation frequency). This expansive data set includes high-quality gene expression and genomic data for many patients. We detected a significant positive correlation (Spearman correlation, FDR-adjusted *P*<0.05) between CES values and both CNA fractions and mutation frequencies for seven cancer types (39%), including breast, lung and stomach adenocarcinomas (ADC) and low-grade brain gliomas (Table 1). We found that CES values significantly correlate with either CNA fraction or mutation frequency for six cancers (33%), including adrenocortical, head and neck, and kidney renal clear cell carcinomas. Five of the analysed cancer data sets showed no correlation (28%), including cervical squamous cell carcinomas (SCCs), glioblastomas and thyroid carcinomas. Moreover, within breast ADCs, CES values correlate with the level of genomic instability for both invasive ductal carcinoma and invasive lobular carcinoma (Supplementary Table 10). Overall, we conclude that across diverse cancer types, the CES signature significantly correlates with the level of genome instability, consistent with the important role of centromeres in genome maintenance across tissues and cell types.

### CES correlates with specific adverse tumour features

We next explored the clinical information in TCGA and other microarray data sets and found that CES is significantly associated with several unfavourable tumour characteristics in a cancer-type-specific manner (Table 2). We also confirmed the associations in single data sets by box plots. For example, in breast cancer, high CES tumours are enriched for high-grade (*P*<0.0001, Kruskal–Wallis test; Supplementary Fig. 3; Table 2), ER^−^ and PR^−^ status (*P*<0.0001, Wilcoxon rank-sum test; Supplementary Fig. 4; Table 2), and more aggressive molecular subtypes (basal like, HER2^+^ versus luminal; *P*<0.0001, Kruskal–Wallis test; Supplementary Fig. 5; Table 2). Moreover, breast invasive lobular carcinomas are predominantly luminal A subtype tumours and show significantly lower CES than invasive ductal carcinomas as expected (Supplementary Fig. 6). More details on breast cancer analysis are provided in Supplementary Note 3. In non-small cell lung cancer (NSCLC), high CES tumours are enriched for SCC versus ADC, even though NSCLC histological subtype lacks consistent prognostic value between ADC and SCC^49^ (*P*<0.0001, Wilcoxon rank-sum test; Supplementary Fig. 7; Table 2).

Although we did not detect significant enrichment for advanced stage (III and IV) in high CES tumours for breast or lung cancer data sets (Table 2; Supplementary Fig. 8), we did find that stage II tumours have higher CES values than stage I tumours in early-stage lung ADCs (*P*<0.01, Wilcoxon rank-sum test; Supplementary Fig. 8). The apparent trend of association between stage II and high CES in TCGA breast cancer data set is primarily due to enrichment of basal-like and HER2^+^ subtypes in stage II compared with stage I tumours (Supplementary Fig. 8; Supplementary Table 11). In agreement with this notion, the difference in CES between stages was eliminated when patients were stratified by PAM50 subtype, but the difference among subtypes was not eliminated when patients were stratified by stage (Supplementary Fig. 9).

We also observed a significant association between high CES and lymph node invasion by box plots in TCGA lung SCCs (*P*=0.003, Wilcoxon rank-sum test) but not in breast cancer data set (Table 2; Supplementary Fig. 10). Breast cancer data set GSE3494 was excluded from the analysis due to significant enrichment of high grade in samples with positive lymph nodes (*P*<0.001, Fisher's exact test). Overall, these findings suggest that the genomic instability associated with high CES may contribute to a number of important tumour properties.

### CES prognosticates cancer patient survival and recurrence

**Figure 3:**
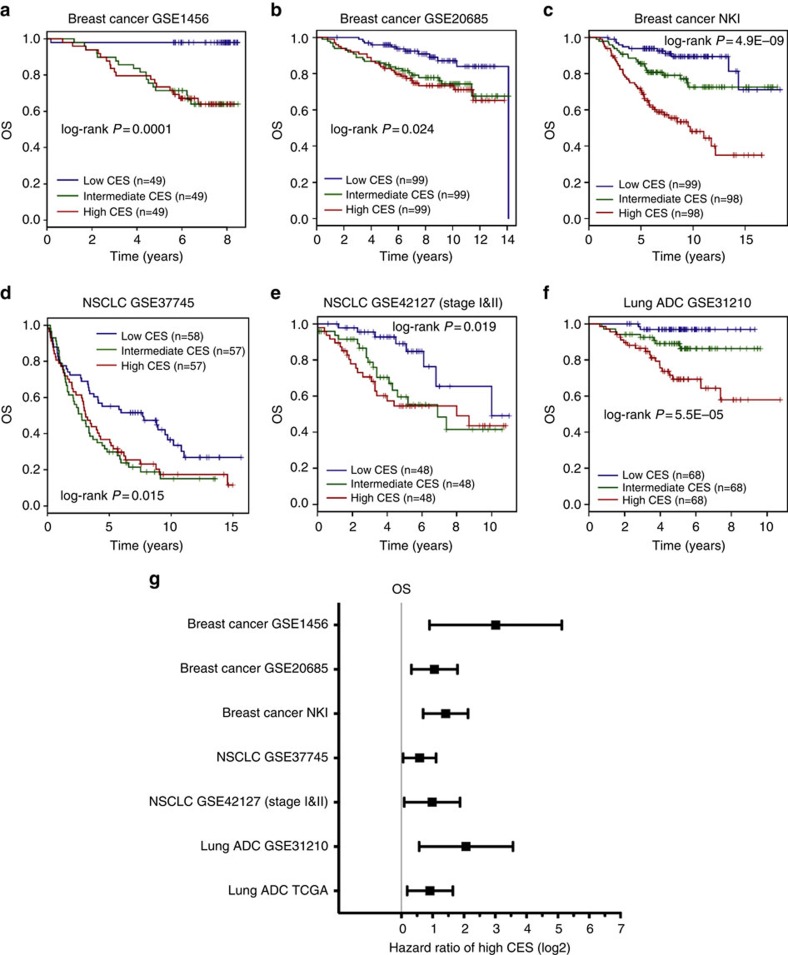
CES signature is prognostic for cancer patient overall survival. (**a**–**f**) Kaplan–Meier survival estimations for individual breast cancer and lung cancer data sets for overall survival (OS). Each cohort was divided into CES high (red), intermediate (blue) and low (green) tertiles. Sample sizes and log-rank *P* values are indicated. (**g**) Forest plot summarizing prognostic impact of CES on patient OS by multivariate Cox regression using individual data sets. Squares and error bars in the plots denote log2 scales of hazard ratio (HR) and 95% confidence interval (CI), respectively. See details for multivariate Cox regression analysis for each data set in Supplementary Tables 15–25. Breast cancer NKI data set, lung ADC GSE31210 data set and NSCLC UT SPORE GSE42127 subcohort contain only stage I and II samples.

**Figure 4:**
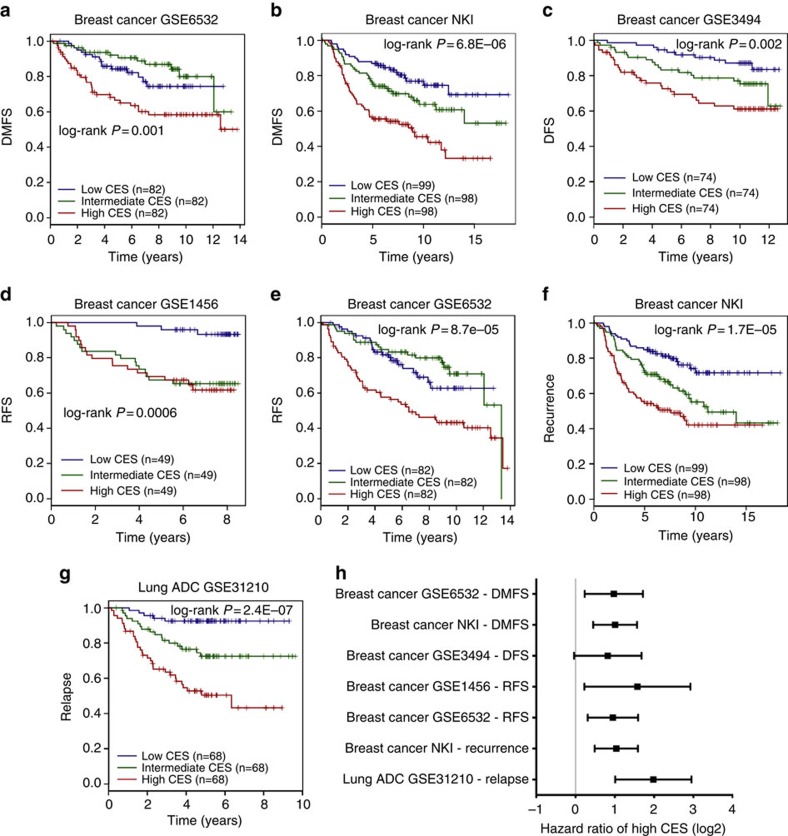
CES signature is prognostic for cancer patient risk of relapse and metastasis. (**a**–**g**) Kaplan–Meier survival estimations for individual breast cancer and lung cancer data sets for distant metastasis-free survival (DMFS), relapse-free survival (RFS)/disease-free survival (DFS) and relapse/recurrence. Each cohort was divided into CES high (red), intermediate (blue) and low (green) tertiles. Sample sizes and log-rank *P* values are indicated. (**h**) Forest plot summarizing prognostic impact of CES on DMFS, RFS/DFS and relapse/recurrence by multivariate Cox regression using individual data sets. Squares and error bars in the plots denote log2 scales of hazard ratio (HR) and 95% confidence interval (CI), respectively. See details of multivariate Cox regression analysis for each data set in Supplementary Tables 15–25. Breast cancer NKI and lung ADC GSE31210 data sets contain only stage I and II samples.

We evaluated the utility of the CES signature for prognosis of patient survival using large, well-defined breast and lung cancer gene expression data sets across several microarray platforms (Supplementary Tables 12–13). These data sets contains relevant clinicopathological information as well as patient survival data. Patients for each data set were stratified into high, medium and low CES groups by dividing the full CES range into tertiles. Then, Kaplan–Meier survival estimation demonstrated that the CES signature effectively predicts overall survival, distant metastasis-free survival and relapse-free survival (Figs 3a–f and 4a–g). In each data set, patients with higher CES values had significantly worse prognoses. We confirmed the significance of the CES signature by meta-analysis across cancer types using K–M Plotter database, using automatically computed best CES thresholds to detect the most significant difference between high and low CES groups (Supplementary Fig. 11). Stratifying patient cohorts according to CES tertiles in K–M Plotter produced similar results. Notably, the CES signature effectively prognosticates patient survival for early-stage (stage I and II combined) NSCLC and ovarian cancer (Figs 3 and 4; Supplementary Fig. 11).

**Figure 5:**
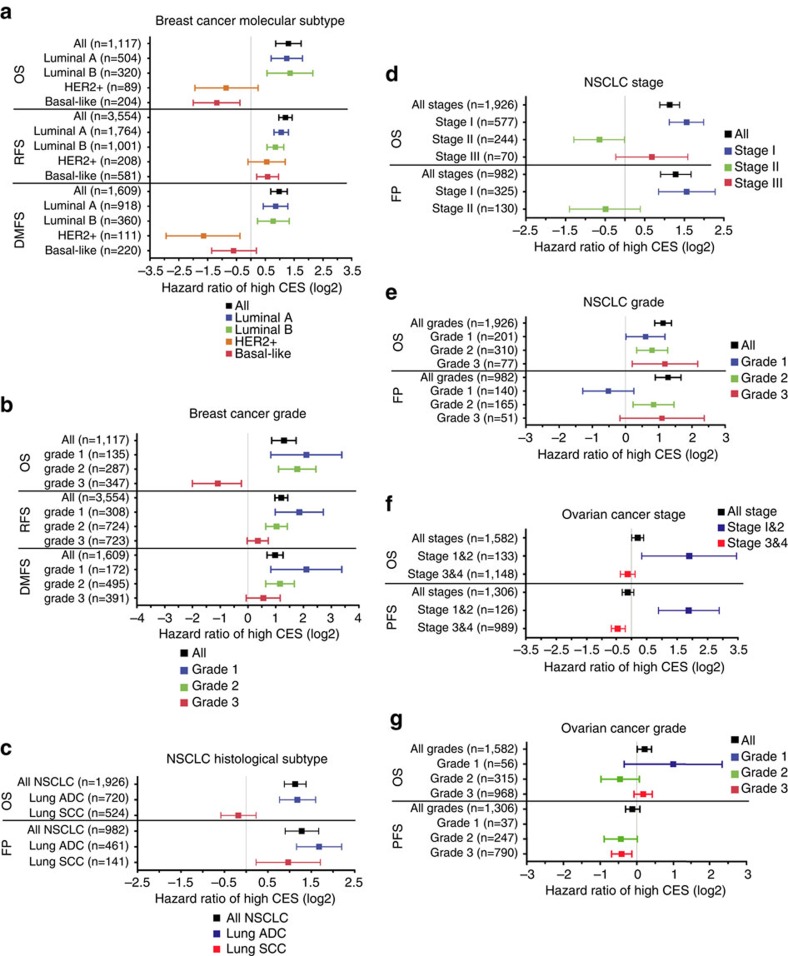
CES signature is prognostic after patient stratification by common clinicopathological factors. CES threshold for high and low were automatically computed using K–M Plotter database. Squares and error bars in the plots denote log2 scales of hazard ratio (HR) and 95% confidence interval (CI), respectively. (**a**) Breast cancer molecular subtypes (see Supplementary Fig. 12 for individual Kaplan–Meier plots). (**b**) Breast cancer grades (Supplementary Fig. 14). (**c**) NSCLC histological subtypes (Supplementary Fig. 16). (**d**) NSCLC stages (Supplementary Fig. 17). (**e**) NSCLC grades (Supplementary Fig. 18). (**f**) Ovarian cancer stages (Supplementary Fig. 21). (**g**) Ovarian cancer grades (Supplementary Fig. 22).

To evaluate whether CES has prognostic values independent from established clinicopathological factors, we carried out multivariate Cox regression using breast cancer and lung cancer data that contain gene expression and clinical information (Supplementary Tables 12–14). Individual data sets and meta-data showed that the prognostic value of the CES remained significant after adjusting for other factors, including tumour stage and individual staging factors, tumour grade, breast cancer ER status, HER2 status, and NSCLC histological subtype (Figs 3g and 4h; Supplementary Tables 15–27). Furthermore, Kaplan–Meier survival analysis after patient stratification according to various factors, including breast cancer molecular subtype and NSCLC histological subtype, demonstrated that high CES remains prognostic for poor patient survival in the majority of cases, although there were several exceptions that we explore below (see Fig. 5 and Supplementary Figs 12–15 for breast cancer, Supplementary Figs 16–20 for NSCLC and Supplementary Figs 21–22 for ovarian cancer). Using TCGA RNA sequencing data, we found that the CES signature significantly prognosticates patient survival for lung ADCs, but not for breast ADC or lung SCCs (Supplementary Fig. 23; Supplementary Table 28). However, the TCGA data sets currently suffer from short follow-up time or lack of treatment information (Supplementary Tables 12–13). Detailed analyses are presented in Supplementary Note 4.

### CES predicts sensitivity to Topo I inhibitors in cell lines

**Figure 6:**
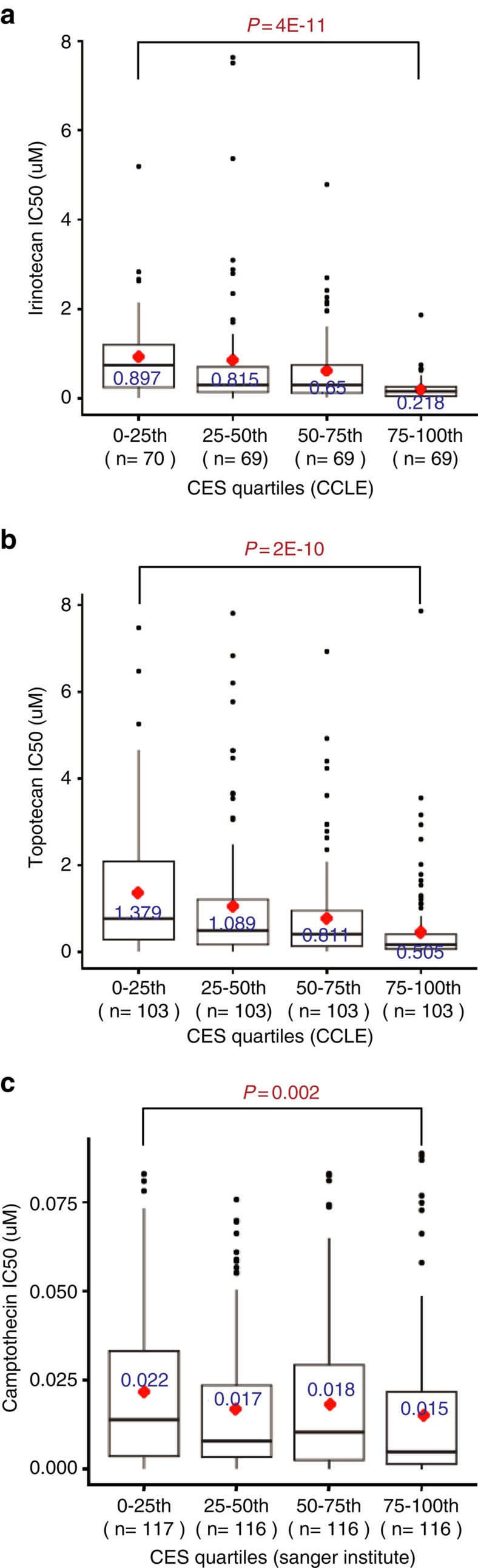
High CES values correlate with increased sensitivity to Topoisomerase I inhibitors in cancer cell lines. Box plots of IC_50_s for Topo I inhibitors in cancer cell lines grouped by CES quartiles. Cell lines in the top CES quartile (75–100th) are significantly more sensitive to Topo I inhibitors than those in the bottom CES quartile (0–25th) determined by Wilcoxon rank-sum tests. Significant *P* values are indicated in red. (**a**) Irinotecan in CCLE data set. (**b**) Topotecan in CCLE data set. (**c**) Camptothecin in Wellcome Trust Sanger Institute CGP data set.

Since high CES cancer cells experience more severe genotoxic stress (Table 1), they may be more sensitive to additional DNA damage than low CES cancer cells. Thus, we mined the Cancer Cell Line Encyclopedia (CCLE) data to investigate the relationship between cancer cell line CES values and half maximal inhibitory concentrations (IC_50_s) for the Topo I inhibitors irinotecan and topotecan, which are camptothecin analogues and damage DNA^50^. After binning CCLE cell lines into quartiles according to their CES values, we detected extremely significant differences in drug IC_50_ between the top and bottom CES quartiles for both irinotecan and topotecan (*P*<0.0001, Wilcoxon rank-sum test; Fig. 6a,b; Supplementary Fig. 24). Consistently, the CES values and drug IC_50_s of cancer cell lines were inversely and significantly correlated (Spearman's *rho*, *r*_s_=−0.384, *P*<0.001 for irinotecan; and *r*_s_=−0.339, *P*<0.001 for topotecan; Table 3). This strong negative correlation was also found across several subgroups of CCLE cell lines derived from specific tissues, including breast, lung and ovarian cancer cell lines (Table 3). The inverse correlation was further confirmed by analysing the Sanger Institute cancer cell line camptothecin data set (Fig. 6c). We conclude that high CES cancer cell lines are more sensitive than low CES cell lines to Topo I inhibitors, which cause genotoxicity and reduce cell survival—consistent with the hypothesis that high CES scores correlate with reduced tolerance to genotoxic stress.

### CES predicts patient outcome after adjuvant chemotherapy

**Figure 7:**
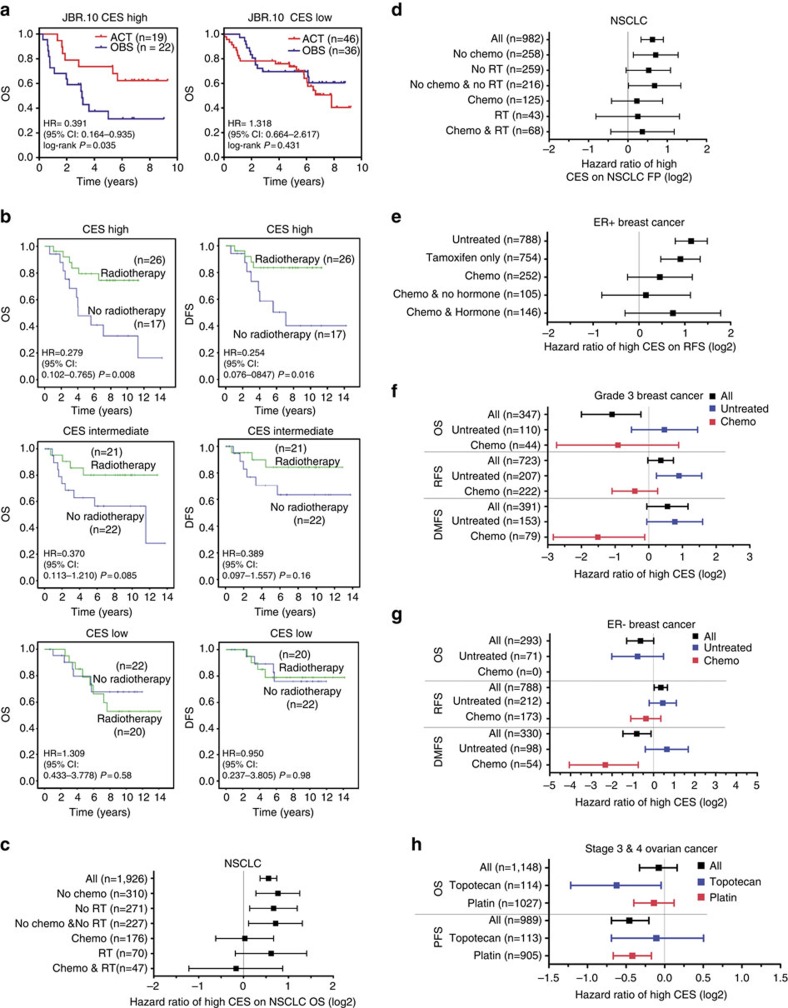
CES predicts cancer patient outcome after adjuvant chemotherapy or radiotherapy. (**a**) Kaplan–Meier plots showing that high CES predicts better patient outcome after adjuvant chemotherapy (ACT) for early-stage NSCLC patients in JBR.10 trial. ACT significantly improved overall survival compared to no ACT (OBS) specifically for the high CES group (top tertile), but not for the low CES group (lower two tertiles). (**b**) Kaplan–Meier plots showing that high CES predicts better patient outcome after adjuvant radiotherapy (RT) for (left) overall survival (OS) and (right) disease-free survival (DFS) for breast cancer patients using the Gray data set (E-TABM-158). Patient cohort was divided into CES tertiles. There is no significant survival benefit from RT for patients with intermediate (middle) and low (bottom) CES. (**c**–**h**) Forest plots summarizing treatment-specific hazard ratios of high CES in cancer patient cohorts using K–M Plotter. Squares and error bars in the plots denote log2 scales of hazard ratio (HR) and 95% confidence interval (CI), respectively. (**c**) NSCLC patient overall survival (OS) with or without chemotherapy or radiotherapy (see Supplementary Fig. 27 for Kaplan–Meier plots). (**d**) NSCLC patient first progression (FP) (Supplementary Fig. 29). (**e**) ER^+^ breast cancer patient relapse-free survival (RFS) with or without tamoxifen or chemotherapy (Supplementary Fig. 30). (**f**) Grade 3 breast cancer patient survival with or without chemotherapy (Supplementary Fig. 31). (**g**) ER^−^ cancer patient survival (Supplementary Fig. 32). (**h**) Stage 3 and 4 combined ovarian cancer patient overall survival and progression-free survival (PFS) with topotecan or platinum treatments (Supplementary Fig. 33).

From the results above, we conjectured that patients with high CES tumours are more likely to respond positively to adjuvant genotoxic therapies, such as chemotherapy or radiotherapy. We first explored this hypothesis by determining whether adjuvant chemotherapy is more effective for early-stage NSCLC patients with high CES tumours, using the JBR.10 early-stage lung cancer clinical trial data set (GSE14814)^51^. This clinical trial utilized a prospective, randomized design, thus avoiding many drawbacks associated with retrospective studies. Post-surgery stage I and II NSCLC patients were randomly assigned to no adjuvant treatment (OBS) or to adjuvant chemotherapy (ACT) including cisplatin, which causes DNA damage and promotes apoptosis, and vinorelbine, a microtubule inhibitor^51^. We divided patients into CES high (top tertile) and low (lower two tertiles) groups, and observed that high CES predicted poor overall survival for patients without adjuvant treatment (hazard ratio (HR)=2.728, *P*=0.017), validating the prognostic power of the CES system (Supplementary Fig. 25A). Importantly, adjuvant chemotherapy effectively negated the adverse outcome associated with high CES (HR=0.710, *P*=0.402), suggesting that the CES system also has predictive power (Supplementary Fig. 25B). Indeed, adjuvant therapy significantly improved overall survival for high CES patients compared with no treatment (HR=0.391, log-rank *P*=0.035; Fig. 7a). Kaplan–Meier analysis demonstrated that this effect was specific for high CES patients, since there was no significant benefit associated with adjuvant therapy for the low CES group (HR=1.318, log-rank *P*=0.431; Fig. 7b). Moreover, multivariate Cox regression analysis on patient subcohorts stratified by CES or treatment options confirmed the prognostic and predictive value of the CES system for early-stage NSCLC patients (Supplementary Tables 24–25). Analysis combining the NSCLC UT SPORE and JBR.10 data sets further strengthened these conclusions (Supplementary Fig. 26; Supplementary Note 5), showing that adjuvant therapy specifically improved 5-year survival for high CES, early-stage NSCLC patients (81.5% for ACT subcohort versus 47.3% for OBS subcohort, *P*=0.002), but not for the low CES group (74.4% for ACT versus 68.4% for OBS, *P*=0.347). We conclude that the CES system effectively predicts patient sensitivity to adjuvant cisplatin chemotherapy for early-stage NSCLC.

To address potential issues associated with small sample sizes in predicting drug sensitivity, we also performed meta-analyses on chemo-sensitivity using K–M Plotter for several cancer types. For both stage I NSCLCs as well as NSCLCs with all stages included, high CES predicted poor survival for patients who did not receive chemotherapy, but not for those who received chemotherapy (Fig. 7c,d; Supplementary Figs. 27–29). These analyses confirmed the results from the JBR.10 clinical trial data.

We next investigated CES values in ER^+^ breast cancer patients, where many patients suffer from relapse after endocrine therapy^52^. We found that high CES associates with poor relapse-free survival for patients who did not receive systemic therapy (HR=2.20, *P*=6.5E-11), confirming the prognostic value of high CES in these patients (Fig. 7e; Supplementary Fig. 30). Moreover, CES is also prognostic for ER^+^ patients treated with tamoxifen only (HR=1.87, *P*=2.6E-05), suggesting that genomic instability is an important mechanism contributing to relapse among those patients. However, high CES lost its significant prognostic value for ER^+^ patients who had chemotherapy, suggesting that chemotherapy reduced the risk of relapse for patients with high CES ER^+^ tumours relative to those with low CES ER^+^ tumours, and consistent with our hypothesis that high CES values predict improved patient response to adjuvant chemotherapy.

In some breast cancer patient cohorts such as high-grade, ER^−^ or basal-like and HER2 subtypes, high CES either: (1) did not have significant prognostic value or (2) predicted better survival (Fig. 5; Supplementary Figs 12–14). Notably, these cohorts are enriched for tumours with high CES values (Table 2). We suspected that this unusual relationship between CES and patient survival stemmed from the sensitivity of high CES tumours to the genotoxic stress added by the treatment. To investigate this hypothesis, we explored high-grade (that is, grade 3) breast cancer cohorts with relatively large sample numbers. The grade 3 cohort displayed higher median and average CES values than lower-grade breast cancers (Table 2; Supplementary Fig. 3). In untreated grade 3 patients, there was a trend indicating that high CES remained a risk factor for poor patient survival with or without significance. However, in treated grade 3 patients, those with high CES showed better survival than those with lower CES (Fig. 7f; Supplementary Fig. 31). Thus, among breast cancer patients with high-grade tumours who were treated by adjuvant chemotherapy, those with the highest CES values were associated with better survival. This relationship between CES values and patient survival was also evident in ER^−^ breast cancers (mainly basal-like and HER2^+^ subtypes) (Fig. 7g; Supplementary Fig. 32), as well as in lung SCC or stage II NSCLC patients that are enriched for high CES tumours (Fig. 5c,d; Supplementary Figs 16 and 17). Moreover, the improved overall survival associated with high CES in lymph node-positive breast cancer patients is probably because there was significant enrichment for high grade (*P*=0.003 for GSE3494 and *P*<0.001 for GSE20711 (ref. 53), respectively, Fisher's exact test) or ER^−^ (GSE16446 (ref. 54) only studied ER^−^ tumours) in lymph node-positive tumours in the 3 data sets used by the K–M Plotter database, and because all patients in the lymph node-positive cohort were subjected to adjuvant therapies (Supplementary Fig. 15B). Importantly, we found no evidence that high CES is associated with significantly better survival without therapy. Altogether, our results suggest that the correlation between extreme genomic instability and improved survival likely results from the greater impact of genotoxic therapy on high CES tumours, and not from high levels of CIN *per se*.

We also studied the CES in ovarian cancer, where most patients had higher-grade (grade 2 and 3) and late-stage (III and IV) tumours, and were treated with chemotherapy including platinum agents. Here high CES appeared to have either: (1) no effect, or (2) was associated with better outcomes, for cohorts including all late-stage patients or platinum-treated late-stage patients (Figs 5f,g and 7h; Supplementary Figs. 21–22 and 33). In addition, high CES was associated with even better overall survival among patients treated with topotecan (HR=0.63, *P*=0.029; Fig. 7h; Supplementary Fig. 33B). Notably, in CCLE ovarian cancer cell lines, high CES values were significantly correlated with increased topotecan sensitivity (Spearman's rho, *r*_s_=−0.469, *P*=0.018; Table 3). Overall, our results demonstrate that the CES system effectively predicts clinical response to adjuvant chemotherapies for lung, breast and ovarian cancer patients.

### CES predicts patient outcome after adjuvant radiotherapy

To determine whether this prediction of patient response extends to other genotoxic cancer therapies, we explored CES values for cancer patients undergoing radiation therapy (RT). This treatment exerts significant genotoxic stress, primarily through double-stranded DNA breaks and other types of damage, and causes apoptosis. As it is not a chemical agent, it constitutes an alternative test of our hypothesis that high CES scores are associated with increased vulnerability to genotoxic stress. Using breast cancer Gray data set^42^, we found that patients with high CES values displayed improved overall survival (HR=0.279, *P*=0.008) and disease-free survival (DFS; HR=0.254, *P*=0.016) after RT, compared with patients not treated with RT (Fig. 7b). Importantly, in contrast, patients with low CES values showed no survival benefit with RT (HR=1.309, *P*=0.58 for OS, and HR=0.950, *P*=0.98 for DFS). The intermediate CES patient group associated with intermediate hazards (HR=0.370, *P*=0.085 for OS, and HR=0.389, *P*=0.16 for DFS). Further analyses showed that the association between RT and improved prognosis is specific to the high CES patient group (Supplementary Fig. 34). Moreover, multivariate Cox regression confirms the significant benefit of RT for high CES breast cancer patients (Supplementary Table 20). Meta-data analysis further indicates that RT treatment significantly reduces the hazard of high CES for NSCLC patients (Fig. 7c,d; Supplementary Figs 27 and 29). Thus, the CES system can predict cancer patient outcome after both adjuvant chemotherapy and RT.

## Discussion

In this study, we used a hypothesis-driven approach to interrogate the prognostic and predictive value of centromere and kinetochore protein gene misexpression in human cancers. Analyses of numerous cancer databases demonstrate that 14 CEN/KT genes are consistently overexpressed in a wide spectrum of human cancer types and prognosticate patient survival. Many of these 14 CEN/KT genes are involved in the process of CENP-A nucleosome assembly, supporting its potential importance in cancer progression. To summarize the extent of CEN/KT gene overexpression, we developed a CES (for Centromere and kinetochore gene Expression Score) signature. We show that high human tumour CES values are associated with several adverse tumour properties, and predict poor patient outcomes, including locoregional recurrence, metastatic spread and overall survival, independently from established clinicopathological factors. High CES values significantly correlate with increased levels of genomic instability (fraction of genome with CNAs and mutation frequencies) in many different cancer types. Thus, we hypothesized that tumours with very high CES are more sensitive to further DNA damage, which is supported by the observation that high CES cancer cell lines demonstrate increased sensitivity and cytotoxicity to topoisomerase I inhibitors. We further show that the CES signature effectively predicts outcomes in breast and lung cancer patients receiving adjuvant chemotherapy or radiotherapy. Thus, we conclude that the CES signature is an effective prognostic and predictive biomarker.

We propose that clinical implementation of the CES signature could contribute to ‘precision medicine' by allowing more effective therapeutic regimens to be chosen, which would limit patient exposure to less effective therapies^55^. For example, many early-stage lung cancer patients who receive adjuvant chemotherapy after resection experience severe side effects without significant benefit^56^. Consequently, the CES signature may hold clinical value by separating responders from non-responders to first-line adjuvant therapies that include platinum agents. Such separation could help spare non-responders from the toxicity of unbeneficial therapy, and, importantly, would promote exploration of other potentially more effective therapeutic regimens for this population. CES may offer similar clinical value in breast DCIS. The CES marker may help identify patients at risk for recurrence, especially invasive recurrences, as only a subset of breast DCIS display high CEN/KT gene expression. This would allow potential de-escalation of adjuvant therapy in low-risk patients who are unlikely to receive significant benefit^57^. Validating the CES marker's utility in identification of high-risk DCIS will require analysing a larger number of patients in prospective data sets. Importantly, since centromeres are universally required for genome stability, the CES signature can potentially be useful for cancer types other than those examined in this study.

Our results may help elucidate the role of centromere misregulation in cancer progression and genome instability. Half the 14 CES genes are involved in the assembly of CENP-A nucleosomes, which is the structural foundation for centromere propagation and function. Therefore, misregulation of centromere replenishment may be a key mechanism that drives genome instability in cancer. This idea is supported by the observation that perturbing CENP-A nucleosome assembly in model organisms and human cultured cells produces severe mitotic defects^32^,^33^,^58^,^59^. Overexpression or mislocalization of key centromere proteins generates dicentric or lagging chromosomes and other segregation errors^22^,^39^,^60^. These mitotic errors also increase levels of DNA damage due to spindle-mediated fragmentation of dicentric chromosomes, ‘cutting' of lagging chromosomes in cytokinesis or defective DNA repair associated with micronuclei in the following cell cycle^20^,^34^,^39^,^40^,^41^,^61^. Consistently, high tumour CES values were associated with several adverse tumour characteristics and with increased risk of relapse and metastasis in patients, suggesting a role for genomic instability associated with high CES in cancer progression.

Some studies using yeast, human cancer cell lines or mouse tumour models have shown that genomic instability or aneuploidy results in poor viability and reduced growth of normal cells, or tumour cells not subjected to therapeutic intervention^62^,^63^,^64^. These studies using experimental models differ from our analyses using human clinical data and patient survival as the end point readout. Importantly, we do not find clinical evidence that extreme genomic instability benefits patient survival without genotoxic therapy. Our results on cell line drug sensitivity and patient outcome on adjuvant therapies indicate that the elevated levels of genomic instability sensitize cancer cells to be susceptible to genotoxic agents. A simple explanation for these findings is that therapy elevates genomic perturbations to a level that cannot be effectively repaired, resulting in cancer cell death^65^. This provides an explanation for the unusual correlation between extremely high CES values and improved patient survival observed in patient cohorts enriched for high CES tumours that undergo adjuvant therapies. Our data may also help interpret the results from a recent prospective breast cancer clinical trial (TACT) where high levels of CIN (measured by fluorescence *in situ* hybridization) were associated with improved patient survival for ER^−^ breast cancer^66^. Importantly, in that study all ER^−^ patients were subjected to adjuvant therapies, and in our study we found no evidence that high CES is associated with significantly better patient survival without therapy. Thus, extreme genomic instability in tumour cells may associate with improved survival only for patients treated with adjuvant genotoxic therapies, a hypothesis that merits further investigation. However, this predictive power of the CES signature appears to also partially temper its prognostic value for certain tumour types such as high-grade, ER^−^ or basal-like and HER2^+^ breast cancers.

In addition to this ‘CIN threshold' model, the involvement of some CES genes in DNA repair could provide another explanation for the sensitivity of high CES cancers to further DNA damage. The CENP-A chaperone and assembly factor HJURP (for ‘Holliday Junction Recognition Protein') was shown to regulate DNA repair and cell viability in cancer cell lines after radiation^42^,^67^. The role of CENP-A in DNA repair has also been reported but may depend on genetic and cellular contexts^68^,^69^. Other CES proteins also may regulate DNA repair. For example, *CENP-W* is a CES gene and member of the CENP-T/-W/-S/-X complex. CENP-S/CENP-X is also known as the MHF complex and stimulates replication fork remodelling by FANCM in DNA repair^70^. It is thus conceivable that overexpression of *CENP-W* favors formation of the CENP-T/-W/-S/-X complex and depletes the pool of CENP-S/CENP-X available for DNA repair or replication. If CES gene overexpression enhances genome instability through centromere misregulation, and simultaneously suppresses effective DNA repair, the CES signature could potentially identify patients who are extremely sensitive to further DNA damage. In future research, it will be interesting to determine whether the CES genes act synergistically with other genes involved in genome maintenance, such as *BRCA1* and *BRCA2* (refs 71, 72), to promote cancer progression.

Finally, one proposed strategy for cancer therapy involves specifically killing cells that contain chromosome aberrations^13^. Here we have identified a group of centromere and kinetochore protein genes whose levels of expression strongly correlate with cancer patient outcome and sensitivity to therapies. These chromosomal functions are distinct from many existing drug targets involved in signal transduction and in regulation of oncogenic or tumour suppression pathways. Thus, these CEN/KT proteins could provide novel drug targets that help overcome the drug resistance caused by CIN, and may increase the effectiveness of cancer cell responses when combined with therapies that target signal transduction or other known oncogenic or tumour suppression pathways.

## Methods

### Data sets used in this study

For identifying differentially expressed CEN/KT genes, 13 microarray data sets containing both normal and tumour samples were downloaded from the GEO website. Sample characteristics are summarized in Supplementary Table 1 for the data sets used for comparison between normal and tumour tissues by gene expression heat maps, differentially expressed gene analysis and permutation test.

Individual breast cancer and lung cancer data sets containing gene expression, clinical information, treatment information and survival data are summarized in Supplementary Tables 12 and 13 for Kaplan–Meier survival estimations, correlation study and multivariate Cox regression analysis after removing samples of missing information. The Affymetrix probes for the 14 CES genes are listed in Supplementary Table 30. For correlations between CES and breast cancer ER and PR status, and molecular subtype, GSE47561 data set was used^73^, which is a third party re-analysis of the meta-data set containing GSE2034, GSE11121, GSE20194, GSE1456, GSE2603, GSE6532, GSE20437, GSE1561, GSE7390 and GSE5847. Normalized UT SPORE NSCLC data set GSE42127 was generously provided by Drs Yang Xie and Hao Tang^74^. Data sets used for Kaplan–Meier meta-analysis (using K–M plotter) of breast (*N*=4,141), lung (*N*=2,438), ovarian (*N*=1,638) and gastric (*N*=751) cancers are listed in Supplementary Table 14.

For TCGA data sets, mRNA expression, fraction of CNA, frequency of non-synonymous gene mutations in cancer exomes and patient clinical information for the set of samples were downloaded from cBioPortal^48^. For the analyses of the TCGA breast ADC data set for correlation between CES and tumour features, we removed samples falling into any of the following categories before analysis: low tumour purity (defined by <0.3 unless otherwise specified), patients marked for having had neo-adjuvant therapy, samples of missing clinical data, samples from metastatic tumours and male samples. For the TCGA lung ADC data set, samples were removed for patients with pretreatment or unknown pretreatment history, and patients with missing survival information.

### Gene expression heat maps and co-expression network

For heat maps, sample clustering was performed on the CEN/KT gene expression profiles using hierarchical clustering in Cluster 3.0 and Java Treeview 1.1.6r4 to group samples by centroid. Gene co-expression correlation networks were constructed for each cancer type using TCGA data sets downloaded from the cBioPortal^48^. A network of CEN/KT genes was constructed using Cytoscape 2.8.0 (www.cytoscape.org) with the ExpressionCorrelation plugin (http://baderlab.org/Software/ExpressionCorrelation)^75^. Correlation coefficients exceeding a threshold (*R*⩾0.4) were displayed as edges between genes represented by nodes. Nodes with fewer edges were arranged to the left of the network and those with more edges to the right.

### Kaplan–Meier plots and multivariate Cox regression analysis

For individual breast cancer and lung cancer data sets, Kaplan–Meier curves were generated for patients stratified into groups of high (upper tertile), intermediate (middle tertile) and low (lower tertile) CES values. For the NSCLC JBR.10 trial and UT lung SPORE data sets used for chemotherapy outcome prediction, the CES high patient group consists of the top CES tertile, and the remaining two tertiles are defined as CES low. Kaplan–Meier survival analysis and multivariate Cox regression were performed in SPSS or R. R code and the individual breast cancer and lung cancer data sets for the Kaplan–Meier plots and multivariate Cox regression in Figs 3 and 4 are available as Supplementary Software 1. The top CES tertile and lower two CES tertiles in the NSCLC JBR.10 and UT SPORE data sets were pooled for the Kaplan–Meier survival analysis of sensitivity to adjuvant chemotherapy. Five-year survival for lung cancer patients was analysed by *χ*^2^-test for significance.

We performed meta-analysis for breast cancer any event survival and metastatic relapse-free survival on 17 breast cancer data sets using bc-GenExMiner v3.0, and for breast, lung, gastric and ovarian cancers using K–M Plotter. For meta-analysis of the prognostic value of the CES for each cancer type and subcohorts using the K–M Plotter database, we used automatically computed best CES thresholds to detect the most significant difference between high and low CES groups, after stratifying patients according to different clinicopathological factors using K–M Plotter. For meta-analysis of sensitivity to adjuvant therapies using K–M plotter, we used the top CES tertile as CES high, and the remaining two tertiles as CES low; using the automatically computed best performing CES threshold showed similar trends in most cases. The K–M Plotter database incorporates genes with probes present in Affymetrix HG-U133A array to maximize sample sizes and ensure comparability between data sets for meta-analysis, thus it excluded seven CEN/KT genes (*CENP-H*, *-W*, *-L*, *-K*, *-P*, *SPC24* and *NUF2*), five of which are also CES genes (*CENP-W*, *-L*, *-K*, *SPC24* and *NUF2*). Therefore, for K–M Plotter analyses we used the nine remaining CES genes as a simplified version of the CES signature to maximize sample sizes. Cohorts with small sample sizes (*n*<30) were excluded from meta-data analysis using K–M Plotter. K–M Plotter Database was accessed in October 2015.

### Statistical analysis

The Statistical Analysis of Microarrays Excel add-on package (http://www-stat.stanford.edu/~tibs/SAM/) was used to identify differences between normal and tumour tissues in expression levels of CEN/KT genes using the following criteria (FDR *P*≤0.05, fold changes ⩾2, and in at least 50% (as the empirical prevalence cutoff threshold) data sets examined. For identification of differentially expressed CEN/KT gene probes in Supplementary Data 1, all microarray data were determined to be normally distributed. Permutation tests were performed in R to confirm significant overexpression of CES genes identified by the DE gene analysis. Statistical analyses were performed using the Statistical Package for the Social Sciences version 11.5 (SPSS, Inc., Chicago, IL), Graphpad Prism, or R version 3.0.2.

Significant associations between CES values and clinicopathological factors were evaluated by a Wilcoxon rank-sum test for two-group comparison and a Kruskal–Wallis test for multiple groups. For CCLE and CGP drug sensitivity data, significant differences in IC_50_s between the top and bottom CES quartiles were determined by a Wilcoxon rank-sum test. For Sanger Institute CGP cell line drug sensitivity to camptothecin, samples with extreme IC_50_ values were excluded from the analysis. Extreme IC_50_s were defined by three times of a robust location-free scale estimate above the median that is more efficient than median absolute deviation, and is more resistant to extreme or outlier data points than 3 s.d. above the population mean^76^.

### Data availability

All microarray data sets mined in this study are available from GEO database at National Center for Biotechnology Information (http://www.ncbi.nlm.nih.gov/gds/) except for two data sets. The Joe Gray E-TABM-158 breast cancer data set is available from ArrayExpress at EMBL-EBI (http://www.ebi.ac.uk/arrayexpress/experiments/E-TABM-158/)^77^. The breast cancer NKI data set is accessible at Dr Howard Chang's laboratory website at Stanford University (http://changlab.stanford.edu/2005-PNAS-Data.html)^78^.

Kaplan–Meier survival estimation by meta-analysis that support the findings of this study are available from bc-GenExMiner (http://bcgenex.centregauducheau.fr/BC-GEM/GEM_Accueil.php?js=1) and K–M Plotter (http://kmplot.com/analysis/) following their respective query tutorials^45^,^79^.

All TCGA RNA-seq data and associated clinical information are available from cBioPortal (http://www.cbioportal.org/)^48^.

The CCLE drug sensitivity and gene expression data are available from Broad Institute (http://www.broadinstitute.org/ccle/home), and the Cancer Genome Project drug sensitivity and gene expression data are available from the Sanger Institute (http://www.cancerrxgene.org/downloads/), respectively^50^,^80^. All other data are contained within the article and the Supplementary Information files, or available from the author on request.

## Additional information

**How to cite this article:** Zhang, W. *et al*. Centromere and kinetochore gene misexpression predicts cancer patient survival and response to radiotherapy and chemotherapy. *Nat. Commun.* 7:12619 doi: 10.1038/ncomms12619 (2016).

## Supplementary Material

Supplementary Information Supplementary Figures 1-34, Supplementary Tables 1-30, Supplementary Notes 1-5 and Supplementary References.

Supplementary Data 1 Differential expression of 15 CEN/KT genes is significant in cancer progression across cancer types.

Supplementary Software 1 R code and the individual breast cancer and lung cancer datasets for the Kaplan-Meier plots and forest plots in Figures 3 and 4.

## Figures and Tables

**Table 1:** Correlation between tumour CES values and CNA and mutation frequencies in TCGA data sets.

**Cancer Types**	**Mutation frequency**				**Copy-number alteration**			
	***r***_**s**_	***P*** **value**	**FDR** ***P*** **value**	***N***	***r***_**s**_	***P*** **value**	**FDR** ***P*** **value**	***N***
Adrenocortical carcinoma	0.468	**3.3E-06**	**1.2E-05**	72	0.046	0.703	0.703	72
Bladder urothelial carcinoma	0.240	**0.006**	**0.011**	129	0.244	**3.4E-08**	**8.7E-08**	353
Lower-grade glioma	0.323	**4.8E-08**	**2.2E-07**	273	0.483	**1.5E-30**	**9.0E-30**	499
Breast adenocarcinoma	0.443	**3.7E-48**	**6.7E-47**	975	0.539	**3.4E-82**	**6.1E-81**	1,076
Cervical SCC and endocervical adenocarcinoma	0.095	0.191	0.264	191	0.091	0.155	0.186	244
Colorectal carcinoma	0.145	0.050	0.075	182	−0.031	0.674	0.703	182
Glioblastoma	0.090	0.280	0.336	147	0.128	0.120	0.166	148
Head and neck SCC	0.124	**0.031**	0.051	304	0.208	**3.2E-06**	**7.2E-06**	494
Kidney RCC	0.036	0.466	0.493	410	0.179	**4.7E-05**	**9.4E-05**	513
Kidney RPC	0.049	0.542	0.542	121	0.182	**0.002**	**0.004**	276
Lung ADC	0.338	**6.3E-06**	**1.9E-05**	171	0.290	**7.5E-11**	**2.7E-10**	485
Lung SCC	0.236	**0.002**	**0.003**	178	0.509	**3.6E-34**	**3.2E-33**	498
High-grade ovarian serous cystadenocarcinoma	0.071	0.368	0.414	161	0.133	**0.031**	0.051	262
Prostate adenocarcinoma	0.349	**1.6E-08**	**1.2E-07**	248	0.487	**1.4E-29**	**6.3E-29**	474
Skin cutaneous melanoma	0.196	**2.7E-04**	**6.9E-04**	339	0.066	0.152	0.186	468
Stomach adenocarcinoma	0.386	**2.0E-08**	**1.2E-07**	198	0.377	**2.8E-10**	**8.4E-10**	262
Thyroid carcinoma	0.056	0.273	0.336	391	0.036	0.427	0.480	489
Uterine carcinosarcoma	0.432	**9.0E-04**	**0.002**	56	0.263	0.050	0.075	56

ADC, adenocarcinoma; CNA, copy-number alteration; RCC, renal cell carcinoma; RPC, renal papillary cell carcinoma; SCC, squamous cell carcinoma.

Significant two-tailed *P* values and FDR-adjusted *P* values for Spearman's correlation coefficient (*r*_s_) are bolded (*P*<0.05).

**Table 2:** Distribution of specific tumour characteristics among CES tertiles.

**Clinical factors**	**CES tertile**			***χ***^**2**^-**test**
	**Low (%)**	**Intermediate (%)**	**High (%)**	(***P*** **value)**
**Breast cancer**				
Grade 3*	12	23	65	**5E−36**
ER negative†	8	19	73	**3E−23**
Positive lymph node†	34	35	31	0.682
High stage (III+IV)†	33	35	32	0.899
**NSCLC (ADC and SCC)**				
Positive lymph node‡	24	40	36	**0.047**
High stage (III+IV)‡	26	37	37	0.161
SCC versus ADC§	9	31	60	**1E−14**

ADC, adenocarcinoma; CES, Centromere and kinetochore gene Expression Score; NSCLC, non-small cell lung cancer; SCC, squamous cell carcinoma

Percentages of tumours with specific tumour characteristics among CES tertiles were displayed. Significant *P* values determined by *χ*^2^-test are bolded (*P*<0.05).

^*^Five data sets with grade information listed in Supplementary Table 12 were pooled (GSE6532, GSE1456, GSE3494, NKI and Gray data set).

^†^TCGA breast adenocarcinoma data set with 0.3 tumour purity cutoff. Tumour purity cutoff at 0.7 results in similar conclusions but not shown.

^‡^TCGA lung ADC and SCC data sets were pooled. Only lung SCC data set contains lymph node invasion information.

^§^Three NSCLC data sets containing histological subtype information listed in Supplementary Table 13 were pooled (GSE14814, GSE42127 and GSE37745).

**Table 3:** Correlation between cancer cell line CES values and IC_50_s for irinotecan and topotecan in CCLE cancer cell lines.

**CCLE cell lines**	**Irinotecan**			**Topotecan**		
	**Spearman's** ***rho***	***P*** **value**	***n***	**Spearman's** ***rho***	***P*** **value**	***n***
Breast	−0.644	0.007*	16	−0.247	0.281	21
Lung	−0.419	0.005*	44	−0.425	0.0001*	77
Ovary	−0.623	0.003*	20	−0.469	0.018*	25
Haematopoietic and lymphoid	−0.344	0.017*	48	−0.092	0.468	65
Skin	0.200	0.327	26	−0.163	0.364	33
CNS	−0.203	0.436	17	−0.126	0.568	23
Pancreas	−0.439	0.078	17	−0.143	0.536	21
Pooled	−0.384	<0.000001*	277	−0.339	<0.000001*	412

CCLE, Cancer Cell Line Encyclopedia; CES, Centromere and kinetochore gene Expression Score; IC_50_, half maximal inhibitory concentration.

Significant two-tailed *P* values for Spearman's correlation coefficient *rho* are indicated by asterisks (*P*<0.05).
