# Supplementary material for: Centromere and kinetochore gene misexpression predicts cancer patient survival and response to radiotherapy and chemotherapy
Source: Nat Commun. 2016 Aug 31;7:12619. doi: 10.1038/ncomms12619 (PMC5013662; doi:10.1038/ncomms12619)
Supplement: Supplementary Information — Supplementary Figures 1-34, Supplementary Tables 1-30, Supplementary Notes 1-5 and Supplementary References. [file ncomms12619-s1.pdf]

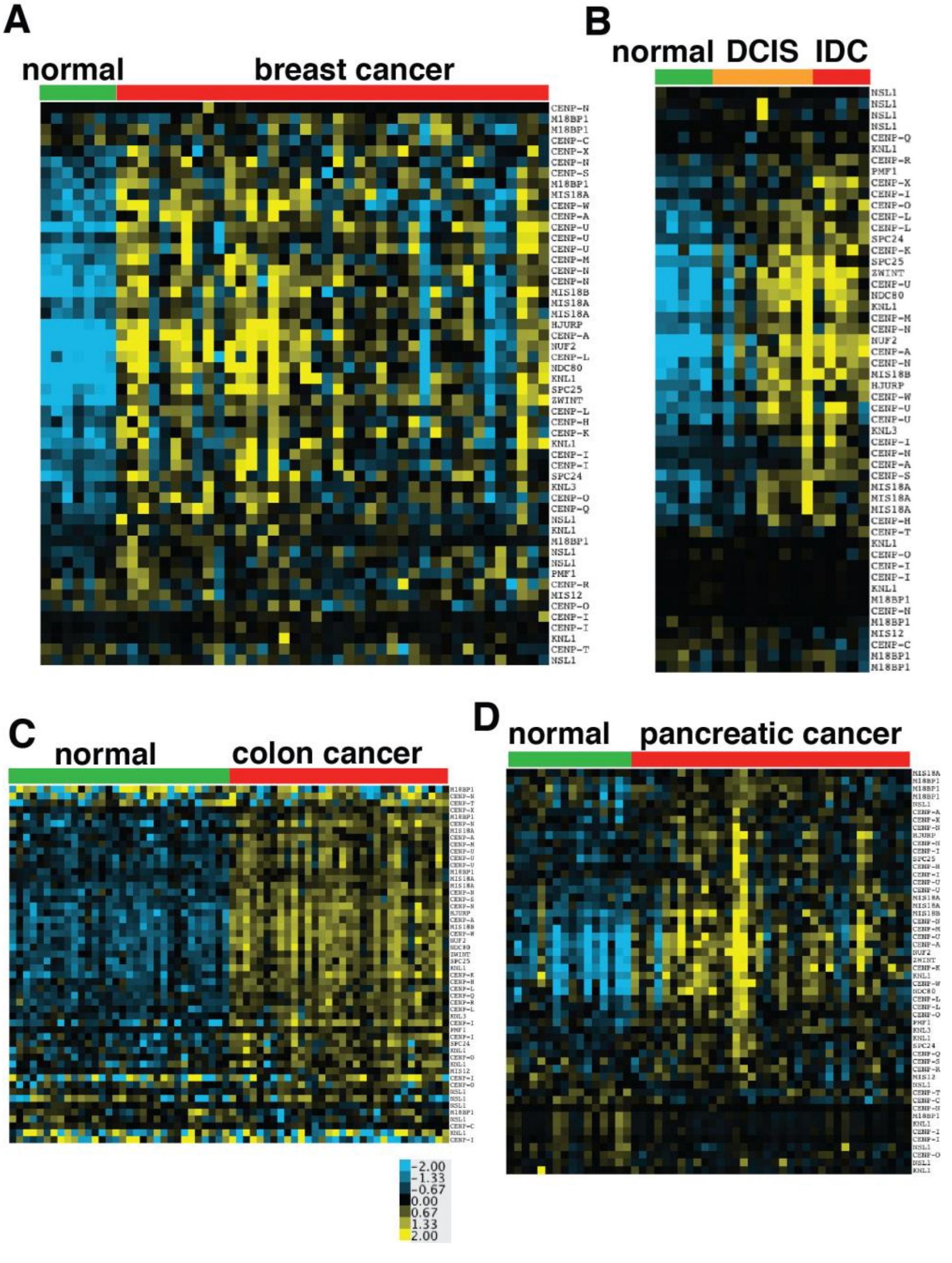
A
normal
breast cancer
B
normal
DCIS
IDC
C
normal
colon cancer
D
normal
pancreatic cancer
-2.00
-1.33
-0.67
0.00
0.67
1.33
2.00

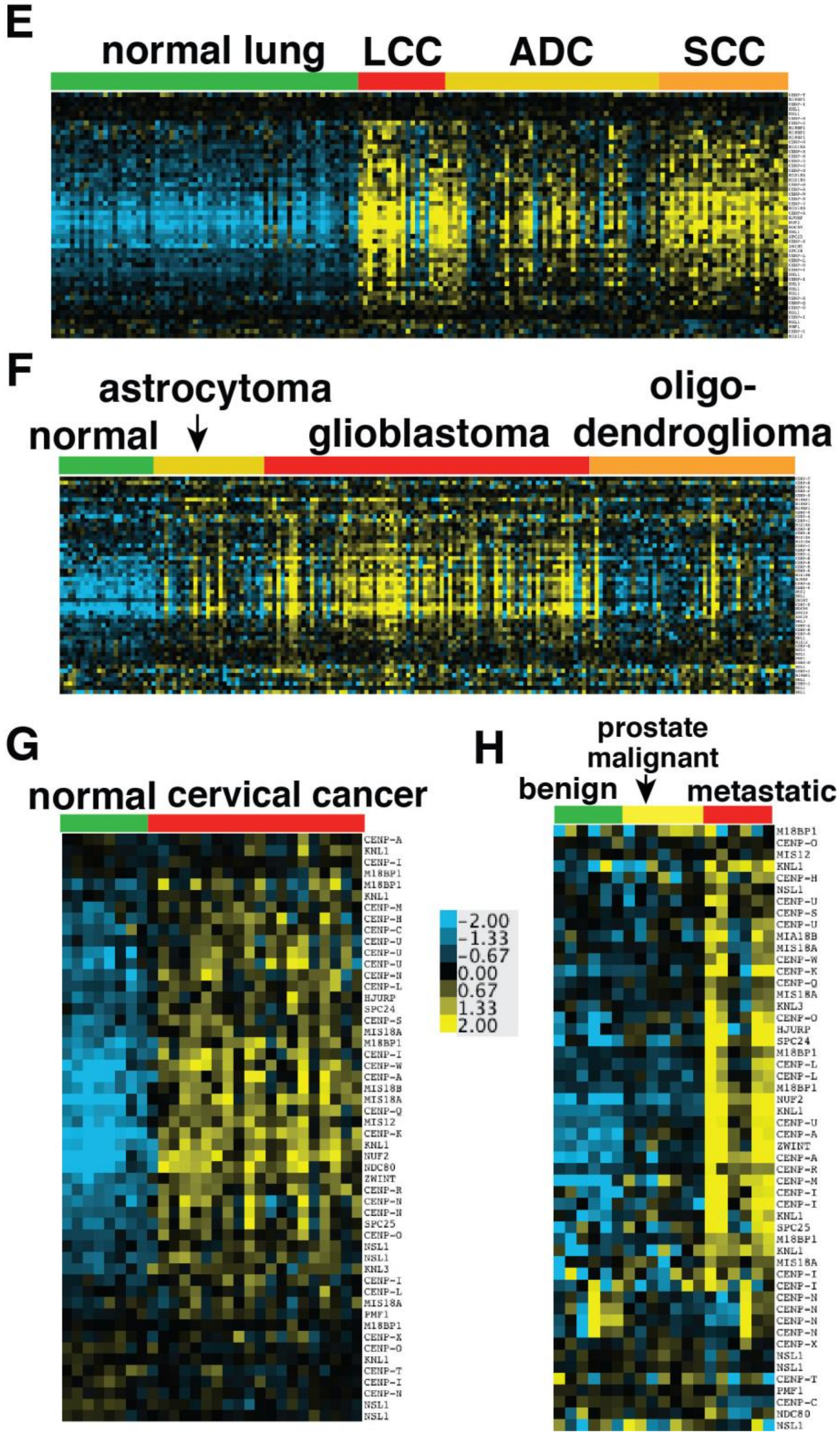
E
normal lung
LCC
ADC
SCC
F
astrocytoma
normal
glioblastoma
oligo-
dendroglioma
G
normal
cervical cancer
CENP-A
KNL1
CENP-I
M18BP1
M18BP1
KNL1
CENP-M
CENP-H
CENP-C
CENP-U
CENP-U
CENP-U
CENP-N
CENP-L
HJURP
SPC24
CENP-S
MIS18A
M18BP1
CENP-I
CENP-W
CENP-A
MIS18B
MIS18A
CENP-Q
MIS12
CENP-K
KNL1
NUF2
NDC80
ZWINT
CENP-R
CENP-N
CENP-N
SPC25
CENP-O
NSL1
NSL1
KNL3
CENP-I
CENP-L
MIS18A
PMF1
M18BP1
CENP-X
CENP-O
KNL1
CENP-T
CENP-I
CENP-N
NSL1
NSL1
-2.00
-1.33
-0.67
0.00
0.67
1.33
2.00
H
prostate
malignant
benign
metastatic
M18BP1
CENP-O
MIS12
KNL1
CENP-H
NSL1
CENP-U
CENP-S
CENP-U
MIA18B
MIS18A
CENP-W
CENP-K
CENP-Q
MIS18A
KNL3
CENP-O
HJURP
SPC24
M18BP1
CENP-L
CENP-L
M18BP1
NUF2
KNL1
CENP-U
CENP-A
ZWINT
CENP-A
CENP-R
CENP-M
CENP-I
CENP-I
KNL1
SPC25
M18BP1
KNL1
MIS18A
CENP-I
CENP-I
CENP-N
CENP-N
CENP-N
CENP-N
CENP-X
NSL1
NSL1
CENP-T
PMF1
CENP-C
NDC80
NSL1

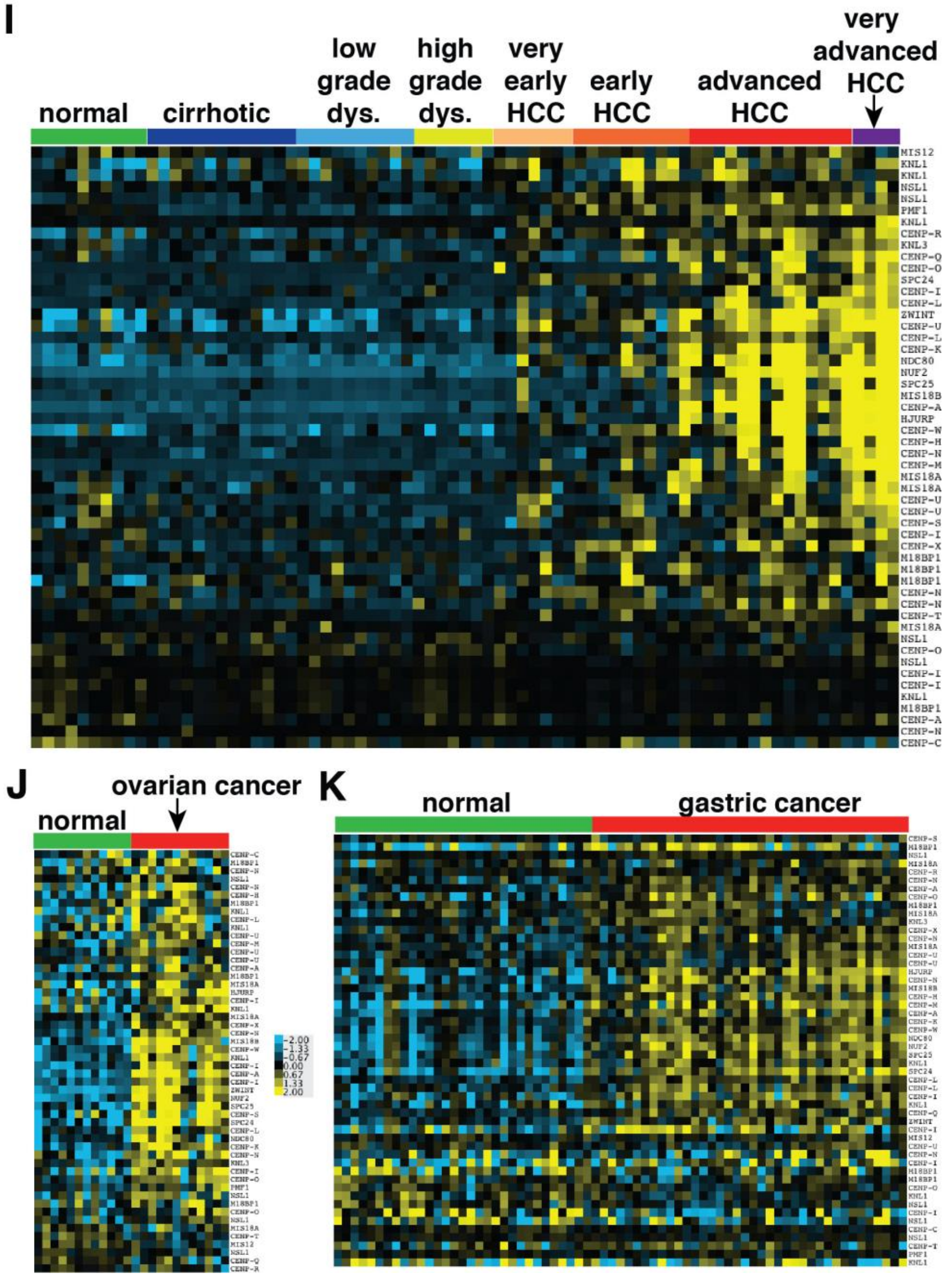
I
normal
cirrhotic
low grade dys.
high grade dys.
very early HCC
early HCC
advanced HCC
very advanced HCC
J
normal
ovarian cancer
K
normal
gastric cancer

L

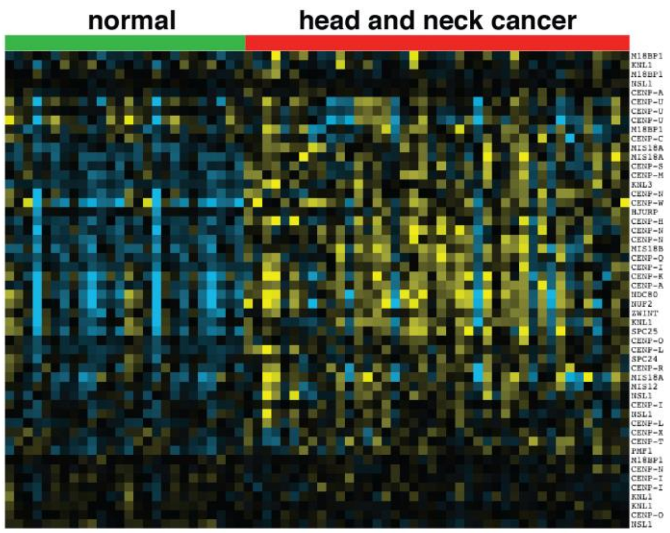

M

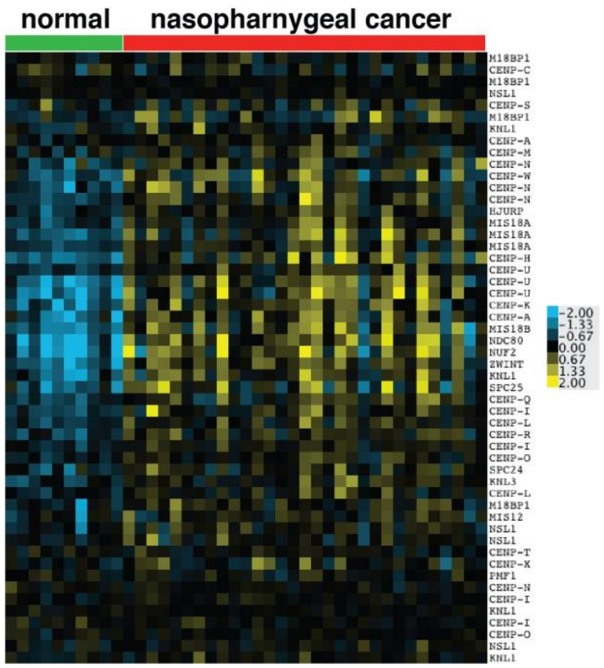

Supplementary Figure 1. CEN/KT genes are misexpressed in various human cancers in comparison to corresponding normal or non-malignant tissues. Samples were clustered according to their CEN/KT gene expression profiles by centroid. Gene names are shown to the right of the heat maps, genes appearing multiple times indicate multiple probes for the same gene. Expression scales are indicated and are identical for all heat maps. (A) breast IDCs (GSE3744). (B) breast DCIS and IDCs (GSE21422). (C) colorectal cancers (GSE8671). (D) pancreatic cancers (GSE16515). (E) lung cancers (GSE19188). (F) brain cancers (GSE4290). (G) cervical cancers (GSE6791). (H) prostate cancers (3325). (I) liver cancers (GSE6764), dys.= dysplastic. (J) ovarian cancers (GSE14407). (K) gastric cancers (GSE13911). (L) head and neck cancers (GSE6791). (M) nasopharyngeal cancers (GSE12452).

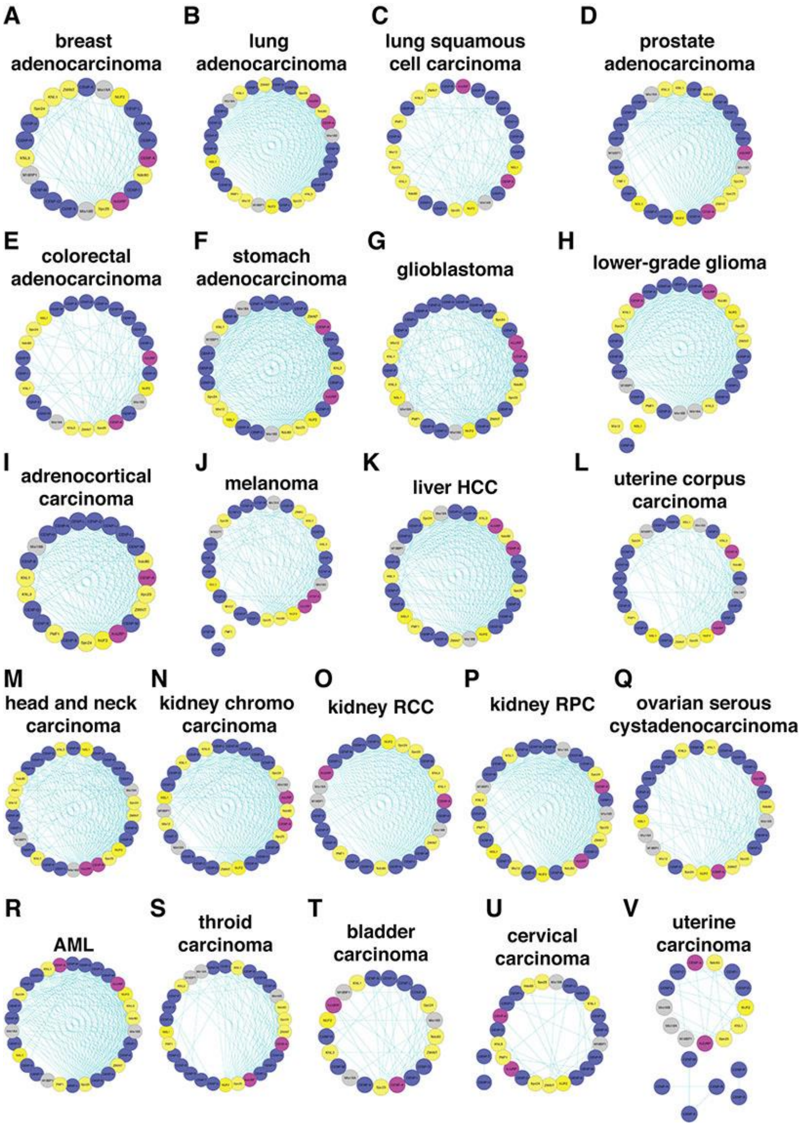

Supplementary Figure 2. Gene co-expression correlation network analyses for CEN/KT genes using TCGA datasets. Individual CEN/KT genes are highlighted according to the color scheme in Figure 1B. Nodes with the highest number of edges are arranged to the right, and those with the lowest number of edges are to the left. (A) breast adenocarcinoma. (B) lung adenocarcinoma. (C) lung squamous cell carcinoma. (D) prostate adenocarcinoma. (E) colorectal adenocarcinoma. (F) stomach adenocarcinoma. (G) glioblastoma. (H) brain lower-grade glioma. (I) adrenocortical carcinoma. (J) melanoma. (K) liver heptacellular carcinoma (HCC). (L) uterine corpus endometrial carcinoma. (M) head and neck squamous cell carcinoma. (N) kidney chromophobe carcinoma. (O) kidney renal clear cell (RCC) carcinoma. (P) kidney renal papillary cell (RPC) carcinoma. (Q) high-grade ovarian serous cystadenocarcinoma. (R) acute myeloid leukemia (AML). (S) thyroid carcinoma. (T) bladder urothelial carcinoma. (U) cervical squamous cell carcinoma and endocervical adenocarcinoma. (V) uterine carcinoma.

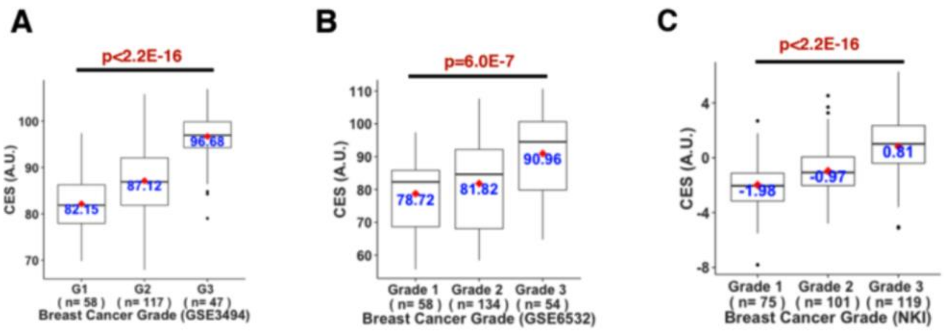

Supplementary Figure 3. **Box plots showing that high CES consistently correlates with high tumor grade.** (A) GSE3494. (B) GSE6532. (C) breast cancer NKI. Mean CES values are labeled (blue) and marked (red diamond). Kruskal-Wallis tests indicate extremely significance (*p*-values labeled in red) for all tested datasets. The thick horizontal line indicates median. The top and the bottom of the box represent the upper and lower quartiles, respectively. The whiskers represent the upper and lower extremes, respectively. Dots indicate outlier data points.

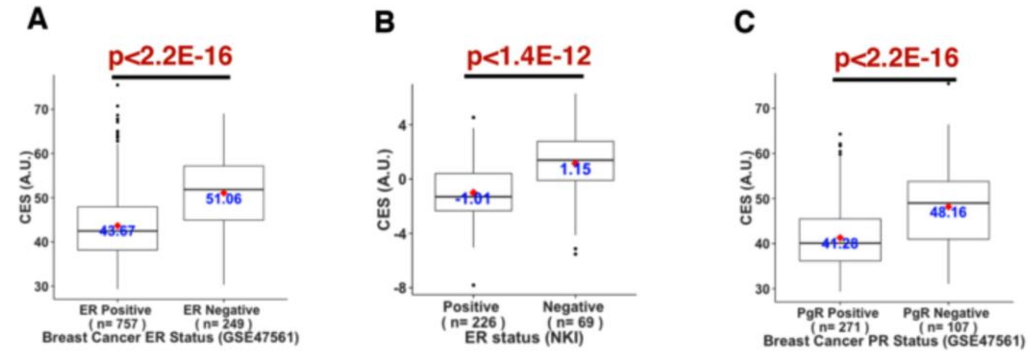

Supplementary Figure 4. Box plots showing that high CES consistently correlates with negative ER and PR status. (A) GSE47561 and (B) breast cancer NKI for negative ER status. (C) GSE47561 for negative PR status. CES means are labeled (blue) and marked (red diamond). Wilcoxon rank-sum tests indicate extremely significant *p*-values (labeled in red). The thick horizontal line indicates median. The top and the bottom of the box represent the upper and lower quartiles, respectively. The whiskers represent the upper and lower extremes, respectively. Dots indicate outlier data points.

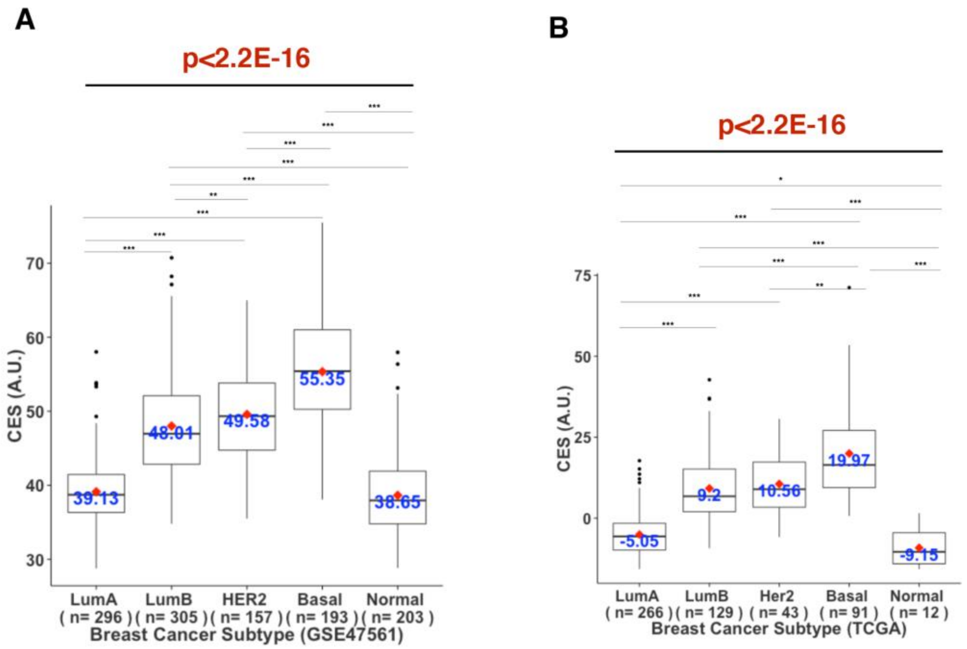

Supplementary Figure 5. Box plots showing that high CES consistently correlates with more aggressive molecular subtypes in breast cancers. (A) GSE47561. (B) TCGA breast adenocarcinoma RNA-seq dataset. LumA=luminal A, LumB=luminal B, Basal=basal-like, Her2 = HER2+, Normal=normal-like. Mean CES values are labeled (blue) and marked (red diamond). Kruskal-Wallis tests indicated extreme significance. Significant pairs using Wilcoxon rank-sum tests are marked with * ($0.01 \leq p < 0.05$), ** ($0.001 \leq p < 0.01$) and *** ($p < 0.001$). The thick horizontal line indicates median. The top and the bottom of the box represent the upper and lower quartiles, respectively. The whiskers represent the upper and lower extremes, respectively. Dots indicate outlier data points.

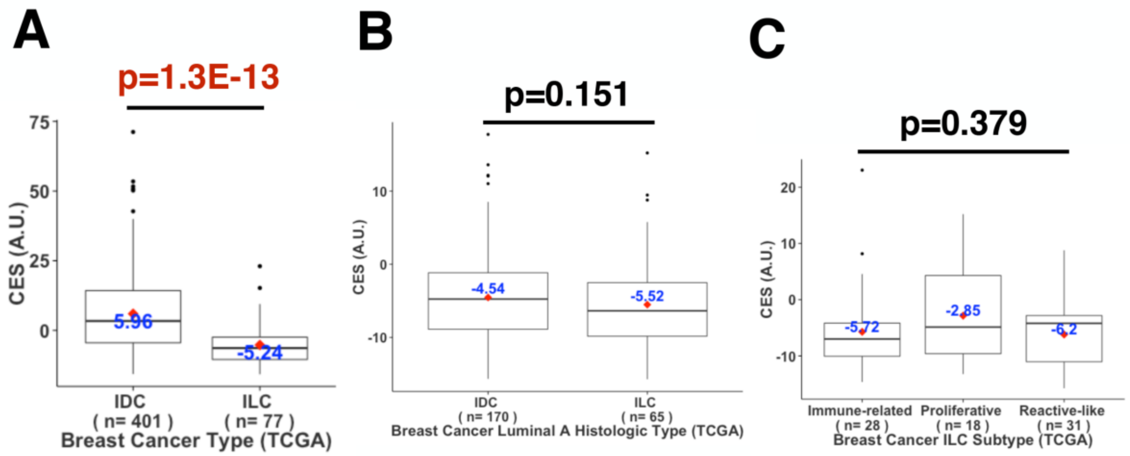

Supplementary Figure 6. Box plots showing correlation between CES and breast adenocarcinoma histologic type using TCGA breast adenocarcinoma dataset. (A) High CES significantly correlates with invasive ductal carcinoma (IDC) compared to invasive lobular carcinoma (ILC). Wilcoxon rank-sum test indicates extreme significance. (B) Within luminal A molecular subtype, there is no significant difference for CES between IDC and ILC by Wilcoxon rank-sum test. (C) There is no significant association between CES and ILC subtype by Kruskal-Wallis test. CES means are labeled (blue) and marked (red diamond). The thick horizontal line indicates median. The top and the bottom of the box represent the upper and lower quartiles, respectively. The whiskers represent the upper and lower extremes, respectively. Dots indicate outlier data points.

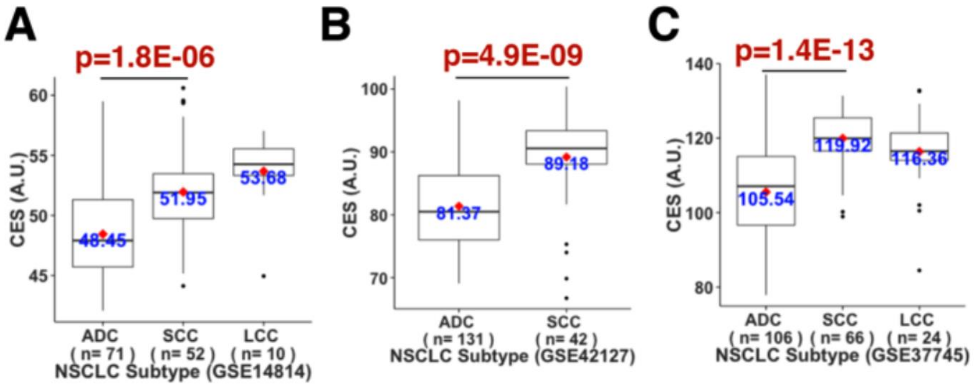

Supplementary Figure 7. Box plots showing that high CES correlates with lung squamous cell carcinoma (SCC) compared to lung adenocarcinoma (ADC) in NSCLC. (A) GSE14814. (B) GSE42127. (C) GSE37745. CES means are labeled (blue) and marked (red diamond). Wilcoxon rank-sum tests between ADC and SCC indicate extremely significant *p*-values (labeled in red). LCC was excluded from comparison because of its excluding nature as an NSCLC subtype. The thick horizontal line indicates median. The top and the bottom of the box represent the upper and lower quartiles, respectively. The whiskers represent the upper and lower extremes, respectively. Dots indicate outlier data points.

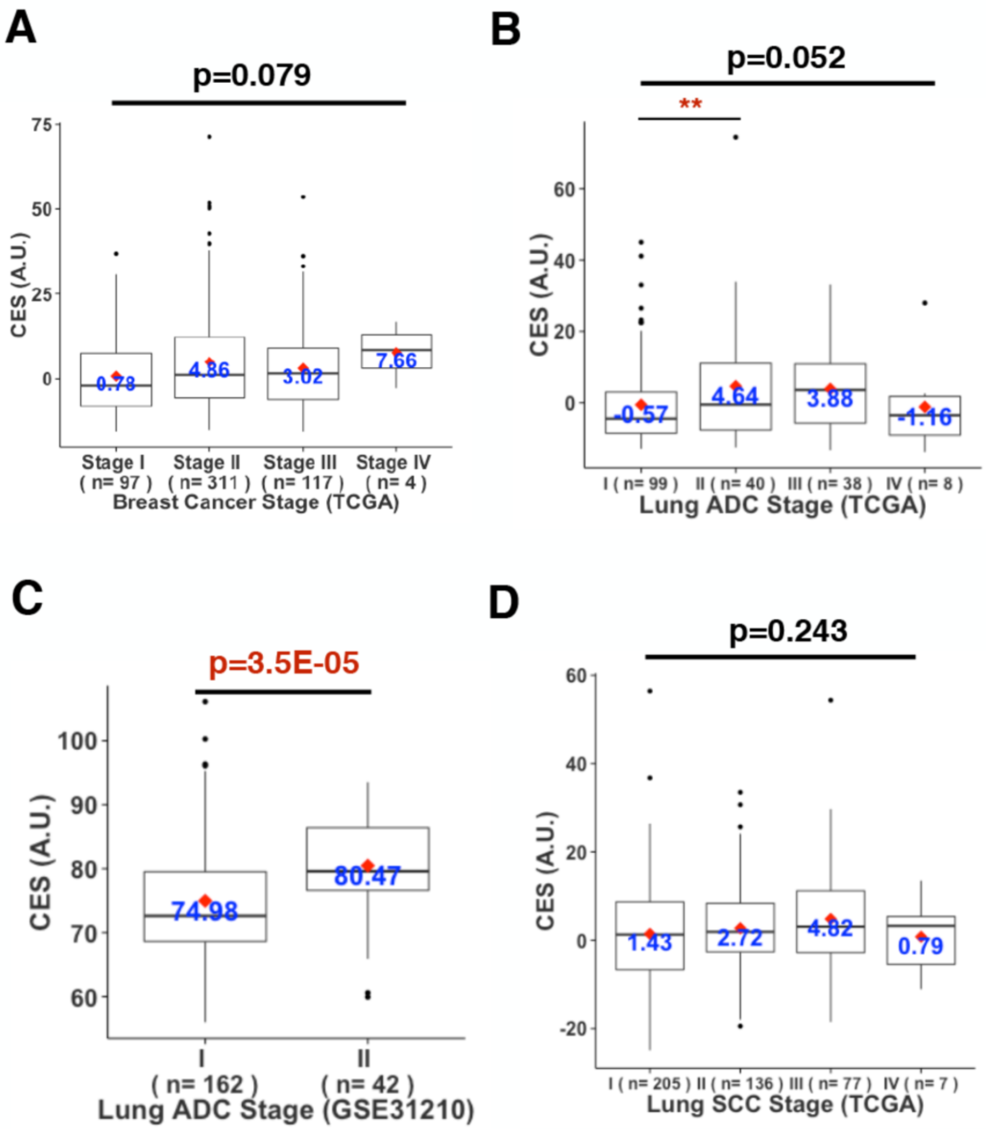

Supplementary Figure 8. Box plots showing correlation between CES and tumor stage in large and well-defined breast cancer and lung cancer datasets. (A) TCGA breast adenocarcinoma. (B) TCGA lung ADC. (C) GSE31210. (D) TCGA lung SCC. CES means are labeled (blue) and marked (red diamond). Kruskal-Wallis test and Wilcoxon rank-sum test were used for multi-group and two-group comparisons, respectively, and significant *p*-values are labeled in red. There is significant difference in CES between stage I and stage II lung ADCs. ** indicates highly significant *p*-value ($0.001 \leq p < 0.01$). The thick horizontal line indicates median. The top and the bottom of the box represent the upper and lower quartiles, respectively. The whiskers represent the upper and lower extremes, respectively. Dots indicate outlier data points.

A

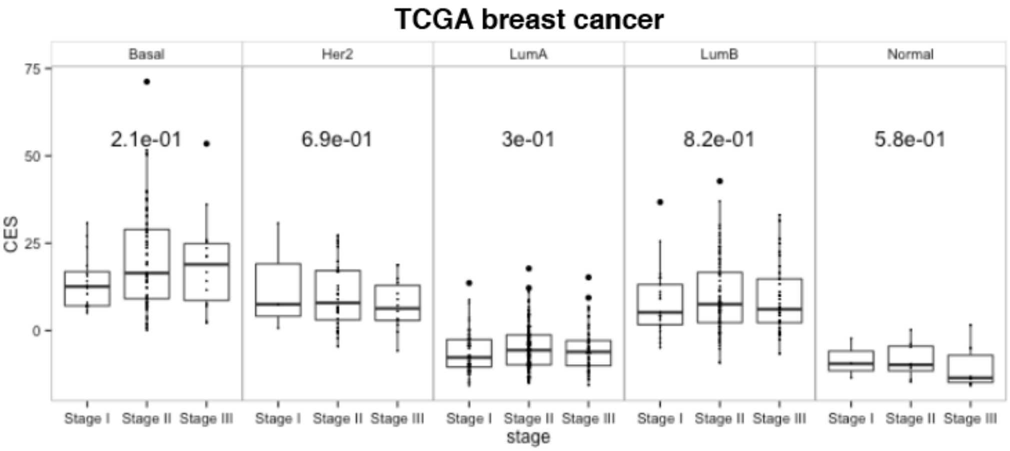

B

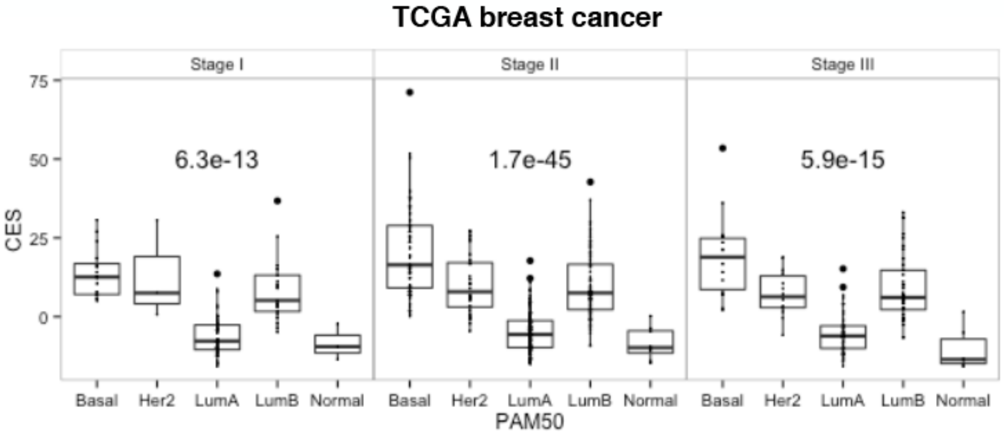

Supplementary Figure 9. **Box plots showing correlation between CES and stage or PAM50 subtype in TCGA breast cancer dataset.** (A) Difference of CES among stages upon patient stratification according to breast cancer PAM50 molecular subtype. (B) Difference of CES among PAM50 subtypes upon patient stratification according to tumor stage. *p*-values of Kruskal-Wallis tests are indicated in each graph. The thick horizontal line indicates median. The top and the bottom of the box represent the upper and lower quartiles, respectively. The whiskers represent the upper and lower extremes, respectively. Dots indicate outlier data points.

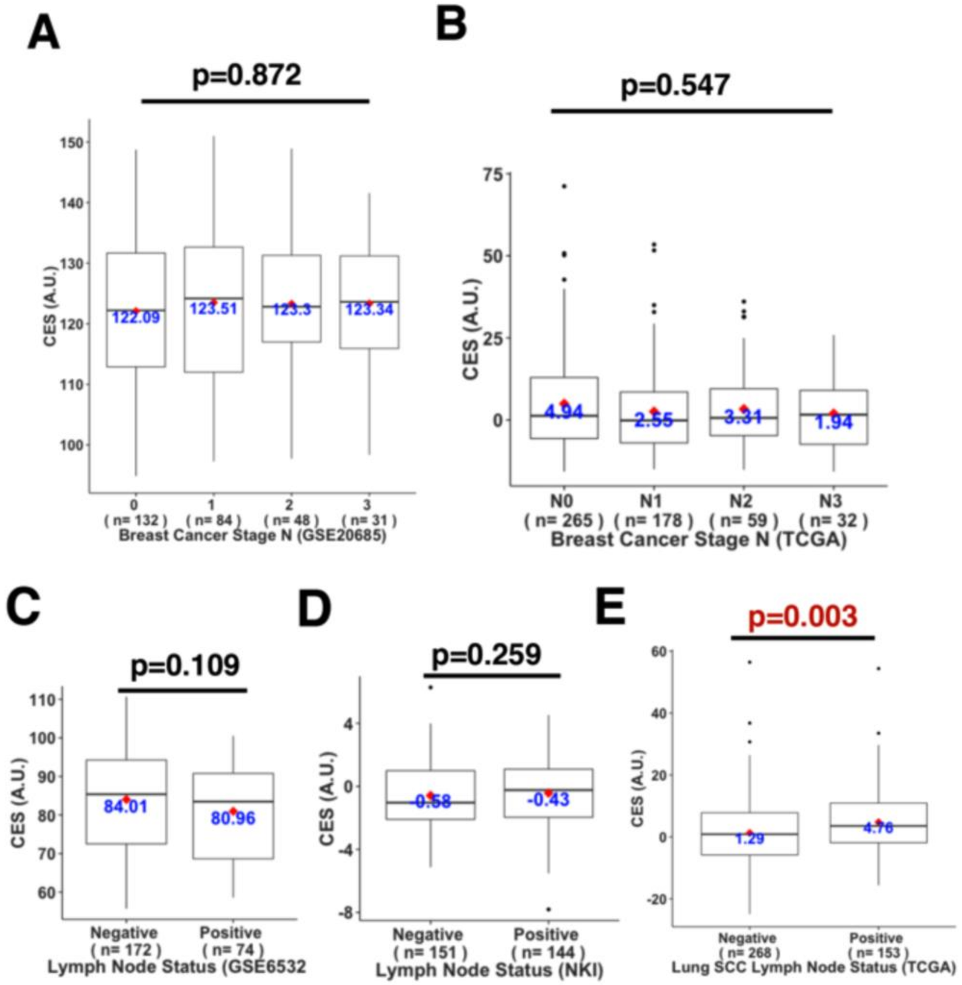

Supplementary Figure 10. **Box plots showing correlation between CES and lymph node status in a cancer type-specific manner.** (A) GSE20685. (B) TCGA breast adenocarcinoma. (C) GSE6532. (D) Breast cancer NKI. (E) TCGA lung SCC. CES is not significantly associated with lymph node invasion in tested breast cancer datasets, but is in TCGA lung SCC. Significant p-value is indicated in red. Mean CES values are labeled (blue) and marked (red diamond) in box plots. The thick horizontal line indicates median. The top and the bottom of the box represent the upper and lower quartiles, respectively. The whiskers represent the upper and lower extremes, respectively. Dots indicate outlier data points.

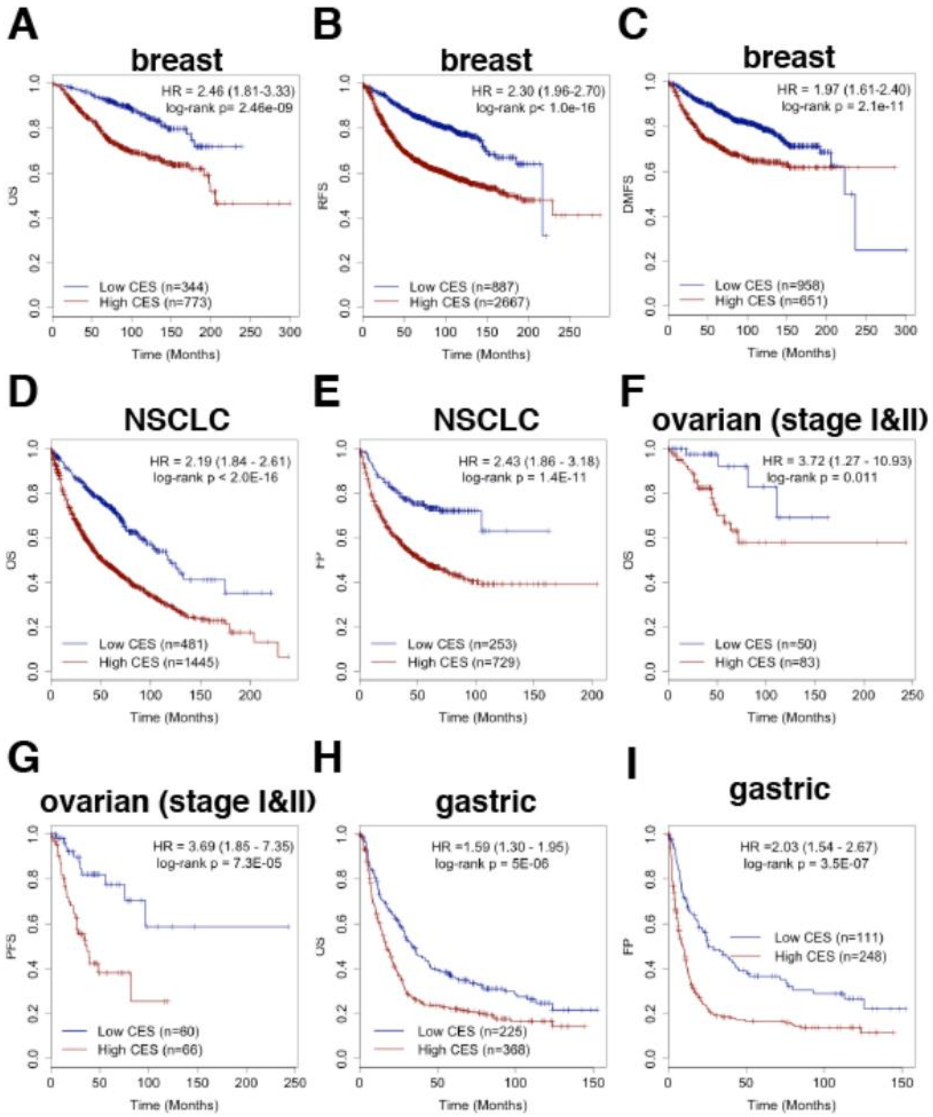

Supplementary Figure 11. **Prognostic impact of the CES signature across cancer types.** Kaplan-Meier plots of the prognostic impact of CES for (A) overall survival (OS), (B) relapse-free survival (RFS), (C) distant metastasis-free survival (DMFS) in breast cancer patients, for (D) OS and (E) first progression (FP) in NSCLC, (F) OS and (G) progression-free survival (PFS) for early-stage (stage I and II combined) ovarian patients, and (H) OS and (I) FP for gastric cancer patients using K-M Plotter.

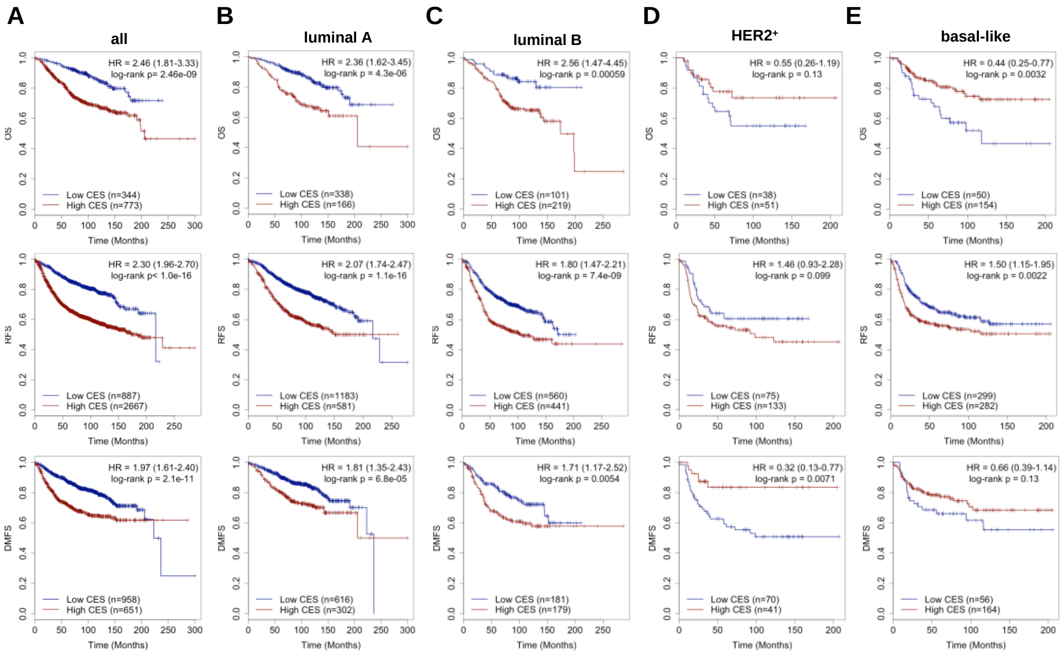

Supplementary Figure 12. Kaplan-Meier plots showing the prognostic impact of CES in breast cancer molecular subtypes. Patient overall survival (OS), relapse-free survival (RFS), and distant metastasis-free survival (DMFS) in (A) all patients, (B) luminal A, (C) luminal B, (D) HER2+, and (E) basal-like breast cancer subtypes by meta-analysis using K-M plotter database.

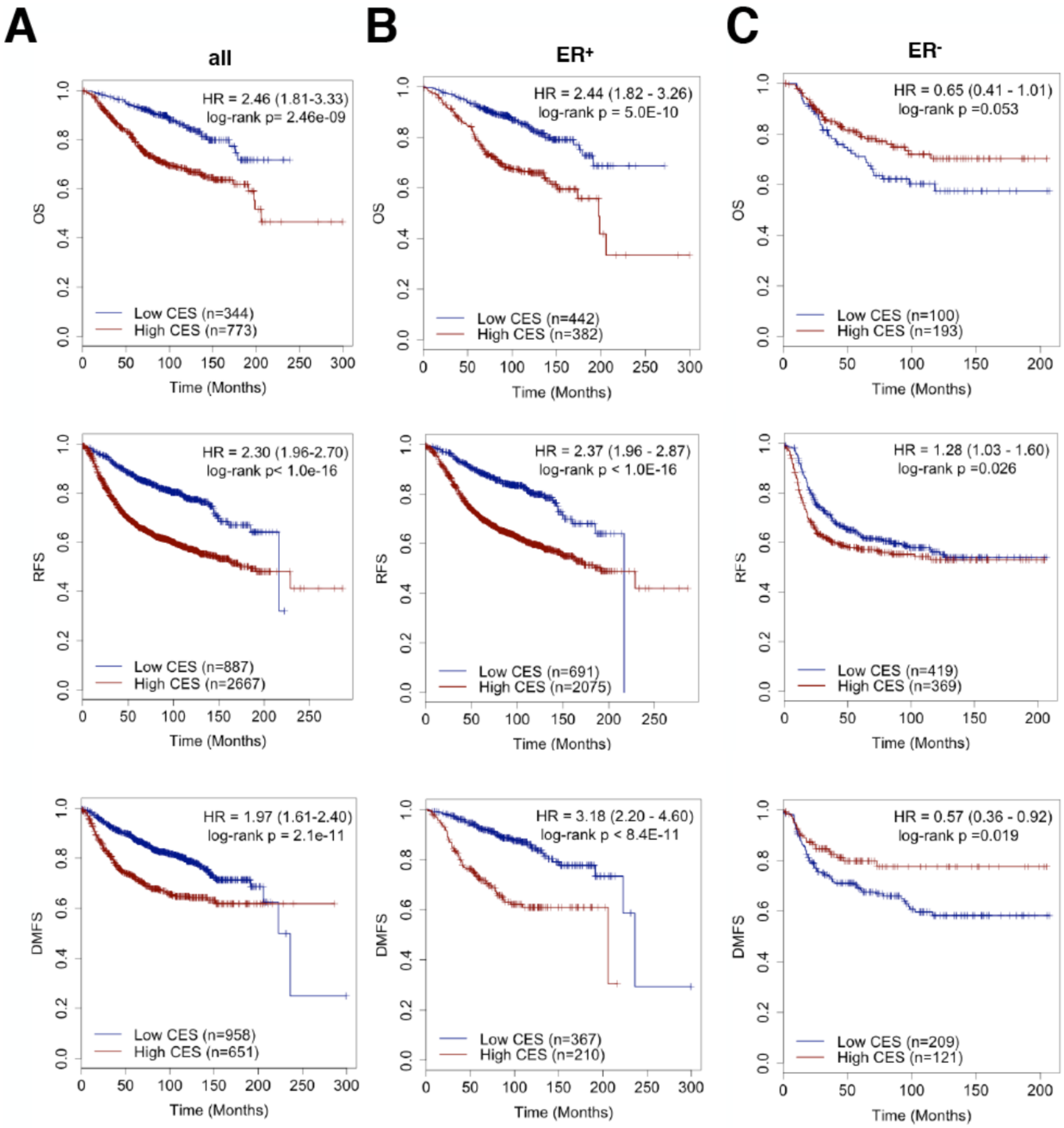

Supplementary Figure 13. Kaplan-Meier plots showing the prognostic impact of CES in breast cancer ER status. Patient overall survival (OS), relapse-free survival (RFS), and distant metastasis-free survival (DMFS) in (A) all breast cancer patients, (B) patients with ER+ tumors, and (C) patients with ER$^-$ tumors by meta-analysis using K-M plotter database.

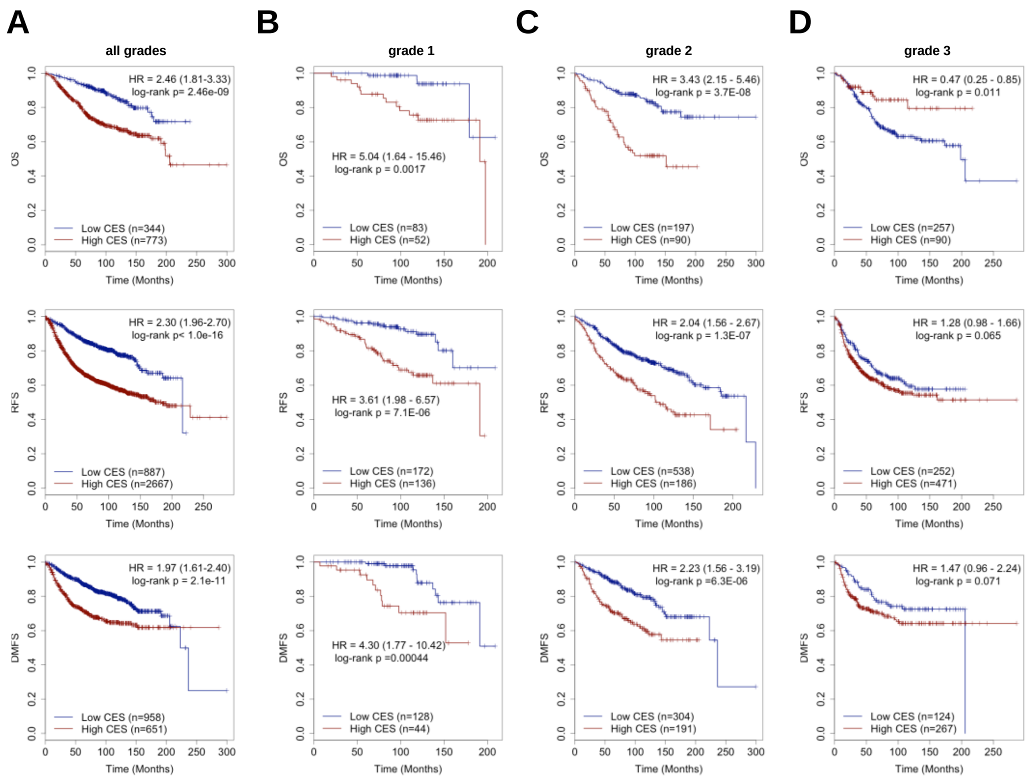

Supplementary Figure 14. Kaplan-Meier plots showing the prognostic impact of CES in breast cancer grades. Patient overall survival (OS), relapse-free survival (RFS), and distant metastasis-free survival (DMFS) for (A) all grades included, (B) grade 1, (C) grade 2, and (D) grade 3 by meta-analysis using K-M Plotter database.

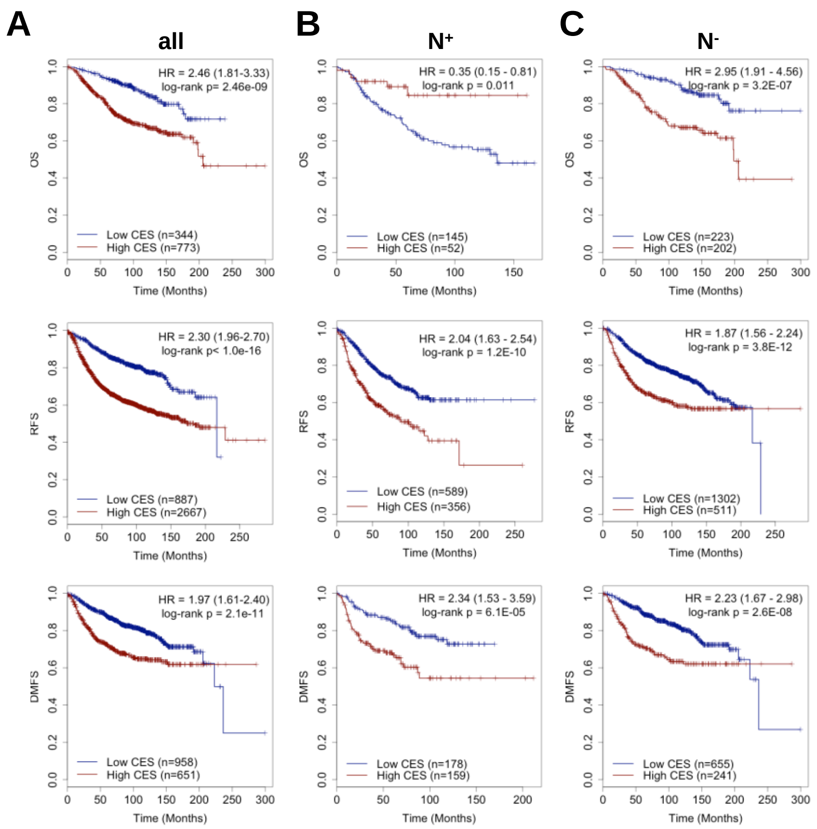

Supplementary Figure 15. Kaplan-Meier plots showing the prognostic impact of CES in breast cancer lymph node status. Patient overall survival (OS), relapse-free survival (RFS), and distant metastasis-free survival (DMFS) in (A) all patients, (B) lymph node positive (N+) patients, and (C) lymph node negative (N-) patients by meta-analysis using K-M Plotter database. Note that improved overall survival associated with high CES was likely caused by a biased distribution of higher grades (grade 2 and 3) in lymph node positive tumors for GSE3494 (p=0.0002, Fisher's exact test), of high grade (grade 3) in lymph node positive tumors for GSE20711 (p<0.001, Fisher's exact test), and of ER- status in lymph node positive (59/59) for GSE16446 that only included ER- tumors in the study, as well as that all lymph node positive patients in the 3 datasets were treated by systemic adjuvant therapies.

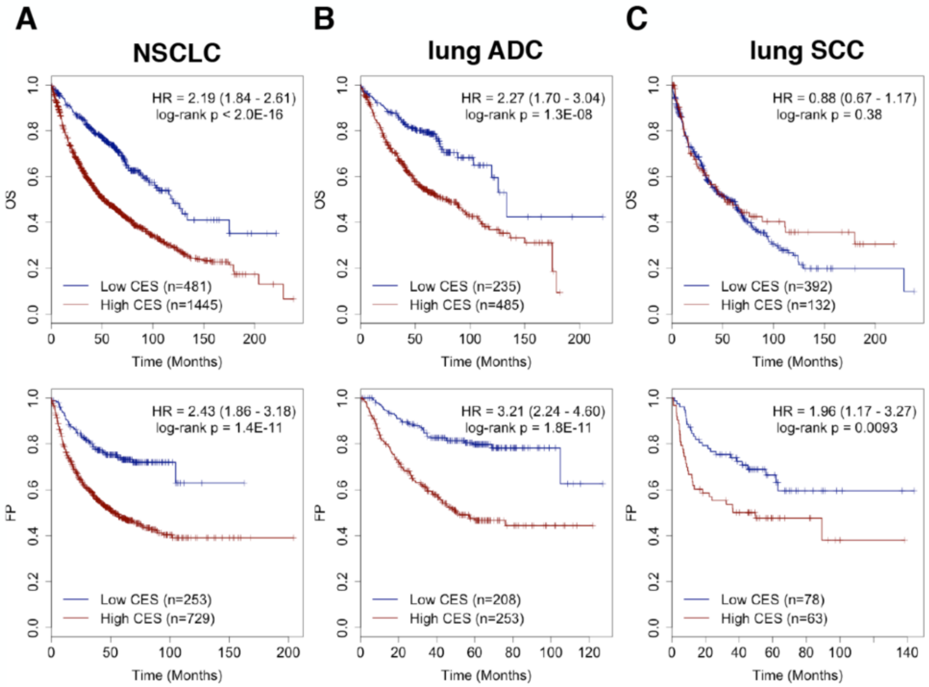

Supplementary Figure 16. Kaplan-Meier plots showing the prognostic impact of CES in NSCLC histological subtypes. Patient overall survival (OS) and first progression (FP) in (A) all NSCLC patients, (B) lung ADC patients, and (C) lung SCC patients by meta-analysis using K-M Plotter database.

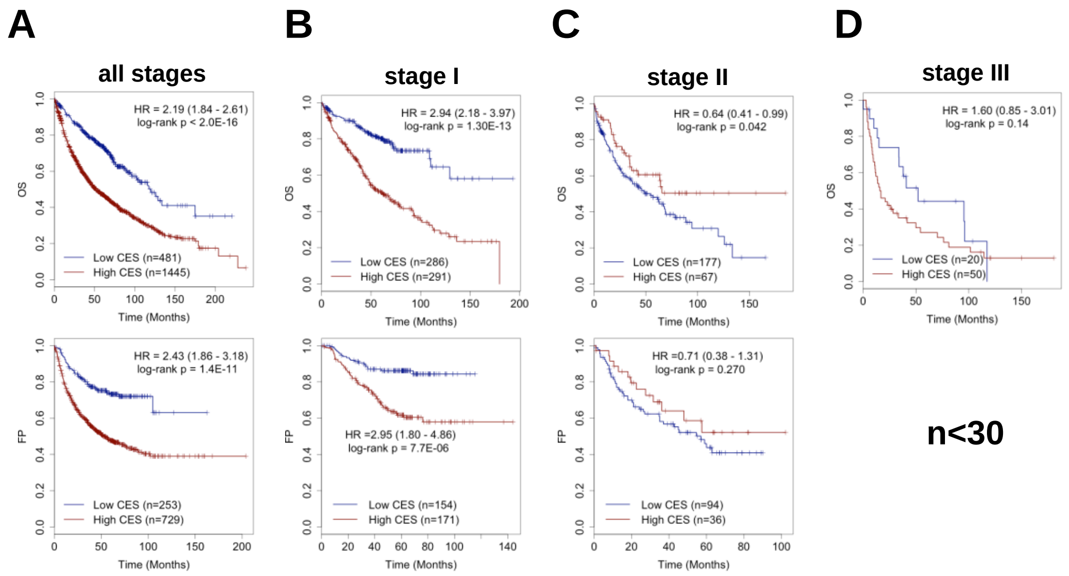

Supplementary Figure 17. Kaplan-Meier plots showing the prognostic impact of CES in NSCLC stages. Patient overall survival (OS) and first progression (FP) for (A) all stages included, (B) stage I, (C) stage II, and (D) stage III by meta-analysis using K-M Plotter database.

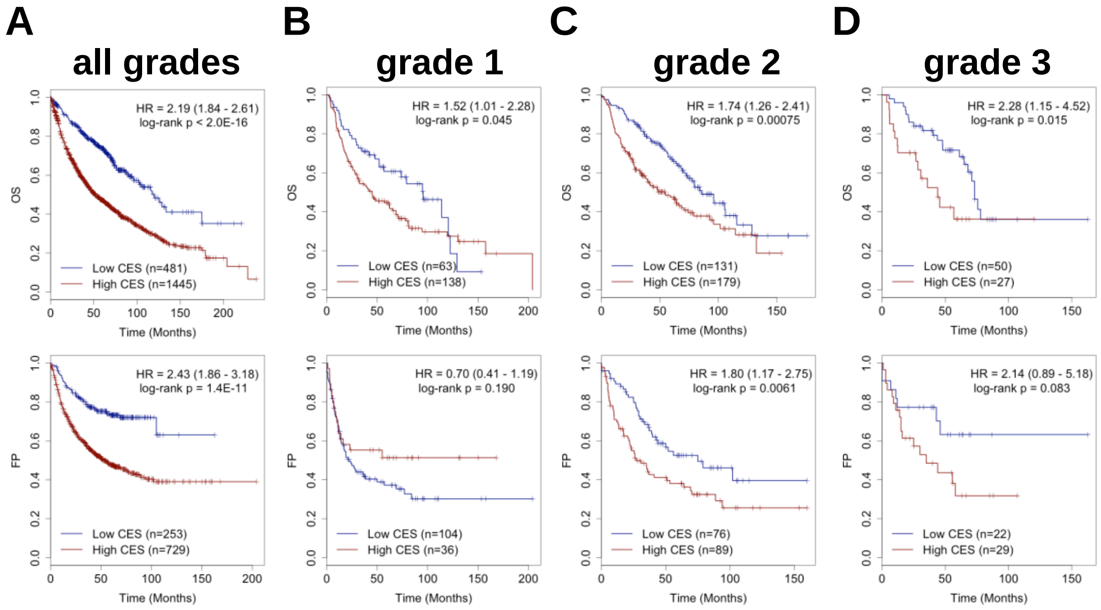

Supplementary Figure 18. Kaplan-Meier plots showing the prognostic impact of CES in NSCLC grades. Patient overall survival (OS) and first progression (FP) for (A) all grades included, (B) grade 1, (C) grade 2, and (D) grade 3 by meta-analysis using K-M Plotter database.

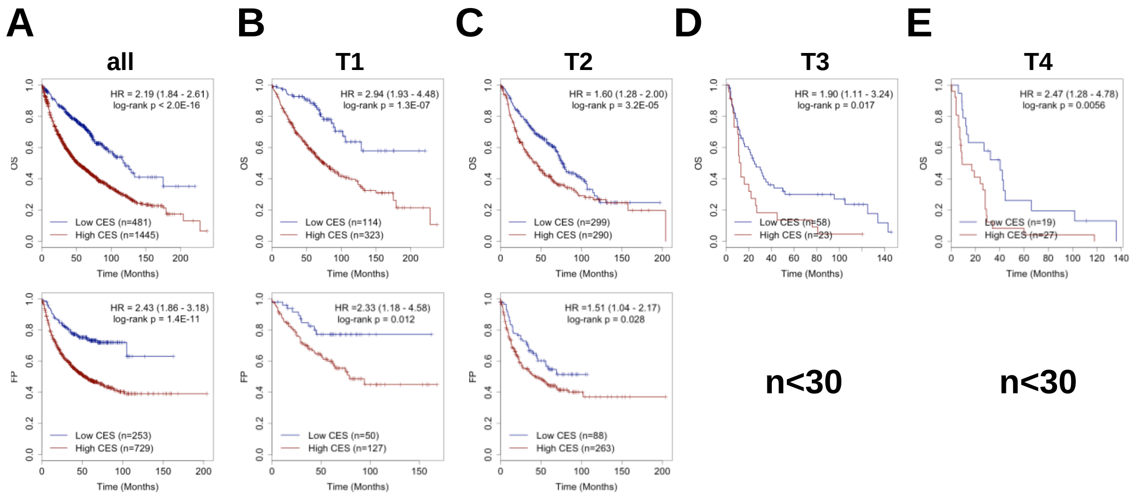

Supplementary Figure 19. Kaplan-Meier plots showing the prognostic impact of CES in NSCLC staging factor T classes. Patient overall survival (OS) and first progression (FP) for (A) all patients included, (B) staging factor T1, (C) staging factor T2, (D) staging factor T3 and (E) staging factor T4 by meta-analysis using K-M Plotter database.

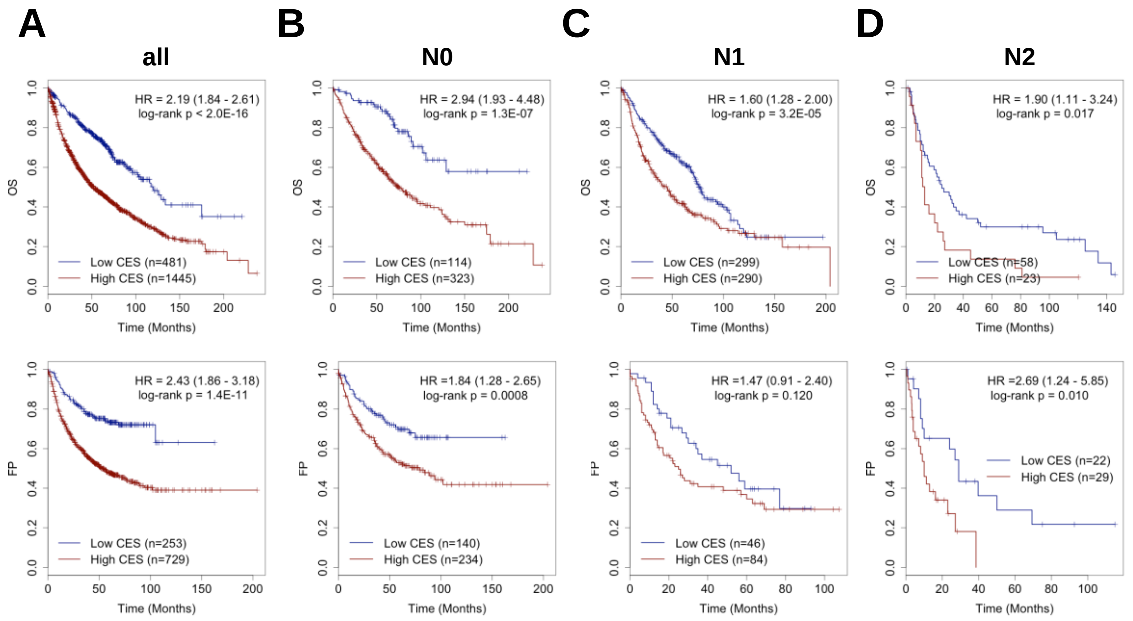

Supplementary Figure 20. Kaplan-Meier plots of the prognostic impact of CES in NSCLC staging factor N classes. Patient overall survival (OS) and first progression (FP) for (A) all patients included, (B) staging factor N0, (C) staging factor N1, and (D) staging factor N2 by meta-analysis using K-M plotter database.

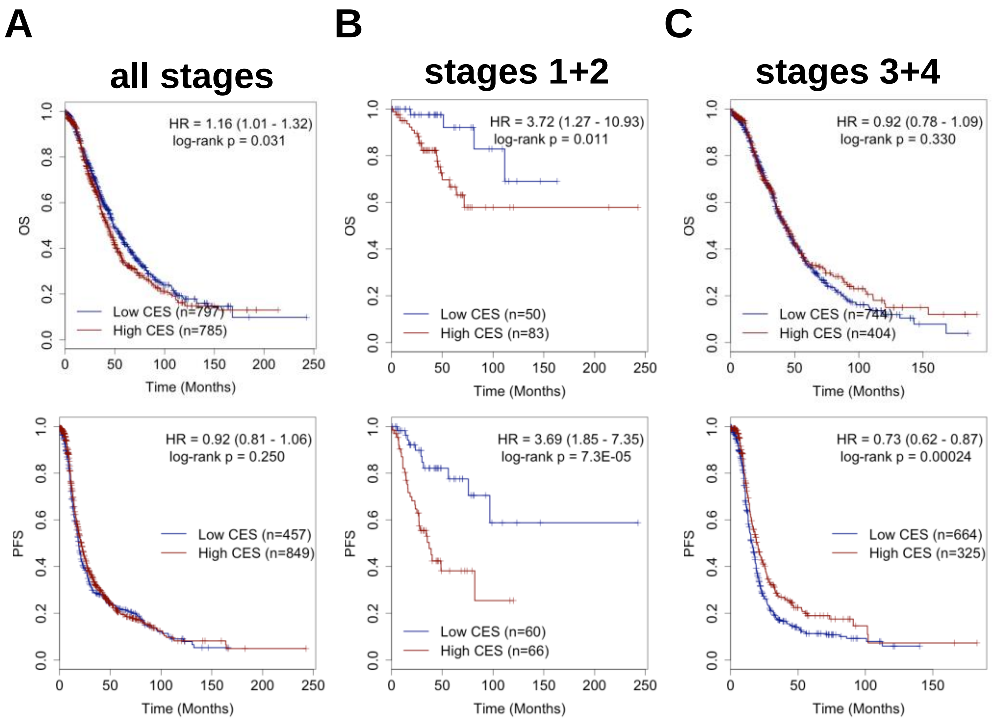

Supplementary Figure 21. Kaplan-Meier plots showing the prognostic impact of CES in ovarian cancer stages. Ovarian cancer patient overall survival (OS) and progression-free survival (PFS) for (A) all stages included, (B) early stage patients only (stages I and 2 combined), and (C) late stage patients only (stages 3 and 4 combined) by meta-analysis using K-M Plotter database.

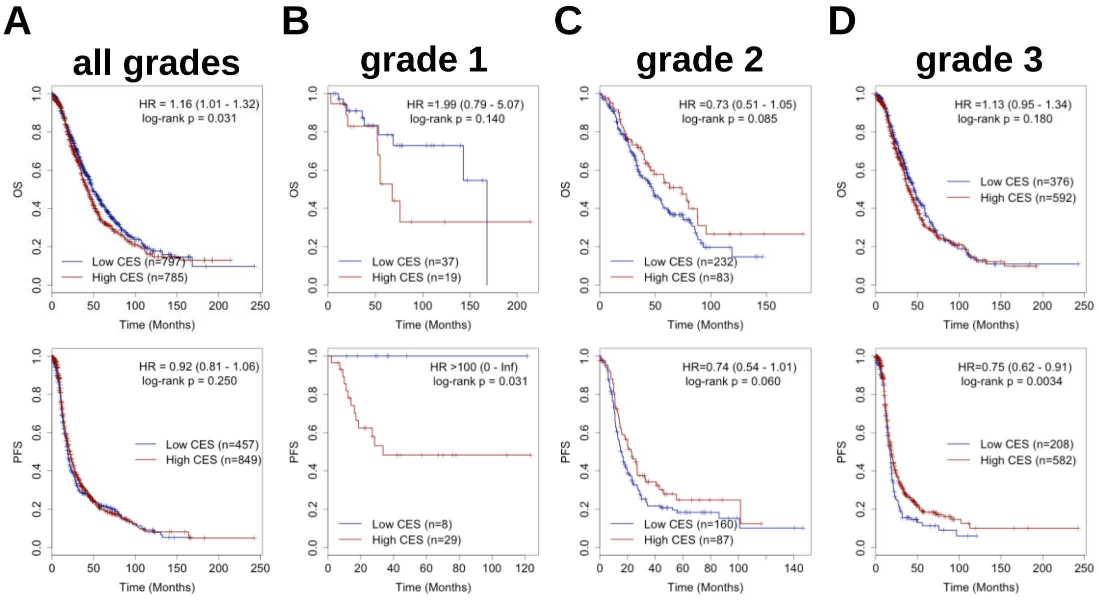

Supplementary Figure 22. Kaplan-Meier plots showing the prognostic impact of CES in ovarian cancer grades. Patient overall survival (OS) and progression-free survival (PFS) for (A) all grades included, (B) grade 1, (C) grade 2, and (D) grade 3 by meta-analysis using K-M Plotter database.

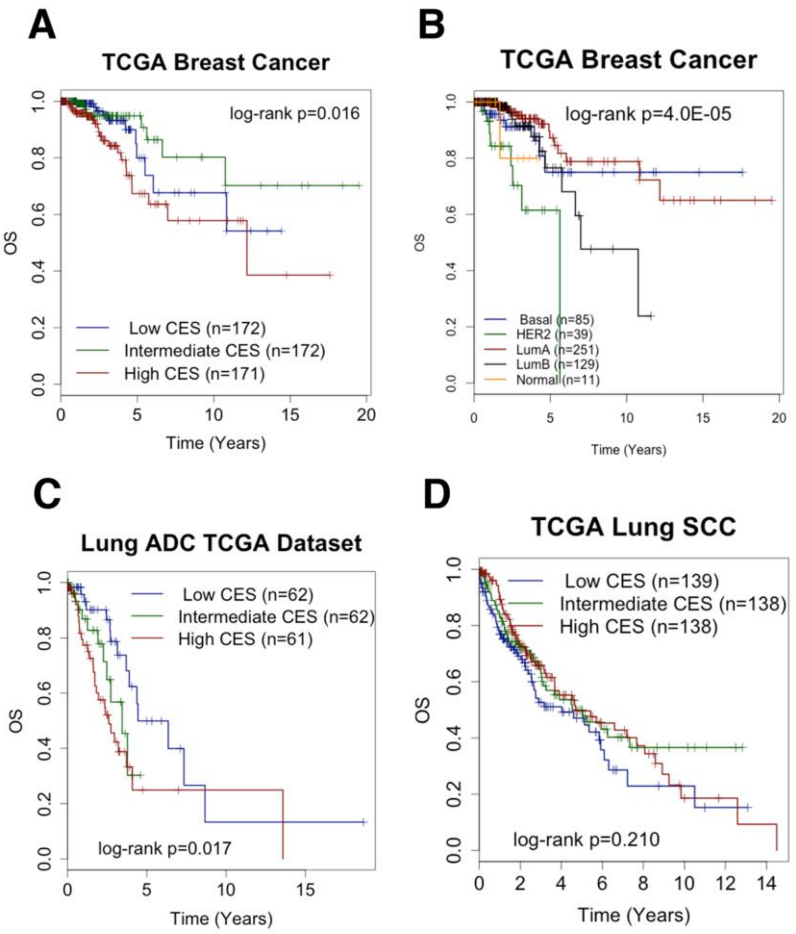

Supplementary Figure 23. Kaplan-Meier survival analysis on TCGA breast adenocarcinoma, lung ADC and lung SCC datasets. (A) TCGA breast cancer overall survival estimation for CES tertiles. (B) TCGA breast cancer overall survival estimation for PAM50 molecular subtypes. (C) TCGA lung ADC overall survival estimation for CES tertiles. (D) TCGA lung SCC overall survival estimation for CES tertiles.

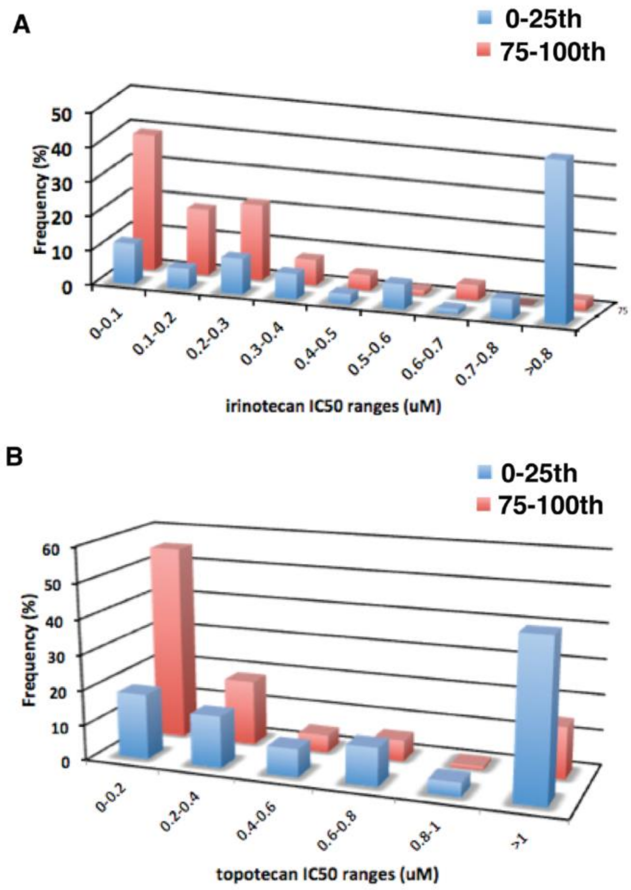

Supplementary Figure 24. Percentage frequency bar graphs showing inverse correlations between CES values and drug IC_{50}s. (A) irinotecan. (B) topotecan. Drug IC_{50}s for CCLE cell lines in the top (75-100th, red) and bottom CES (0-25th, blue) quartiles are shown.

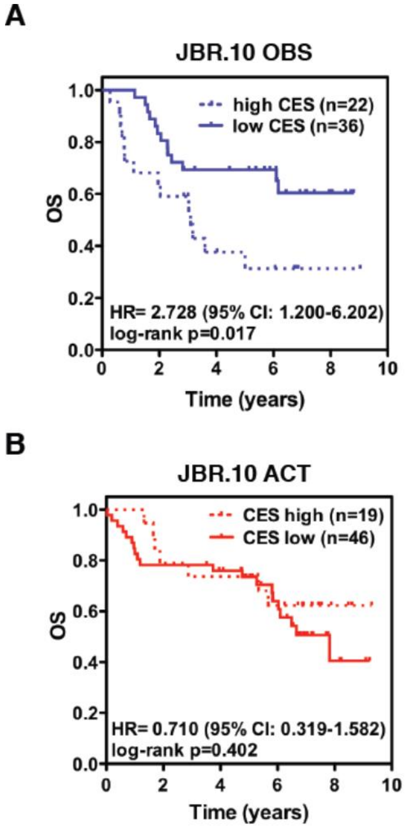

Supplementary Figure 25. Kaplan-Meier survival plots showing that ACT specifically benefits overall survival for patients with high CES early stage (I and II combined) NSCLC in the JBR.10 trial. Hazard ratio, 95% CI and log-rank *p*-values are indicated. (A) High CES prognosticates poor OS for patients without adjuvant chemotherapy (OBS). (B) For patients with adjuvant chemotherapy (ACT), there is no significant difference in OS between high CES and low CES patient groups.

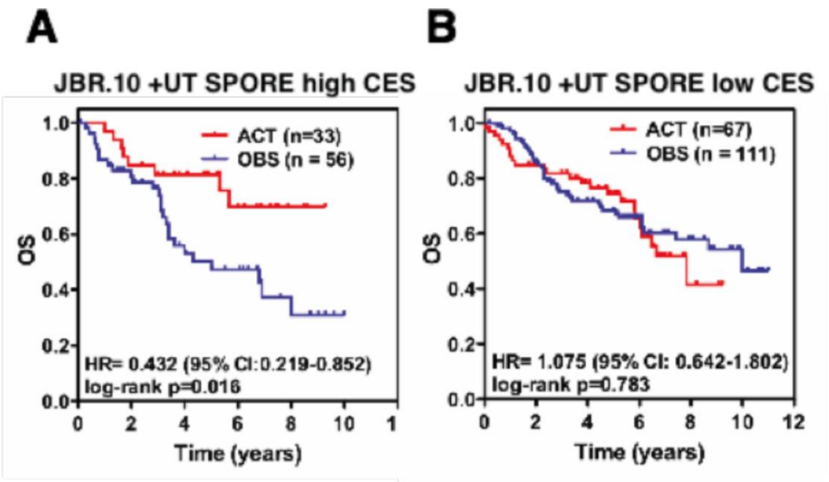

Supplementary Figure 26. Kaplan-Meier survival analysis of the CES on combined JBR.10 and UT SPORE early stage NSCLC patients. (A) High CES group (top tertile) shows a significant effect of adjuvant chemotherapy (ACT) on patient survival compared to no ACT (OBS). (B) ACT did not improve overall survival for the CES low group (lower two tertiles) compared to OBS.

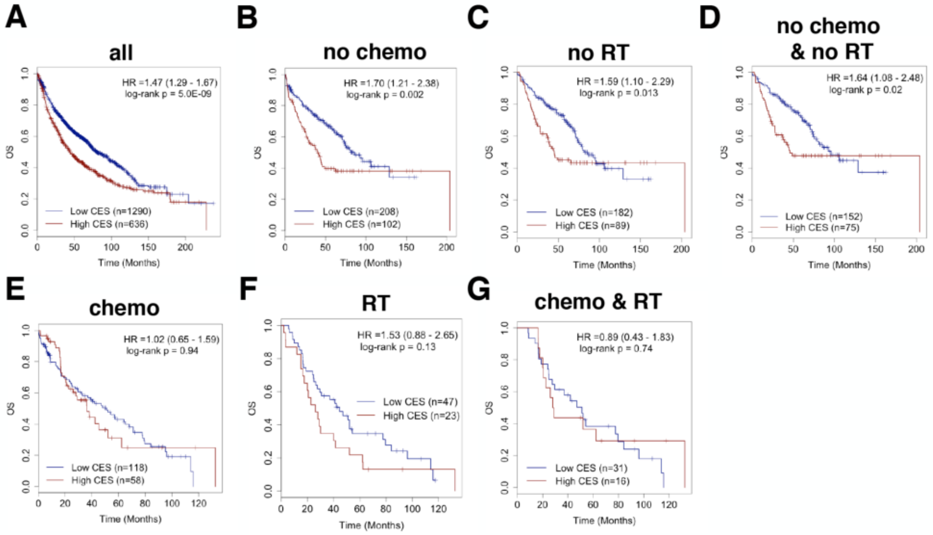

Supplementary Figure 27. Kaplan-Meier survival analysis of CES on NSCLC patient overall survival after different treatments. Patient overall survival (OS) for (A) all patients included, (B) with no adjuvant chemotherapy, (C) with no adjuvant radiotherapy (RT), (D) with no chemotherapy or RT, (E) with chemotherapy, (F) with RT, and (G) with both chemotherapy and RT by meta-analysis using K-M Plotter database.

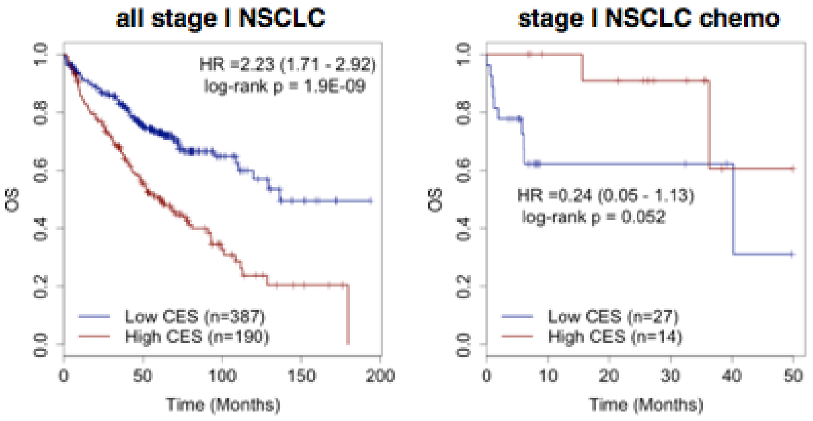

Supplementary Figure 28. Kaplan-Meier survival analysis of CES on stage I NSCLC patients with or without adjuvant chemotherapy. Patient overall survival (OS) for (left) all stage I NSCLC patients included and (right) patients treated with adjuvant chemotherapy by meta-analysis using K-M Plotter database.

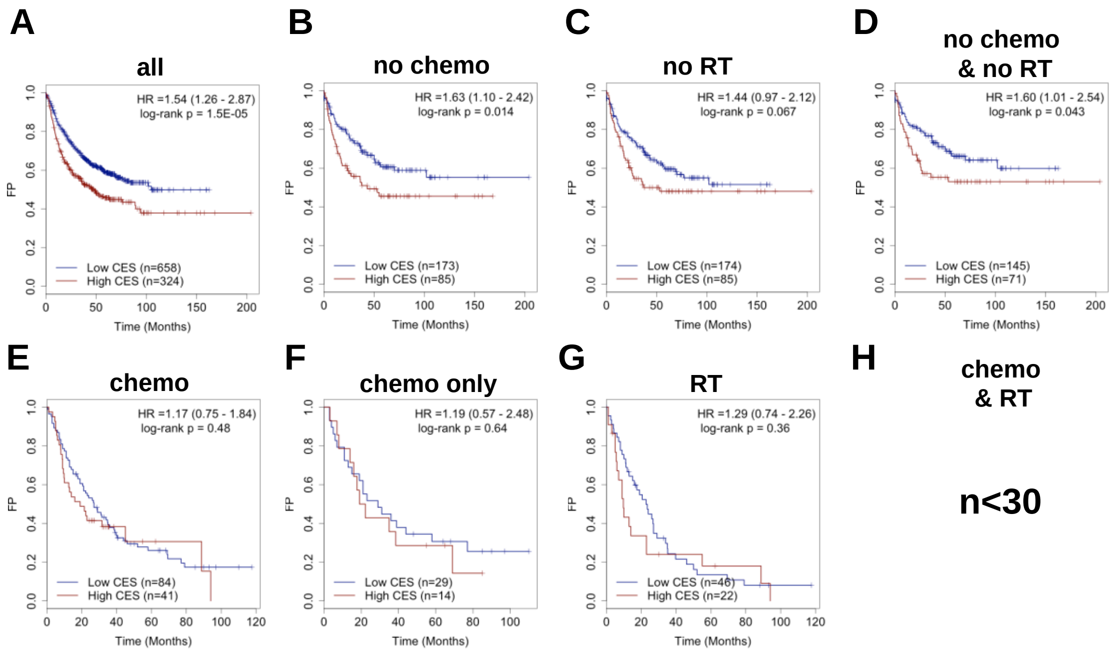

Supplementary Figure 29. Kaplan-Meier survival analysis of CES on NSCLC patient first progression after different treatments. Patient first progression (FP) for (A) all NSCLC patients included, (B) with no adjuvant chemotherapy, (C) with no adjuvant radiotherapy (no RT), (D) with no chemotherapy or RT, (E) with chemotherapy, (F) with chemotherapy only, (G) with RT by meta-analysis using K-M Plotter database. (H) Sample size for patients upon both chemotherapy and RT is excluded from analysis due to small size (n<30).

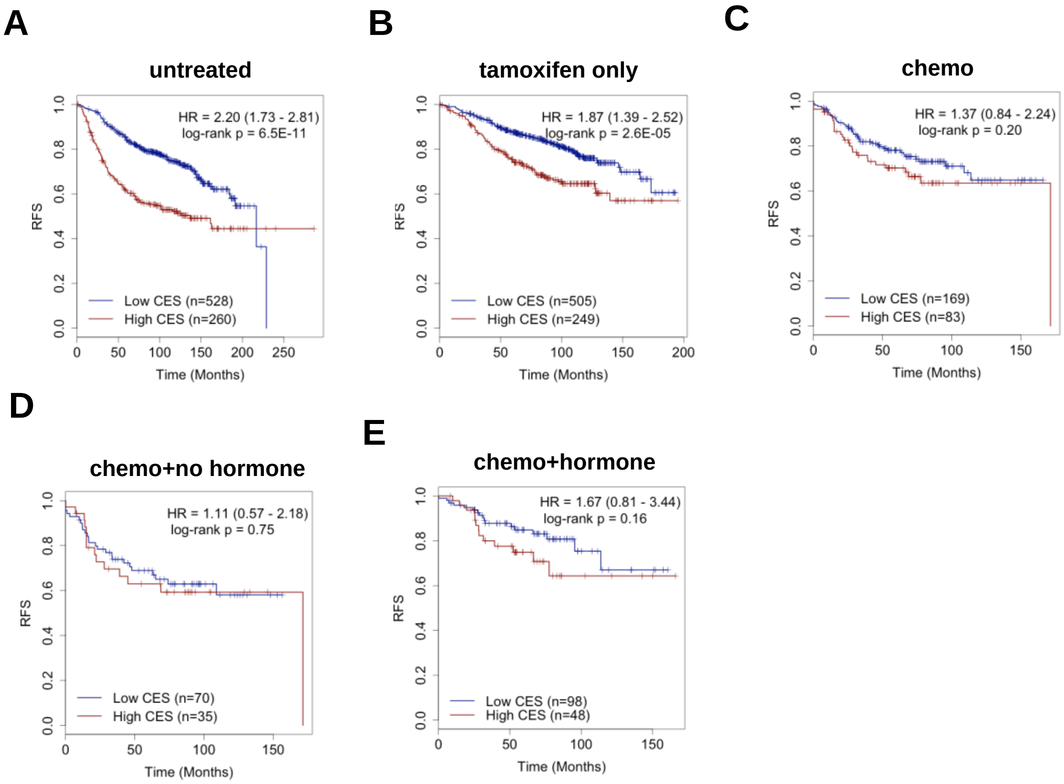

Supplementary Figure 30. Kaplan-Meier survival analysis of CES on ER+ breast cancer patient relapse-free survival upon different treatments. ER+ breast cancer patient relapse-free survival (RFS) for (A) untreated patients, (B) tamoxifen only, (C) adjuvant chemotherapy, (D) with adjuvant chemotherapy but without hormone therapy, and (E) with both chemotherapy and hormone therapy, by meta-analysis using K-M Plotter database.

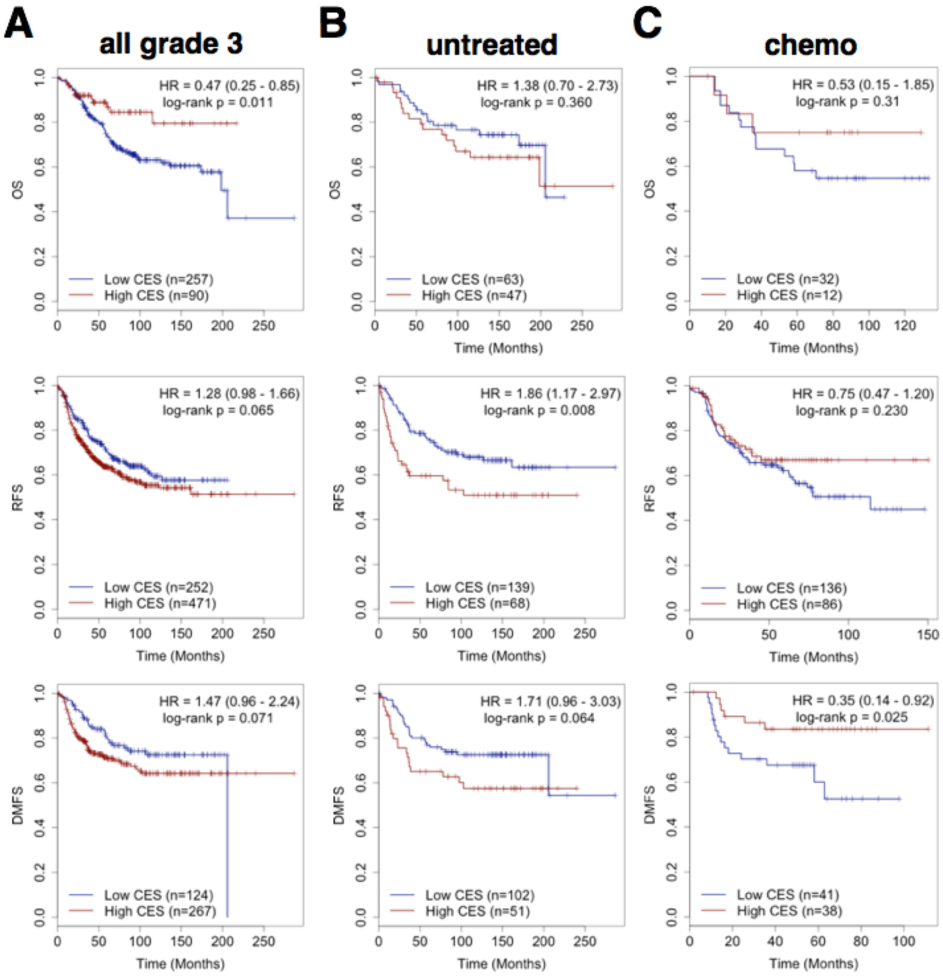

Supplementary Figure 31. Kaplan-Meier survival analysis on CES for high grade breast cancer patients. Patient overall survival (OS), relapse-free survival (RFS) and distant metastasis-free survival (DMFS) for (A) all high grade (grade 3) patients, (B) untreated high grade patients, and (C) high grade patients after adjuvant chemotherapy by meta-analysis using K-M Plotter database.

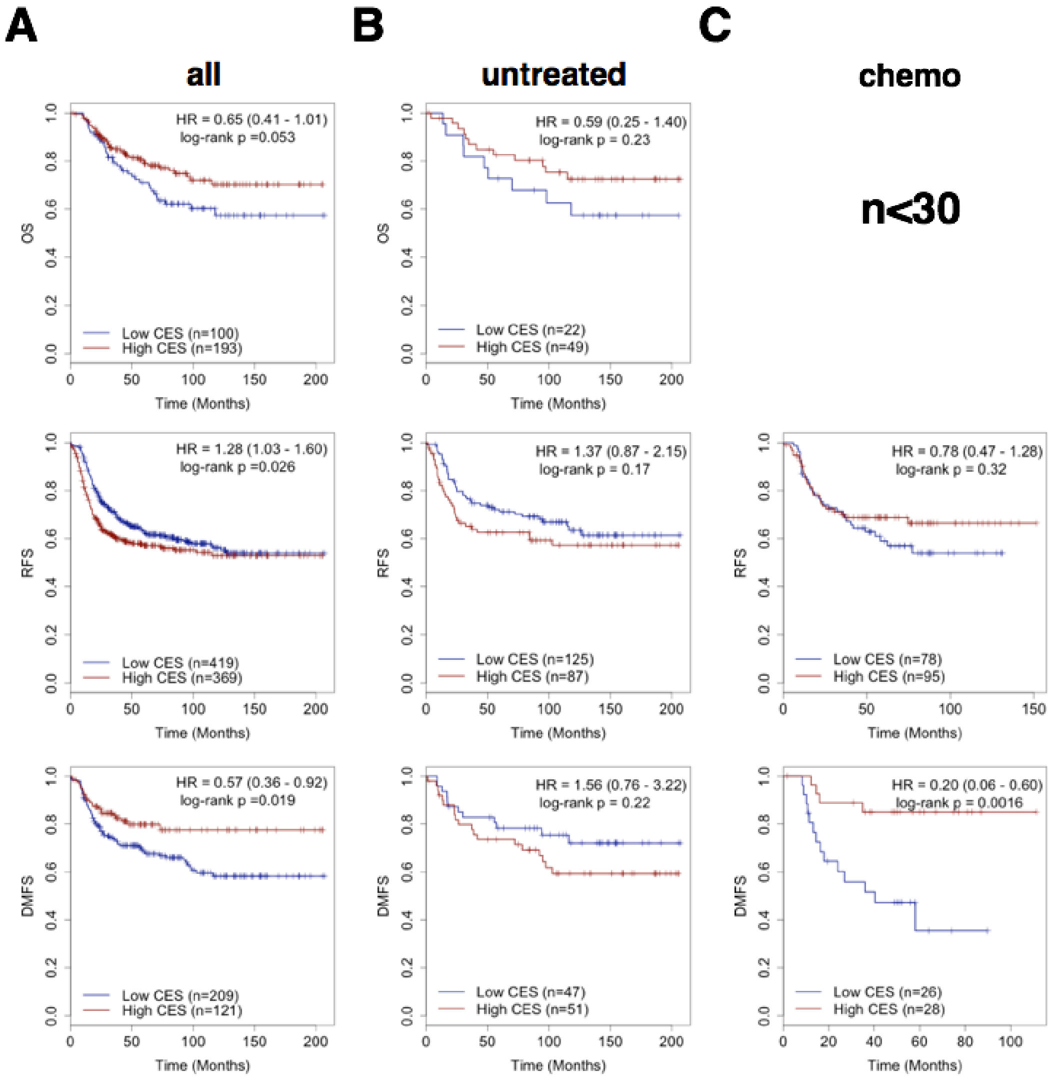

Supplementary Figure 32. Kaplan-Meier survival analysis of CES on ER- breast cancer patient survival with or without adjuvant adjuvant chemotherapy. Patient overall survival (OS), relapse-free survival (RFS) and distant metastasis-free survival (DMFS) for (A) all ER- patients (all), (B) untreated ER- patients (untreated), and (C) ER- patients after adjuvant chemotherapy (chemo) by meta-analysis using K-M Plotter database.

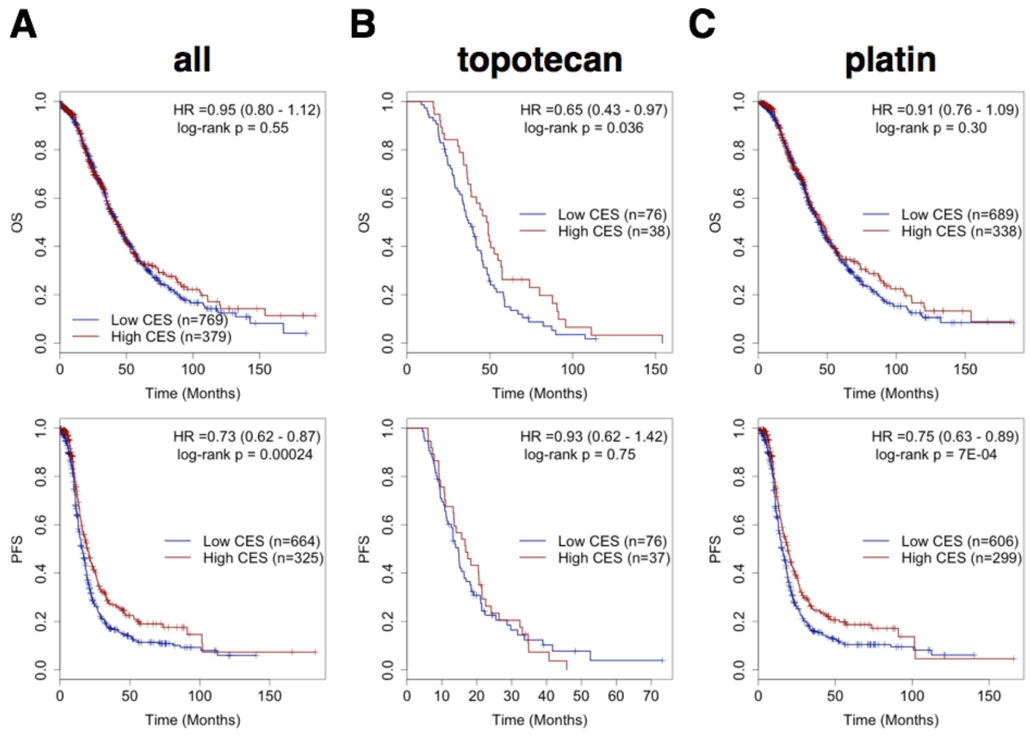

Supplementary Figure 33. Kaplan-Meier plots on CES in late stage ovarian cancer patient survival after different adjuvant chemotherapies. Patient (upper panels) overall survival (OS) and (lower panels) progression-free survival (PFS) for (A) all late stage ovarian cancer patients (stages III and IV combined), (B) late stage patients treated with topotecan, and (C) late stage patients treated with platin by meta-analysis using K-M Plotter database.

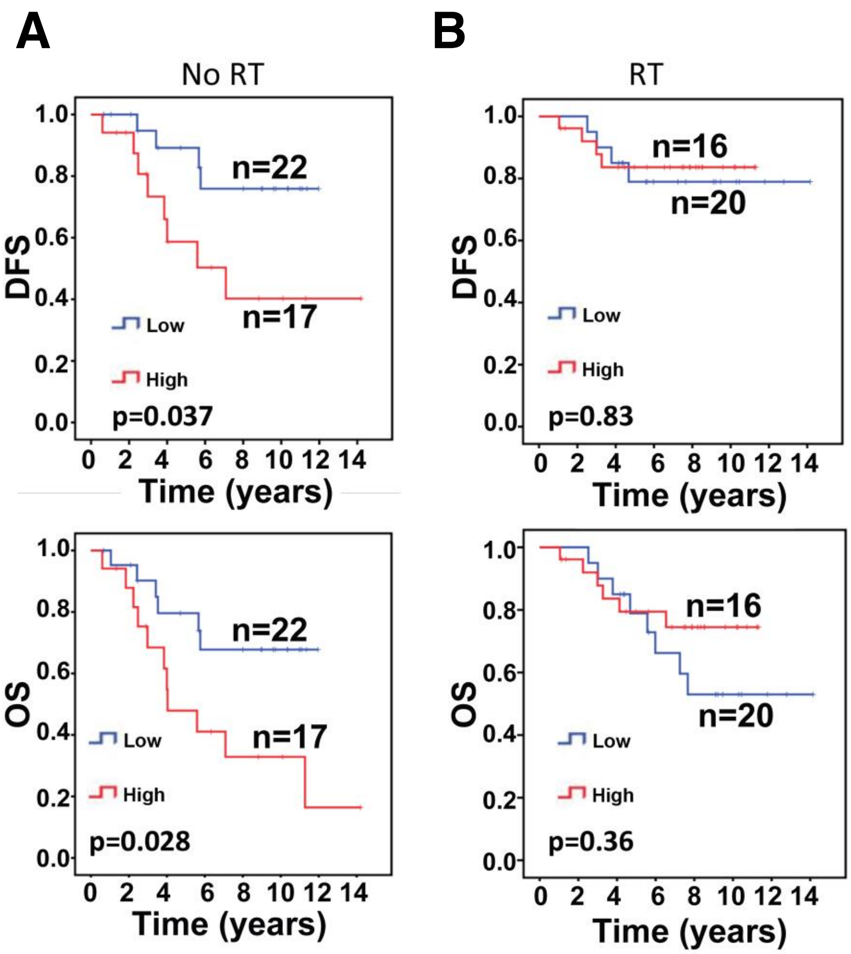

Supplementary Figure 34. Kaplan-Meier survival plots on the CES for breast cancer patient survival in the Gray dataset. Patient (upper panels) disease-free survival (DFS) and (lower panels) overall survival (OS) for patients (A) without adjuvant radiotherapy (RT) and (B) with RT are evaluated. CES values are indicated for high (red) and low (blue. (A) High CES values associate with poor DFS and OS, respectively (p<0.05) with no RT. (B) Upon RT, patients with high CES values show hazard ratios that are not significantly different from those with low CES values for both DFS and OS (p>0.05).

Supplementary Tables:

Supplementary Table 1. Sample characteristics of GEO datasets for differential expression of CEN/KT genes in human cancers.

Tissue Origin	Breast	Breast	Breast	Lung	Ovarian	Liver
**Dataset**	**GSE21422 (n=19)**	**GSE10780 (n=185)**	**GSE3744 (n=47)**	**GSE19188 (n=156)**	**GSE14407 (n=24)**	**GSE6764 (n=75)**
**Gender**						
Female	19	185	47	34	24	-
Male	-	-	-	100	-	-
Unknown	-	-	14407	22	-	75
**Sample Characteristics**	5 (healthy)<br>5 (DCIS)<br>9 (IDC)	143 (normal)<br>42 (IDC)	7 (healthy)<br>18 (basal-like)<br>20 (non-basal-like)<br>2 (Brca1$^-$)	65 (helathy)<br>91 (tumors)	12 (healthy)<br>12 (ADC)	10 (healthy)<br>13 (cirrhotic)<br>10 (low-grade dysplastia)<br>7 (high-grade dysplasia)<br>35 HCC
			MBR grade: 1 (I)<br>4 (II)<br>35 (III)	*Tumor Subtype*: 45 (ADC)<br>27 (SCC)<br>19 (LCC)	*Tumor Stage*: 2 (I)<br>2 (II)<br>6 (III)<br>2 (IV)	*HCC Classification*: 8 (very early)<br>10 (early)<br>7 (advanced)<br>10 (very advanced)<br>2 (unknown)
				Tumor Stage: 51 (I)<br>20 (II)<br>8 (IIIA)<br>3 (IV)<br>9 (Unknown)	*Tumor Grade*: 2 (grade 2)<br>10 (grade 3)	*HCC Tumor Size*: 10 (0.8-2cm)<br>13 (2-5cm)<br>8 (>5cm)<br>2 (Diffuse)
						HCC differentiation: 12 (well)<br>9 (moderate)<br>12 (poor)
						HCC vasc.iInvasion: 15 (No)<br>11 (micro)<br>7 (macro)
**Platform**<br>**Normalization Method**	HG-U133PLUS2<br>GCRMA	HG-U133PLUS2<br>RMA	HG-U133PLUS2<br>GCRMA	HG-U133PLUS2<br>RMA	HG-U133PLUS2<br>MAS5	HG-U133PLUS2<br>GCRMA
**Citation (PMID)**	(Kretschmer et al., 2011)<br>PMID: 21314937	(Chen et al., 2010)<br>PMID: 19266279	(Richardson et al., 2006)<br>PMID: 16473279	(Hou et al., 2010)<br>PMID: 20421987	Bowen et al. (2009)<br>PMID: 20040092	Wurmbach et al. (2007)<br>PMID: 17393520

Tissue Origin	Pancreatic	Colon	Nasopharyngeal	Gastric	Cervical	Head and Neck	Prostate	Brain
**Dataset**	**GSE16515 (n=52)**	**GSE8671 (n=64)**	**GSE12452 (n=41)**	**GSE13911 (n=69)**	**GSE6791 (n=28)**	**GSE6791 (n=56)**	**GSE3325 (n=19)**	**GSE4290 (n=180)**
**Gender**								
Female	18	8	-	23	20	20	-	-
Male	34	20	-	15	-	34	19	-
Unknown	-	4	41	-	-	-	-	180
**Sample Characteristics**	16 (healthy)<br>36 (cancer)	32 (matched normal)<br>32 (adenoma)	10 (normal)<br>31 (carcinoma)	31 (normal)<br>38 (tumor)	8 (normal)<br>20 (cancer)	14 (normal)<br>40 (cancer)	6 (benign)<br>7 (primary malignant)<br>6 (metastatic)	23 (epilepsy)<br>157 (tumor)
		Tumor Size: 6 (1.0 cm)<br>10 (1.5 cm)<br>7 (2.0 cm)<br>6 (3.0 cm)<br>1 (3.5 cm)<br>2 (4.0 cm)	*Tumor Stage*: 6 (T1N0)<br>10 (T1N1)<br>5 (T2N1)<br>6 (T2N2)<br>2 (T3N2)<br>2 (other)	*MSI Status*: 19 (MSI)<br>20 (MSS)	*Stage*: 16 (IB)<br>3 (II/III)<br>1 (IV)	*Stage*: 6 (I/II)<br>8 (III)<br>10 (IV)<br>16 (Unknown)		*tumor type*: 26 (astrocytoma): 7 (grade 2), 19 (garde 3)<br>77 (glioblastoma): 77 (grade 4)<br>50 (oligodendroglioma): 38 (grade 2), 12 (grade 3)<br>4 (other): Unknown
		Degree: 10 (H)<br>3 (H/L)<br>15 (L)	*WHO histology*: 16 (IIA)<br>15 (IIB)	*Lauren*: 6 (D)<br>16 (I)<br>4 (M)<br>2 (U)	*Grade*: 8 (well/mod.)<br>12 (poor/undiff.)	*Grade*: 28 (well/mod.)<br>12 (Unknown)		
				Tumor Stage: 1 (T1N0Mx)<br>2 (T2N0Mx)<br>7 (T2N1Mx)<br>1 (T2N2Mx)<br>1 (T2N3Mx)<br>6 (T3N0Mx)<br>14 (T3N1Mx)<br>1 (T3N2M1)<br>2 (T3N2Mx)<br>3 (T3N3Mx)				
**Platform**<br>**Normalization Method**	HG-U133PLUS2<br>GCRMA	HG-U133PLUS2<br>RMA	HG-U133PLUS2<br>RMA	HG-U133PLUS2<br>RMA	HG-U133PLUS2<br>RMA	HG-U133PLUS2<br>RMA	HG-U133PLUS2<br>RMA	HG-U133PLUS2<br>Li-Wong
**Citation (PMID)**	Pei et al. (2009)<br>PMID: 19732725	Sabates-Bellver et al. (2007)<br>PMID: 18171984	Sengupta et al. (2006)<br>PMID: 16912175	D'Errico et al. (2009)<br>PMID: 19081245	Pyeon et al. (2007)<br>PMID: 17510386	Pyeon et al. (2007)<br>PMID: 17510386	Varambally et al. (2005)<br>PMID: 16286247	Sun et al. (2006)<br>PMID: 16616334

The GEO datasets are used in Supplementary Fig. 1 and Supplementary Tables 2-3.

Supplementary Table 2. Permutation test confirms significant overexpression of many CEN/KT genes in different cancer types.

CES Genes	GSE6791 cervical		GSE3744		GSE4290		GSE6764		GSE8761		GSE10780		GSE12452	
	obs. changes	perm. Freq (%)	obs. changes	perm. Freq (%)	obs. changes	perm. Freq (%)	obs. changes	perm. Freq (%)	obs. changes	perm. Freq (%)	obs. changes	perm. Freq (%)	obs. changes	perm. Freq (%)
CENP-A	2.51	0	4.41	0	1.82	0	3.17	0	1.61	0	1.44	0	1.27	0
CENP-K	3.06	0	1.86	0	2.39	0	2.27	0	1.56	0	1.13	0	1.48	0
CENP-L	1.09	0	1.29	0	0.86	0	1.47	0	0.79	0	0.33	0	0.65	0.001
CENP-M	0.82	0	1.65	0	1.52	0	1.73	0	0.75	0	0.75	0	0.27	0.096
CENP-N	1.61	0	2.33	0	1.24	0	1.95	0	1.50	0	0.72	0	1.00	0
CENP-W	2.79	0	1.60	0.006	0.37	0.041	2.74	0	1.57	0	0.88	0	0.97	0.002
HJURP	1.26	0	3.03	0	2.39	0	2.23	0	1.65	0	1.02	0	0.63	0
CENP-U	1.72	0	3.22	0	1.92	0	2.62	0	1.09	0	2.08	0	1.69	0
NDC80	3.35	0	2.81	0	4.52	0	2.81	0	1.47	0	1.26	0	1.97	0
NUF2	3.70	0	4.19	0	2.29	0	3.54	0	1.55	0	1.42	0	2.06	0
MIS18B	2.13	0	2.58	0	1.28	0	2.39	0	1.45	0	0.92	0	1.48	0
SPC24	0.80	0.002	1.70	0	2.07	0	1.12	0	0.63	0.001	0.40	0	0.59	0.001
SPC25	1.40	0	2.22	0	1.25	0	2.17	0	1.32	0	0.71	0	1.07	0.001
ZWINT	1.90	0	2.87	0	0.86	0	3.02	0	1.10	0	2.18	0	1.52	0

CES Genes	GSE13911		GSE14407		GSE16515		GSE19188		GSE21422		GSE31210		GSE6791 head and neck	
	obs. changes	perm. Freq (%)	obs. changes	perm. Freq (%)	obs. changes	perm. Freq (%)	obs. changes	perm. Freq (%)	obs. changes	perm. Freq (%)	obs. changes	perm. Freq (%)	obs. changes	perm. Freq (%)
CENP-A	1.13	0	3.34	0	1.72	0	2.89	0	3.64	0.005	1.79	0	1.09	0
CENP-K	0.77	0	2.72	0	1.86	0	1.72	0	2.38	0.001	1.05	0	0.86	0.002
CENP-L	0.90	0	1.34	0.001	0.55	0	0.97	0	1.48	0	0.98	0	0.63	0
CENP-M	1.73	0	2.01	0.016	1.14	0	1.37	0	1.39	0.003	1.77	0	0.56	0.001
CENP-N	1.49	0	2.23	0	0.77	0.001	1.91	0	1.78	0.043	0.85	0	0.87	0
CENP-W	1.05	0	0.58	0.113	1.32	0	1.71	0	1.80	0.001	0.54	0	1.16	0
HJURP	1.73	0	2.03	0.009	0.93	0	2.32	0	1.61	0.011	1.57	0	0.32	0.007
CENP-U	0.67	0	2.05	0	1.87	0	2.18	0	3.25	0.002	1.30	0	0.23	0.259
NDC80	1.64	0	1.74	0.004	1.51	0	2.58	0	3.13	0.001	1.07	0	1.26	0.001
NUF2	2.00	0	2.92	0	1.79	0	2.98	0	3.01	0.004	1.69	0	1.02	0.001
MIS18B	0.99	0	2.48	0	1.24	0	1.98	0	2.40	0.003	1.08	0	1.00	0
SPC24	2.04	0	3.50	0	0.33	0.021	0.99	0	0.82	0.046	1.53	0	0.18	0.113
SPC25	1.55	0	2.84	0	0.61	0.007	1.86	0	1.80	0.011	1.44	0	0.72	0.001
ZWINT	0.74	0	3.27	0	1.64	0	2.31	0	3.72	0	1.54	0	0.76	0

obs. changes = observed fold difference for log2 (mRNA intensity) between normal and cancer samples. perm. Freq = frequency of gene overexpression higher than the observed value in cancers from 1,000 repetitions of randomization, i.e., *p*-value. Significant $p<0.05$.

Supplementary Table 3. CEN/KT genes are prognostic for breast cancer patient survival using BC GenExMiner database.

Genes	Breast Cancer AEFS		Breast Cancer MRFS	
	HR (95%CI)	p-value	HR (95%CI)	p-value
CENP-A	1.47 (1.31-1.66)	<0.0001	1.56 (1.33-1.83)	<0.0001
HJURP	1.55 (1.37-1.76)	<0.0001	1.69 (1.43-1.98)	<0.0001
M18BP1	0.96 (0.85-1.09)	0.567	0.93 (0.80-1.09)	0.400
MIS18A	1.34 (1.18-1.51)	<0.0001	1.48 (1.26-1.74)	<0.0001
MIS18B	1.42 (1.26-1.61)	<0.0001	1.51 (1.28-1.77)	<0.0001
CENP-C	0.82 (0.75-0.91)*	0.0001	0.78 (0.69-0.88)*	0.0001
CENP-N	1.61 (1.46-1.77)	<0.0001	1.82 (1.60-2.07)	<0.0001
CENP-I	1.44 (1.28-1.63)	<0.0001	1.46 (1.25-1.72)	<0.0001
CENP-H	1.27 (1.08-1.50)	0.005	1.32 (1.01-1.72)	0.041
CENP-T	1.02 (0.90-1.17)	0.721	1.08 (0.91-1.28)	0.392
CENP-W	1.95 (1.40-2.72)	0.0001	2.13 (1.41-3.19)	0.0003
CENP-S	1.14 (0.98-1.32)	0.090	1.10 (0.91-1.32)	0.331
CENP-X	1.22 (1.12-1.34)	0.0001	1.20 (1.06-1.36)	0.004
CENP-M	1.39 (1.23-1.57)	<0.0001	1.49 (1.27-1.75)	<0.0001
CENP-U	1.35 (1.19-1.52)	<0.0001	1.51 (1.29-1.77)	<0.0001
CENP-L	1.44 (1.22-1.70)	<0.0001	1.43 (1.15-1.79)	0.001
CENP-K	1.28 (1.10-1.48)	0.001	1.34 (1.10-1.65)	0.005
CENP-O	1.31 (1.16-1.48)	<0.0001	1.28 (1.09-1.50)	0.002
CENP-P	0.96 (0.79-1.16)	0.658	0.88 (0.69-1.13)	0.327
CENP-Q	0.99 (0.88-1.12)	0.870	0.98 (0.84-1.15)	0.835
CENP-R	0.96 (0.85-1.08)	0.469	0.90 (0.77-1.05)	0.165
KNL1	1.19 (1.08-1.32)	0.001	1.21 (1.07-1.38)	0.003
ZWINT	1.51 (1.37-1.67)	<0.0001	1.66 (1.46-1.89)	<0.0001
MIS12	1.14 (1.00-1.30)	0.045	1.17 (0.98-1.39)	0.077
PMF1	0.96 (0.87-1.05)	0.381	0.99 (0.87-1.12)	0.827
NSL1	1.02 (0.90-1.14)	0.796	1.03 (0.88-1.21)	0.679
KNL3	1.26 (1.11-1.42)	0.0003	1.44 (1.23-1.70)	<0.0001
NDC80	1.32 (1.17-1.48)	<0.0001	1.38 (1.18-1.62)	<0.0001
SPC24	1.54 (1.25-1.88)	<0.0001	1.66 (1.20-2.30)	0.002
SPC25	1.32 (1.17-1.48)	<0.0001	1.36 (1.16-1.59)	0.0001
NUF2	1.32 (1.14-1.54)	0.0003	1.41 (1.13-1.76)	0.003

Genes are highlighted according to the color scheme in Figure 1B. Cells for CEN/KT genes with significant prognostic values (p<0.05) for breast cancer patient any event (AE)-free survival and metastatic relapse (MR)-free survival are highlighted (red for overexpression and green for reduced expression), Hazard ratio (HR) with 95% confidence interval, and p-values are shown.

Supplementary Table 4. CEN/KT genes are prognostic for breast cancer patient survival using K-M Plotter database.

	BREAST CANCER OS (n=1115)		BREAST CANCER RFS (n=3455)		BREAST CANCER DMFS (n=1609)	
	HR (95% CI)	p-value	HR (95% CI)	p-value	HR (95% CI)	p-value
CENP-A	1.89 (1.49-2.42)	1.6E-07	1.97 (1.75-2.22)	0.0E+00	1.87 (1.52-2.3)	1.9E-09
HJURP	1.73 (1.36-2.2)	7.1E-06	1.72 (1.53-1.93)	0.0E+00	1.87 (1.52-2.29)	1.9E-09
M18BP1	1.07 (0.85-1.36)	5.5E-01	1.3 (1.16-1.46)	6.2E-06	1.3 (1.06-1.59)	1.1E-02
MIS18A	1.75 (1.38-2.23)	4.1E-06	1.16 (1.03-1.3)	1.3E-02	1.82 (1.48-2.24)	7.5E-09
MIS18B	1.89 (1.49-2.41)	1.5E-07	1.43 (1.27-1.61)	1.3E-09	1.62 (1.33-1.99)	2.5E-06
CENP-C	0.88 (0.69-1.12)	3.0E-01	0.71 (0.63-0.8)	8.1E-09	0.91 (0.74-1.11)	3.4E-01
CENP-N	1.85 (1.45-2.36)	4.9E-07	1.95 (1.73-2.19)	0.0E+00	1.8 (1.46-2.21)	1.7E-08
CENP-I	1.53 (1.2-1.94)	5.1E-04	1.45 (1.29-1.62)	3.3E-10	1.76 (1.44-2.17)	4.3E-08
CENP-H	NA	NA	NA	NA	NA	NA
CENP-T	0.92 (0.72-1.17)	4.9E-01	0.73 (0.65-0.83)	1.7E-07	1.09 (0.89-1.33)	4.2E-01
CENP-W	NA	NA	NA	NA	NA	NA
CENP-S	1.09 (0.86-1.39)	4.7E-01	1.1 (0.98-1.24)	9.2E-02	0.92 (0.75-1.12)	4.0E-01
CENP-X	1.07 (0.84-1.36)	5.7E-01	1.39 (1.24-1.56)	2.3E-08	1.28 (1.05-1.57)	1.7E-02
CENP-M	1.55 (1.22-1.97)	3.3E-04	1.49 (1.32-1.67)	1.7E-11	1.4 (1.14-1.72)	1.0E-03
CENP-U	1.75 (1.38-2.23)	3.9E-06	1.98 (1.76-2.23)	0.0E+00	1.79 (1.46-2.2)	2.0E-08
CENP-L	NA	NA	NA	NA	NA	NA
CENP-K	NA	NA	NA	NA	NA	NA
CENP-O	0.92 (0.72-1.16)	4.8E-01	0.8 (0.71-0.9)	1.2E-04	0.93 (0.76-1.14)	5.1E-01
CENP-P	NA	NA	NA	NA	NA	NA
CENP-Q	1.05 (0.83-1.33)	7.0E-01	1.14 (1.02-1.28)	2.4E-02	1.14 (0.93-1.39)	2.2E-01
CENP-R	1.06 (0.84-1.35)	6.2E-01	1.17 (1.05-1.32)	6.5E-03	0.94 (0.77-1.15)	5.8E-01
KNL1	0.98 (0.77-1.24)	8.6E-01	0.86 (0.76-0.96)	9.7E-03	0.97 (0.79-1.18)	7.4E-01
ZWINT	1.55 (1.22-1.97)	2.8E-04	1.7 (1.51-1.91)	0.0E+00	1.56 (1.27-1.91)	1.7E-05
MIS12	0.94 (0.74-1.2)	6.4E-01	1.05 (0.93-1.17)	4.4E-01	0.99 (0.81-1.21)	9.1E-01
NSL1	0.94 (0.74-1.19)	5.8E-01	1.2 (1.07-1.35)	2.2E-03	1.09 (0.89-1.34)	3.9E-01
PMF1	0.78 (0.61-0.99)	4.0E-02	0.92 (0.82-1.03)	1.5E-01	0.9 (0.73-1.1)	3.0E-01
KNL3	1.36 (1.07-1.72)	1.2E-02	1.36 (1.21-1.52)	2.3E-07	1.31 (1.07-1.61)	8.0E-03
NDC80	1.51 (1.19-1.91)	6.8E-04	1.87 (1.66-2.11)	0.0E+00	1.82 (1.48-2.24)	6.4E-09
SPC24	NA	NA	NA	NA	NA	NA
SPC25	1.49 (1.18-1.9)	9.4E-04	1.44 (1.28-1.61)	8.3E-10	1.55 (1.26-1.9)	2.1E-05
NUF2	NA	NA	NA	NA	NA	NA

Genes are highlighted according to the color scheme in Figure 1B. Significant genes are highlighted (red for overexpression and green for reduced expression, $p<0.05$). Hazard ratio (HR) with 95% confidence interval, and *p*-values are shown. OS = overall survival, RFS = relapse-free survival, DMFS = distant metastasis-free survival. NA = not applicable. NA indicates lack of probes in the U133A platform. For genes with more than one probes, the most sensitive probes and associated values are presented.

Supplementary Table 5. CEN/KT genes are prognostic for lung cancer patient overall survival and first progression using K-M Plotter database.

Genes	LUNG CANCER OS (n=1405)		LUNG CANCER FP (n=982)	
	HR (95% CI)	p-value	HR (95% CI)	p-value
CENP-A	1.57 (1.35-1.83)	5.6E-09	1.87 (1.51-2.31)	6.0E-09
HJURP	1.73 (1.49-2.02)	1.2E-12	1.96 (1.61-2.38)	6.7E-12
M18BP1	0.74 (0.63-0.86)	8.4E-05	0.61 (0.49-0.75)	3.8E-06
MIS18A	1.38 (1.18-1.61)	3.2E-05	1.37 (1.11-1.7)	3.1E-03
MIS18B	1.67 (1.43-1.94)	3.8E-11	1.81 (1.46-2.24)	3.2E-08
CENP-C	0.62 (0.53-0.72)	5.5E-10	0.5 (0.4-0.62)	1.4E-10
CENP-N	1.47 (1.27-1.72)	5.6E-07	1.62 (1.31-2.01)	6.9E-06
CENP-I	1.41 (1.21-1.64)	8.3E-06	1.73 (1.4-2.14)	3.5E-07
CENP-H	NA	NA	NA	NA
CENP-T	1.34 (1.15-1.56)	1.5E-04	1.56 (1.26-1.93)	3.9E-05
CENP-W	NA	NA	NA	NA
CENP-S	0.71 (0.61-0.83)	1.1E-05	0.64 (0.52-0.79)	3.6E-05
CENP-X	1.55 (1.33-1.8)	1.8E-08	1.74 (1.4-2.15)	3.0E-07
CENP-M	1.55 (1.33-1.8)	1.3E-08	1.53 (1.24-1.89)	7.5E-05
CENP-U	1.7 (1.46-1.98)	8.6E-12	1.57 (1.27-1.94)	2.6E-05
CENP-L	NA	NA	NA	NA
CENP-K	NA	NA	NA	NA
CENP-O	1.19 (1.02-1.38)	2.5E-02	1.52 (1.23-1.88)	8.6E-05
CENP-P	NA	NA	NA	NA
CENP-Q	0.82 (0.71-0.96)	1.2E-02	0.86 (0.69-1.08)	2.0E-01
CENP-R	1 (0.86-1.17)	3.6E-01	1.1 (0.9-1.36)	3.5E-01
KNL1	0.93 (0.8-1.08)	1.2E-01	0.77 (0.6-1)	5.0E-02
ZWINT	1.5 (1,32-1.71)	3.2E-10	1.52 (1.16-1.99)	2.4E-03
MIS12	0.73 (0.63-0.85)	4.9E-05	0.62 (0.5-0.77)	8.7E-06
NSL1	0.68 (0.59-0.8)	9.4E-07	0.64 (0.52-0.79)	2.8E-05
PMF1	0.86 (0.76-0.97)	1.7E-02	1.01 (0.83-1.22)	9.4E-01
KNL3	1.08 (0.93-1.25)	3.3E-01	1.09 (0.88-1.34)	4.4E-01
NDC80	1.29 (1.11-1.51)	8.9E-04	1.21 (0.98-1.5)	7.1E-02
SPC24	NA	NA	NA	NA
SPC25	1.69 (1.45-1.97)	1.3E-11	1.99 (1.61-2.47)	1.4E-10
NUF2	NA	NA	NA	NA

Genes are highlighted according to the color scheme in Figure 1B. Significant genes are highlighted (red for overexpression and green for reduced expression, $p<0.05$). Hazard ratio (HR) with 95% confidence interval, and p values are shown. OS = overall survival, FP = first progression. NA = not applicable. NA indicates lack of probes in the U133A platform. For genes with more than one probes, the most significant probes and associated values are presented.

Supplementary Table 6. CEN/KT genes are prognostic for early stage ovarian cancer patient overall survival and progression-free survival using K-M Plotter.

Genes	OVARIAN CANCER OS (n=133)		OVARIAN CANCER PFS (n=126)	
	HR (95% CI)	p-value	HR (95% CI)	p-value
CENP-A	9.2 (1.24-68.13)	8.2E-03	17.24 (2.37-125.29)	1.1E-04
HJURP	5.17 (1.54-17.35)	3.0E-03	3.12 (1.7-5.73)	1.1E-04
M18BP1	3.65 (1.37-9.75)	5.6E-03	3.03 (1.28-7.16)	8.1E-03
MIS18A	7.59 (1.03-56.14)	1.9E-02	3.24 (1.71-6.13)	1.3E-04
MIS18B	4.78 (1.13-20.28)	1.9E-02	4.63 (1.65-12.95)	1.3E-03
CENP-C	0.43 (0.19-0.96)	3.4E-02	3.34 (1.84-6.06)	2.7E-05
CENP-N	5.17 (1.54-17.29)	3.0E-03	2.36 (1-5.6)	4.4E-02
CENP-I	3.85 (1.15-12.88)	1.8E-02	4.13 (1.63-10.51)	1.2E-03
CENP-H	NA	NA	NA	NA
CENP-T	1.49 (0.66-3.38)	3.4E-01	1.35 (0.71-2.55)	3.6E-01
CENP-W	NA	NA	NA	NA
CENP-S	1.49 (0.66-3.33)	3.3E-01	1.81 (0.99-3.3)	4.9E-02
CENP-X	1.84 (0.83-4.11)	1.3E-01	0.49 (0.25-0.95)	3.1E-02
CENP-M	4.36 (1.3-14.59)	9.2E-03	2.96 (1.37-6.38)	3.8E-03
CENP-U	4.94 (1.16-20.97)	1.6E-02	1.93 (1.06-3.5)	2.9E-02
CENP-L	NA	NA	NA	NA
CENP-K	NA	NA	NA	NA
CENP-O	1.97 (0.89-4.35)	8.8E-02	0.73 (0.39-1.37)	3.3E-01
CENP-P	NA	NA	NA	NA
CENP-Q	1.92 (0.86-4.28)	1.1E-01	1.88 (1.02-3.46)	3.8E-02
CENP-R	0.38 (0.13-1.11)	6.6E-02	1.33 (0.74-2.41)	3.4E-01
KNL1	1.66 (0.69-3.97)	2.5E-01	0.47 (0.23-0.93)	2.6E-02
ZWINT	2.6 (1.14-5.9)	1.8E-02	4.03 (2.02-8)	1.8E-05
MIS12	3.79 (1.13-12.71)	2.0E-02	1.83 (0.98-3.42)	5.4E-02
NSL1	3.21 (1.27-8.07)	9.1E-03	3.83 (2.07-7.08)	4.6E-06
PMF1	3.36 (1.44-7.84)	2.9E-03	2.86 (1.57-5.19)	3.2E-04
KNL3	4.16 (1.53-11.29)	2.6E-03	3.92 (2-7.68)	2.0E-05
NDC80	5.15 (2.03-13.09)	1.4E-04	3.94 (1.99-7.78)	2.4E-05
SPC24	NA	NA	NA	NA
SPC25	2.98 (1.24-7.16)	1.0E-02	4.17 (1.63-10.63)	1.2E-03
NUF2	NA	NA	NA	NA

Genes are highlighted according to the color scheme in Figure 1B. Early stage ovarian cancers include both stage I and II tumors. Significant genes are highlighted (red for overexpression and green for reduced expression, *p*<0.05). Hazard ratio (HR) with 95% confidence interval, and *p*-values are shown. OS = overall survival. PFS = progression-free survival. NA = Not Applicable. NA indicates lack of probes for the gene.

Supplementary Table 7. CEN/KT genes are prognostic for gastric cancer patient overall survival and first progression using K-M Plotter.

Genes	GASTRIC CANCER OS (n=593)		GASTRIC CANCER FP (n=359)	
	HR (95% CI)	p-value	HR (95% CI)	p-value
CENP-A	1.5 (1.23-1.82)	5.9E-05	1.86 (1.45-2.37)	5.0E-07
HJURP	1.7 (1.36-2.12)	2.6E-06	2.23 (1.74-2.86)	7.2E-11
M18BP1	1.18 (0.95-1.47)	1.4E-01	0.73 (0.56-0.96)	2.5E-02
MIS18A	1.29 (1.04-1.6)	1.9E-02	1.33 (1.04-1.7)	2.4E-02
MIS18B	1.28 (1.05-1.56)	1.5E-02	1.55 (1.18-2.03)	1.5E-03
CENP-C	0.78 (0.64-0.96)	1.8E-02	0.8 (0.63-1.03)	8.4E-02
CENP-N	1.54 (1.26-1.89)	2.9E-05	2.06 (1.53-2.76)	8.8E-07
CENP-I	1.52 (1.25-1.84)	2.2E-05	1.75 (1.36-2.24)	7.7E-06
CENP-H	NA	NA	NA	NA
CENP-T	1.15 (0.95-1.39)	1.6E-01	1.61 (1.25-2.08)	2.0E-04
CENP-W	NA	NA	NA	NA
CENP-S	1.44 (1.16-1.78)	1.0E-03	1.37 (1.07-1.75)	1.2E-02
CENP-X	0.67 (0.54-0.84)	3.0E-04	0.56 (0.43-0.74)	2.7E-05
CENP-M	1.38 (1.1-1.73)	5.6E-03	1.51 (1.18-1.93)	9.5E-04
CENP-U	1.48 (1.19-1.85)	4.4E-04	1.59 (1.22-2.06)	4.6E-04
CENP-L	NA	NA	NA	NA
CENP-K	NA	NA	NA	NA
CENP-O	1.16 (0.94-1.43)	1.6E-01	1.53 (1.2-1.96)	5.9E-04
CENP-P	NA	NA	NA	NA
CENP-Q	1.59 (1.29-1.96)	1.3E-05	1.52 (1.14-2.02)	4.2E-03
CENP-R	1.43 (1.16-1.77)	7.5E-04	1.66 (1.29-2.13)	6.8E-05
KNL1	0.82 (0.66-1.01)	6.6E-02	0.8 (0.61-1.05)	1.1E-01
ZWINT	1.39 (1.13-1.72)	1.8E-03	1.56 (1.22-2)	3.0E-04
MIS12	1.28 (1.04-1.57)	1.8E-02	1.33 (1.02-1.74)	3.7E-02
NSL1	0.88 (0.71-1.08)	2.2E-01	0.74 (0.57-0.95)	1.7E-02
PMF1	0.82 (0.67-1)	5.2E-02	0.71 (0.55-0.91)	6.7E-03
KNL3	1.24 (1.02-1.5)	3.0E-02	1.35 (1.05-1.75)	2.1E-02
NDC80	1.54 (1.26-1.89)	2.1E-05	1.88 (1.44-2.45)	2.0E-06
SPC24	NA	NA	NA	NA
SPC25	1.45 (1.19-1.77)	2.1E-04	1.94 (1.45-2.58)	4.6E-06
NUF2	NA	NA	NA	NA

Genes are highlighted according to the color scheme in Figure 1B. Significant genes are highlighted (red for overexpression and green for reduced expression, $p<0.05$). Hazard ratio (HR) with 95% confidence interval, and *p*-values are shown. OS = overall survival. FP = first progression. NA = Not Applicable. NA indicates absence of probes.

Supplementary Table 8. Comparison of gene lists from Supplementary Tables 3-7.

Genes	breast cancer (BC GeneEx Miner)	breast cancer (K-M plotter)	lung Cancer (K-M Plotter)	gastric cancer (K-M Plotter)	DE genes	CES Genes
CENP-A	x	x	x	x	x	**x**
HJURP	x	x	x	x	x	**x**
M18BP1		x	x	x		
MIS18A	x	x	x	x		
MIS18B	x	x	x	x	x	**x**
CENP-C	x	x	x	x		
CENP-N	x	x	x	x	x	**x**
CENP-I	x	x	x	x		
CENP-H	x	NA	NA	NA		
CENP-T		x	x	x		
CENP-W	x	NA	NA	NA	x	**x**
CENP-S			x	x		
CENP-X	x	x	x	x		
CENP-M	x	x	x	x	x	**x**
CENP-U	x	x	x	x	x	**x**
CENP-L	x	NA	NA	NA	x	**x**
CENP-K	x	NA	NA	NA	x	**x**
CENP-O	x	x	x	x		
CENP-P		NA	NA	NA		
CENP-Q		x	x	x		
CENP-R		x		x		
KNL1	x	x			x	
ZWINT	x	x	x	x	x	**x**
MIS12			x	x		
NSL1		x	x	x		
PMF1		x	x	x		
KNL3	x	x		x		
NDC80	x	x	x	x	x	**x**
SPC24	x	NA	NA	NA	x	**x**
SPC25	x	x	x	x	x	**x**
NUF2	x	NA	NA	NA	x	**x**

Genes are highlighted according to the color scheme in Figure 1B. The 14 CEN/KT genes that are consistently misexpressed and with significant prognostic values in various cancers are defined as CES genes. NA = Not Applicable as indicated for Tables S4-S7. Black checkmarks (x) indicate overexpression. Green checkmarkes highlighed in grey indicate reduced expression. Cells of CES genes are checked in bold and highlighted in red.

Supplementary Table 9. Combined mutation frequencies for the 14 CES genes in various TCGA cancer datasets analyzed using cBioPortal.

Cancer Type	Mutation Frequency	
AML	0.50%	(1/200)
adenoid cystic carcinoma	0.00%	(0/60)
adrenocortical carcinoma	3.33%	(3/90)
bladder urothelial carcinoma	13.08%	(17/130)
brain lower grade glioma	1.40%	(4/286)
breast invasive carcinoma	2.24%	(22/982)
cervical cancer	6.70%	(13/194)
colorectal adenocarcinoma	8.04%	(18/224)
esophageal adenocarcinoma	4.11%	(6/146)
esophageal squamous cell carcinoma	5.68%	(5/88)
glioblastoma	0.34%	(1/291)
glioblastoma multiforme	1.38%	(4/290)
head and neck carcinoma	3.58%	(10/279)
kidney chromophobe	1.52%	(1/66)
kidney renal clear cell carcinoma	2.88%	(12/417)
kidney papillary cell carcinoma	5.59%	(9/161)
liver heptacellular carcinoma	3.03%	(6/198)
lung adenocarcinoma	10.87%	(25/230)
lung squamous cell carcinoma	6.74%	(12/178)
melanoma	12.00%	(3/25)
multiple myeloma	1.95%	(4/205)
nasopharyngeal carcinoma	3.57%	(2/56)
ovarian serous cystadenocarcinoma	1.90%	(6/316)
pancreatic adenocarcinoma	4.11%	(6/146)
papillary thyroid carcinoma	0.50%	(2/401)
prostate carcinoma	0.94%	(4/425)
metastatic prostate carcinoma	4.92%	(3/61)
skin cutaneous melanoma	10.14%	(35/345)
small cell lung cancer	8.45%	(6/71)
stomach adenocarcinoma	11.07%	(32/289)
thyroid carcinoma	0.49%	(2/405)
uterine carcinoma	3.51%	(2/57)
uterine corpus endometrial carcinoma	10.89%	(27/248)

Supplementary Table 10. Correlation between CES values and CNA and mutation frequencies in breast cancer IDCs and ILCs in TCGA breast adenocarcinoma dataset.

Breast Cancer Type	Mutation Frequency			Copy Number Alteration		
	Spearman's rho	p-value	N	Spearman's rho	p-value	N
IDC	0.495	<2.2E-16	396	0.471	<2.2E-16	396
ILC	0.275	0.016	76	0.452	4.2E-05	76
Luminal A IDC	0.251	0.001	396	0.207	0.007	396
Luminal A ILC	0.136	0.283	76	0.330	0.007	76

Significant two-tailed *p*-values for Spearman's correlation coefficient are highlighted in yellow ($p<0.05$). IDC = invasive ductal carcinoma. ILC = invasive lobular carcinoma.

Supplementary Table 11. Biased distribution of breast cancer molecular subtypes between stage I and II in TCGA breast adenocarcinoma dataset.

TCGA breast		stage I (n=98)		stage II (n=313)	
PAM50 [a]	Basal-like	15	15.3%	61	19.5%
	HER2+	3	3.1%	26	8.3%
	Luminal A	60	61.2%	150	47.9%
	Luminal B	20	20.4%	69	22.0%
	Normal	0	0.0%	7	2.2%

p=0.086, Fisher's exact test. Tumor purity cutoff is 0.3.

Supplementary Table 12. Sample characteristics of breast cancer datasets used to for survival and correlation analysis.

	Breast Cancer Datasets						
Factors	GSE6532 (n=246)	GSE20685 (n=297)	GSE1456 (n=147)	GSE3494 (n=222)	NKI (n=295)	Gray (n=130)	TCGA (n=624)
**Age (years)**			-				
Minimal	32	24	-	28	26	31	26
Median	60	47	-	63	44	51	59
Maximal	88	84	-	90	53	88	90
Unknown	0	0	-	0	0	1	0
**Grade**		-					-
1	58	-	28	58	101	14	-
2	134	-	58	117	119	46	-
3	54	-	61	47	75	65	-
Unknown	0	-	0	0	0	5	-
**Stage**	-	-	-	-	-		
I	-	-	-	-	-	33	110
II	-	-	-	-	-	75	363
III	-	-	-	-	-	14	134
IV	-	-	-	-	-	5	6
Unknown	-	-		-	-	3	11
**Stage T**	-		-	-			
T1	-	100	-	-	155	52	172
T2	-	175	-	-	140	61	355
T3	-	15	-	-	0	8	71
T4	-	7	-	-	0	6	25
Unknown		0		-	0	3	1
**Tumor Size (cm)**		-	-				-
Min. Size	0.0	-	-	0.2	0.2	0.3	-
Median Size	2.1	-	-	2.0	2.0	2.3	-
Max. Size	7	-	-	6.5	5.0	7.5	-
Unknown	0.0	-	-	0.0	0.0	2.0	-
**Lymph node**			-				
Negative	172	133	-	145	151	59	298
Positive	74	164	-	77	144	66	317
Unknown	0	0	-	0	0	5	9
**ER status**		-	-				-
ER-	37	-	-	29	69	46	-
ER+	209	-	-	193	226	84	-
Unknown	0	-	-	0	0	0	-
**PR status**		-	-		-		-
PR-	11	-	-	50	-	54	-
PR+	114	-	-	172	-	74	-
Unknown	121	-	-	0	-	2	-
**Event 1**	**RFS**	**RFS**	**RFS**	**DFS**	**Recurrence**	**OS**	**DFS**
No	157	259	108	168	181	84	528
Yes	89	19	38	54	114	45	39
Unknown	0	19	0	0	0	1	57
Median Time from Diagnosis (yrs.)	6.2	4.9	7.1	10.3	6.7	6.0	1.6
**Event 2**	**DMFS**	**OS**	**OS**		**DMFS**	**DFS**	**OS**
No	187	234	112		194	100	549
Yes	59	63	35		101	29	75
Unknown	0	0	0		0	1	0
Median Time from Diagnosis (yrs.)	6.3	8.3	7.1		6.8	6.0	1.8
**Event 3**					**OS**		
No					216		
Yes					79		
Unknown					0		
Median Time from Diagnosis (yrs.)					7.2		
**Therapy**			-	-			-
	104 (untreated)	- (≤ 54) (untreated)	-	-	165 (untreated)	61 (no RT)	-
	92 (tamoxifen)	87 (CAF)	-	-	90 (chemo only)	67 (RT)	-
	50 (tamoxifen-only)	61 (CMF)	-	-	20 (hormone only)	60 (no chemo)	-
		91 (other chemo.)	-	-	20 (chemo&hormone)	68 (chemo)	-
		- (radiotherapy)	-	-		53 (no Hormone)	-
		4 (unknown)	-	-		74 (Hormone)	-
			-	-		3 (unknown)	-
**Platform**	HG-U133-Plus2	HG-U133-Plus2	HG-U133-Plus2	HG-U133-Plus2	Agilent Hu25K	HG-U133A	RNA-seq

Supplementary Table 13. Sample characteristics of lung cancer datasets used to for survival and correlation analysis.

Factors	lung cancer datasets[a]					
	GSE14814 (JBR.10) (n=133)	GSE42127 (n=176)	GSE31210 (n=204)	GSE37745 (n=196)	TCGA lung ADC (n=220)	TCGA lung SCC (n=426)
**Age (years)**						
Minimal	35	42	30	39	41	39
Median	62	66	61	65	67	68
Maximal	81	86	76	84	86	85
Uknown	0	0	0	0	16	8
**Gender**						
Female	42	83	109	89	125	110
Male	91	93	95	107	95	316
Unknown	0	0	0	0	0	0
**Smoking History**						
No	-	-	105	-	32	-
Yes	-	-	99	-	178	417
Unknown	133	176	0	-	10	9
**Stage**						
Stage 1	73	112	162	130	115	205
Stage 2	60	32	42	35	46	136
Stage 3	0	30	0	27	45	77
Stage 4	0	1	0	4	9	7
Unknown	0	1	0	0	5	1
**Lymph Node Status**	-	-	-	-	-	
Negative	-	-	-	-	-	269
Positive	-	-	-	-	-	153
Unknown	-	-	-	-	-	4
**Stage T**	-	-	-	-		
T1	-	-	-	-	60	93
T2	-	-	-	-	128	245
T3	-	-	-	-	17	64
T4	-	-	-	-	14	22
Unknown	-	-	-	-	1	0
**Histological subtype**						
ADC	71	133	204	104	220	0
SCC	52	43	0	66	0	426
LCC/NOS	10	0	0	24	0	0
Unknown	0	0	0	0	0	0
**Event 1**	**OS**	**OS**	**OS**	**OS**	**OS**	**OS**
No	73	112	174	51	154	245
Yes	60	64	30	145	66	181
Unknown	0	0	0	0	0	6
Median Follow-up (years)	5.3	4	5.0	3.4	1.1	1.9
**Event 2**	**DSS**		**Relapse**	**Relapse**		**DFS**
No	83		150	47		215
Yes	50		54	49		106
Unknown	0		0	100		105
Median Follow-up (years)	5.3		4.7	1.7		1.8
**Adjuvant Chemotherapy**						
untreated	62	127	204	71	-	-
treated	71 (ACT)	49 (ACT)	0	29 (unspecified)	-	-
Uknown	0	0	0	96	220	426
**Platform**	HG-U133A	Illumina HumanWG-6 v3	HG-U133PLUS2	HG-U133PLUS2	RNA-seq	RNA-seq

[a] Datasets or subcohorts after removing samples due to missingness used for correlation, Kaplan-Meier survival estimation, or multivariate Cox regression.

Supplementary Table 14. Lists of breast, lung, ovarian and gastric cancer datasets in K-M Plotter database used for Kaplan-Meier survival analysis.

Breast Cancer	Lung Cancer	Ovarian Cancer	Gastric Cancer
E-MTAB-365 (n=537)	CAARRAY (n=504)	GSE14764 (n=80)	GSE14210 (n=146)
GSE11121 (n=200)	GSE14814 (n=90)	GSE15622 (n=36)	GSE15459 (n=200)
GSE12093 (n=136)	GSE19188 (n=157)	GSE18520 (n=63)	GSE22377 (n=43)
GSE12276 (n=204)	GSE29013 (n=55)	GSE19829 (n=28)	GSE29272 (n=268)
GSE1456 (n=159)	GSE30219 (n=307)	GSE23554 (n=28)	GSE51105 (n=94)
GSE16391 (n=55)	GSE31210 (n=246)	GSE26193 (n=107)	
GSE16446 (n=120)	GSE3141 (n=111)	GSE26712 (n=195)	
GSE17705 (n=196)	GSE31908 (n=40)	GSE27651 (n=49)	
GSE17907 (n=54)	GSE37745 (n=196)	GSE30161 (n=58)	
GSE19615 (n=115)	GSE43580 (n=150)	GSE3149 (n=116)	
GSE20194 (n=45)	GSE4573 (n=130)	GSE51373 (n=28)	
GSE20271 (n=96)	GSE50081 (n=181)	GSE9891 (n=285)	
GSE2034 (n=286)	GSE8894 (n=138)	TCGA (n=565)	
GSE20685 (n=327)	TCGA (n=133)		
GSE20711 (n=90)			
GSE21653 (n=240)			
GSE2603 (n=99)			
GSE26971 (n=276)			
GSE2990 (n=102)			
GSE31448 (n=71)			
GSE31519 (n=67)			
GSE3494 (n=251)			
GSE5327 (n=58)			
GSE6532 (n=82)			
GSE7390 (n=198)			
GSE9195 (n=77)			

Supplementary Table 15. Multivariate Cox regression analysis using breast cancer GSE6532 dataset.

GSE6532 (breast cancer)				
Factor	RFS (n=246)		DMFS (n=246)	
	HR (95% CI)	p-value	HR (95% CI)	p-value
CES (tertile 2 vs. tertile 1)	0.95 (0.48-1.88)	0.889	0.84 (0.37-1.91)	0.672
CES (tertile 3 vs. tertile 1)	2.59 (1.36-4.91)	0.004*	2.65 (1.26-5.56)	0.010*
Tumor Size	1.33 (1.08-1.64)	0.007*	1.26 (0.99-1.62)	0.064
Tumor Grade (2 vs. 1)	1.65 (0.75-3.65)	0.214	1.70 (0.62-4.70)	0.306
Tumor Grade (3 vs. 1)	0.88 (0.40-1.95)	0.750	1.14 (0.44-2.98)	0.790
ER status (Positive vs. Negative)	0.93 (0.49-1.75)	0.811	1.05 (0.46-2.41)	0.906
Lymph Node (Positive vs. Negative)	1.35 (0.71-2.55)	0.364	1.97 (0.90-4.28)	0.089
Treatment (Tam-only vs. Tam)	1.32 (0.57-3.10)	0.519	1.25 (0.45-3.51)	0.667
Treatment (Untreated vs. Tam)	1.06 (0.48-2.34)	0.889	1.22 (0.44-3.36)	0.697
Age	1.00 (0.98-1.02)	0.829	1.01 (0.98-1.04)	0.465

Clinical variables are listed as factors and include CES, tumor size, grade, ER status, lymph node status, patient age and treatment options. Samples with missing clinicopathological information were removed before stratification according to CES value. Significant *p*-values are marked by *. Tam = tamoxifen.

Supplementary Table 16. Multivariate Cox regression analysis using breast cancer GSE20685 dataset.

GSE20685 (breast cancer)				
Factor	OS (n=295)		DMFS (n=295)	
	Hazard Ratio (95% CI)	p-value	Hazard Ratio (95% CI)	p-value
CES (tertile 2 vs. tertile 1)	2.33 (1.12-4.86)	0.024*	1.81 (0.91-3.62)	0.091 #
CES (tertile 3 vs. tertile 1)	3.22 (1.54-6.70)	0.002*	2.54 (1.28-5.07)	0.008*
Stage N (0-3)[&]	1.61 (1.25-2.07)	0.0002*	1.74 (1.35-2.24)	1.9E-05*
Stage M (Yes vs. No)	0.60 (0.07-4.77)	0.626	NA	NA
Age	1.00 (0.97-1.02)	0.762	0.98 (0.96-1.01)	0.165

Clinical variables are listed as factors and include CES, tumor stage factor N, stage factor M and age. Samples with missing clinicopathological information were removed before stratification according to CES value. Stage T is a stratifying factor.

* Significant *p*-value.

[#] Borderline *p*-value.

[&] Stage N is treated as a continuous variable.

NA indicates small sample sizes for stage M1.

Supplementary Table 17. Multivariate Cox regression analysis using breast cancer NKI dataset.

NKI (breast cancer)						
	OS (n=295)			Recurrence (n=295)		
Factor	HR	(95% CI)	p-value	HR	(95% CI)	p-value
CES (tertile 2 vs. tertile 1)	1.60	(0.76-3.36)	0.217	1.70	(0.99 - 2.89)	0.052 #
CES (tertile 3 vs. tertile 1)	2.89	(1.36-6.15)	0.006*	2.29	(1.27 - 4.12)	0.006 *
Tumor Size	1.03	(1.00-1.05)	0.052 #	1.02	(1.00 - 1.04)	0.052 #
Tumor Grade (2 vs. 1)	3.60	(1.22-10.57)	0.020*	1.92	(1.03 - 3.61)	0.042 *
Tumor Grade (3 vs. 1)	3.96	(1.31-11.95)	0.015*	1.92	(0.98 - 3.73)	0.056 #
ER (Positive vs. Negative)	0.63	(0.38-1.07)	0.085 #	0.98	(0.61 - 1.58)	0.931
Lymph Node [1]	1.08	(0.98-1.19)	0.135	1.08	(1.00 - 1.17)	0.052 #
Chemo (Yes vs. No)	0.65	(0.37-1.15)	0.140	0.62	(0.39 - 0.98)	0.042 *
Hormone Yes vs. No)	0.68	(0.29-1.62)	0.386	0.65	(0.32 - 1.33)	0.237
Age	0.96	(0.93-1.00)	0.046*	0.95	(0.92 - 0.98)	0.001 *

Clinical variables are listed as factors and include CES, tumor size, grade, ER status, lymph node status, patient age and treatment options.

[1] Number of positive lymph nodes as a continuous variable.

* Significant *p*-values.

[#] Borderline *p*-values.

Supplementary Table 18. Multivariate Cox regression analysis using breast cancer GSE1456 dataset.

GSE1456 (breast cancer)				
	OS (n=147)		RFS (n=147)	
Factor	Hazard Ratio (95% CI)	p-value	Hazard Ratio (95% CI)	p-value
CES (tertile 2 vs. tertile 1)	18.5 (2.40-142.41)	0.005*	5.64 (1.62-19.67)	0.007*
CES (tertile 3 vs. tertile 1)	20.34 (2.46-168.4)	0.005*	4.84 (1.25-18.65)	0.022*
Tumor Grade (2 vs 1)	1.90 (0.43-8.47)	0.400	2.46 (0.55-11.01)	0.240
Tumor Grade (3 vs 1)	1.45 (0.31-6.80)	0.634	3.17 (0.67-14.94)	0.145

Clinical variables are listed as factors and include Clinical variables are CES and tumor grade.
* Significant *p*-values.

Supplementary Table 19. Multivariate Cox regression analysis using breast cancer GSE3494 dataset.

GSE3494 (breast cancer)		
Factor	DFS (n=222)	
	Hazard Ratio (95% CI)	p-value
CES (tertile 2 vs. tertile 1)	1.65 (0.75-3.66)	0.215
CES (tertile 3 vs. tertile 1)	2.27 (0.96-5.36)	0.061 #
Tumor Size	1.03 (1.01-1.06)	0.017*
Tumor Grade (2 vs. 1)	1.47 (0.62-3.47)	0.385
Tumor Grade (3 vs. 1)	1.34 (0.43-4.15)	0.615
ER status (Positive vs. Negative)	2.27 (0.76-6.79)	0.142
PR status (Positive vs. Negative)	0.63 (0.26-1.53)	0.311
Lymph Node (Positive vs. Negative)	2.65 (1.45-4.87)	0.002*
Age	1.01 (0.99-1.03)	0.578

Clinical variables are listed as factors and include CES, tumor size, grade, ER status, PR status, lymph node status and patient age.
* Significant *p*-values.
# Borderline *p*-values.

Supplementary Table 20. Multivariate Cox regression analysis using breast cancer E-TABM-158 Joe Gray dataset.

Breast cancer Joe Gray Dataset (E-TABM-158)						
	no RT subcohort OS (n=56)			with RT subcohort OS (n=58)		
Factor	HR	95% (CI)	p-value	HR	95% (CI)	p-value
CES (tertile 2 vs. tertile 1)	3.93	(0.84 - 18.26)	0.081 #	0.09	(0.01 - 1.20)	0.068 #
CES (tertile 3 vs. tertile 1)	6.71	(1.12 - 40.15)	0.037 *	0.29	(0.03 - 2.58)	0.267
Stage (1-4) [1]	3.76	(1.39 - 10.19)	0.009 *	0.86	(0.22 - 3.35)	0.833
Tumor Size	1.64	(1.11 - 2.43)	0.014 *	1.54	(0.88 - 2.71)	0.130
Age	1.02	(0.98 - 1.05)	0.360	1.01	(0.96 - 1.06)	0.764
Tumor Grade (1-3) [1]	0.65	(0.17 - 2.56)	0.541	3.98	(0.68 - 23.15)	0.125
ER (Positive vs Negative)	0.22	(0.06 - 0.82)	0.025 *	0.43	(0.09 - 2.12)	0.300
Lymph Node (Positive vs Negative)	3.14	(0.76 - 12.89)	0.113	3.57	(0.67 - 19.08)	0.136
Chemo (Yes vs. No)	0.24	(0.06 - 0.90)	0.034 *	0.87	(0.19 - 3.88)	0.852
Hormone (Yes vs. No)	0.92	(0.30 - 2.82)	0.880	1.13	(0.22 - 5.87)	0.888

Clinical variables are listed as factors and include CES, tumor stage, size, grade, ER status, lymph node status, patient age and treatment options.

[1]Continuous variables.

* Significant *p*-values.

[#] Borderline *p*-values.

Supplementary Table 21. Multivariate Cox regression analysis using stage I and stage II lung ADC GSE31210 dataset.

GSE31210 (early stage lung ADC)						
Factor	OS (n=204)			Relapse (n=204)		
	HR	(95% CI)	p-value	HR	(95% CI)	p-value
CES (tertile 2 vs. tertile 1)	3.39	(0.71 - 16.2)	0.126	3.18	(1.16 - 8.69)	0.024 *
CES (tertile 3 vs. tertile 1)	7.88	(1.76 - 35.2)	0.007 *	7.25	(2.74 - 19.17)	6E-05 *
Tumor Stage (II vs. I)	2.78	(1.32 - 5.85)	0.007 *	2.45	(1.39 - 4.34)	0.002 *
Age	1.03	(0.98 - 1.09)	0.206	1.04	(1.00 - 1.08)	0.041 *
Gender (Male vs. Female)	1.09	(0.37 - 3.19)	0.879	1.12	(0.52 - 2.41)	0.776
Smoking (Yes vs No)	1.26	(0.42 - 3.83)	0.680	0.96	(0.44 - 2.09)	0.913

Clinical variables are listed as factors and include CES, tumor stage, patient age, gender and smoking history.
* Significant *p*-values.

Supplementary Table 22. Multivariate Cox regression analysis using NSCLC GSE37745 dataset.

GSE37745 (NSCLC)						
	OS (n=196)			Recurrence (n=196)		
Factor	HR	95% CI	p-value	HR	95% CI	p-value
CES (tertile 2 vs. tertile 1)	1.95	(1.21 - 3.13)	0.006 *	2.55	(1.08 - 6.05)	0.033 *
CES (tertile 3 vs. tertile 1)	1.79	(1.06 - 3.03)	0.030 *	2.31	(0.88 - 6.08)	0.091 #
Age	1.03	(1.00 - 1.05)	0.018 *	1.00	(0.96 - 1.04)	0.992
Gender (Male vs Female)	1.04	(0.73 - 1.49)	0.830	1.29	(0.62 - 2.68)	0.503
subtype (LCC vs. ADC)	0.68	(0.38 - 1.23)	0.201	0.75	(0.28 - 2.00)	0.571
subtype (SCC vs. ADC)	0.73	(0.45 - 1.17)	0.190	0.65	(0.28 - 1.53)	0.323
WHO Performance Status (1 vs. 0)	1.88	(1.31 - 2.70)	0.001 *	3.44	(1.65 - 7.16)	0.001 *
WHO Performance Status (2 vs. 0)	1.85	(0.92 - 3.71)	0.084 #	1.01	(0.22 - 4.66)	0.992
WHO Performance Status (3 vs. 0)	2.00	(0.59 - 6.81)	0.269	3.69	(0.84 - 16.23)	0.084 #
Tumor Stage (II vs I)	1.06	(0.67 - 1.66)	0.810	0.76	(0.34 - 1.73)	0.521
Tumor Stage (III vs I)	2.00	(1.26 - 3.18)	0.003 *	2.61	(1.08 - 6.29)	0.033 *
Tumor Stage (IV vs I)	1.34	(0.41 - 4.40)	0.627	6.55	(0.72 - 59.17)	0.094 #

Clinical variables are listed as factors and include CES, tumor stage, subtype, patient WHO performance, patient age and gender.
* Significant *p*-values.
[#] Borderline *p*-values.

Supplementary Table 23. Multivariate Cox regression analysis using NSCLC GSE42127 dataset.

GSE42127 (early stage NSCLC subcohort[1])			
	OS (n=144)		
Factor	HR	(95% CI)	p-value
CES (tertile 2 vs. tertile 1)	2.39	(1.01 - 5.67)	0.048 *
CES (tertile 3 vs. tertile 1)	2.68	(1.10 - 6.53)	0.031 *
Chemo (Yes vs No)	0.53	(0.20 - 1.38)	0.195
Age	1.02	(0.99 - 1.05)	0.233
Stage M (Yes vs No)	1.28	(0.70 - 2.37)	0.424
Tumor Stage (II vs I)	1.18	(0.62 - 2.24)	0.608
Subtype (SCC vs ADC)	0.92	(0.43 - 1.99)	0.841

Clinical variables are listed as factors and include CES, tumor stage, stage factor M, subtype, treatment options and patient age.
[1] Early stage (stage I and stage II) NSCLC excluding LCCs.
* Significant *p*-values.

Supplementary Table 24. Multivariate Cox regression analysis using NSCLC GSE14814 dataset after stratifying patients according to treatment options.

NSCLC GSE14814 (JBR.10) Overall Survival				
	OBS subcohort (n=62)		ACT subcohort (n=71)	
Factor	HR (95% CI)	p-value	HR (95% CI)	p-value
CES (tertile 2 vs. tertile 1)	1.63 (0.55-4.86)	0.381	1.53 (0.62-3.78)	0.359
CES (tertile 3 vs. tertile 1)	3.51 (1.24-9.91)	0.018*	1.11 (0.36-3.46)	0.858
Stage (II vs. I)	2.89 (1.32-6.30)	0.008*	1.26 (0.60-2.63)	0.544
Histology Subtype (LCC vs. ADC)	1.05 (0.27-4.09)	0.945	0.90 (0.21-3.84)	0.885
Histology Subtype (SCC vs. ADC)	0.36 (0.16-0.84)	0.018*	0.25 (0.09-0.70)	0.009*
Gender (male vs. female)	1.63 (0.52-5.04)	0.400	1.44 (0.60-3.47)	0.410
Age	1.08 (1.01-1.14)	0.019*	1.05 (1.00-1.10)	0.050 #

Clinical variables are listed as factors and include CES, tumor stage, subtype, patient gender and age. OBS is without any adjuvant chemotherapy, ACT is adjuvant chemotherapy including cisplatin. Samples with missing clinicopathological information were removed before stratification according to CES value.
* Significant *p*-values.

Supplementary Table 25. Multivariate Cox regression analysis using NSCLC GSE14814 dataset after stratifying patients according to CES tertiles.

GSE14814 JBR.10 Trial early stage NSCLC						
	high CES subcohort		mid CES subcohort		low CES subcohort	
	OS (n=44)		OS (n=44)		OS (n=45)	
Factor	Hazard Ratio (95% CI)	p-value	Hazard Ratio (95% CI)	p-value	Hazard Ratio (95% CI)	p-value
Treatment Option (OBS vs. ACT)	2.87 (1.14-7.26)	0.027*	1.24 (0.49-3.12)	0.649	0.74 (0.23-2.36)	0.607
Stage (II vs. I)	2.30 (0.97-5.51)	0.063#	1.48 (0.54-4.03)	0.442	1.37 (0.50-3.78)	0.541
Histology Subtype (LCC vs. ADC)	1.10 (0.35-3.49)	0.874	12.06 (0.79-184.10)	0.073#	NA^	NA^
Histology Subtype (SCC vs. ADC)	0.39 (0.15-1.04)	0.060#	0.34 (0.12-0.96)	0.042*	NA^	NA^
Gender (male vs. female)	0.92 (0.25-3.44)	0.904	2.85 (0.67-12.15)	0.156	2.06 (0.61-6.92)	0.244
Age	1.08 (1.01-1.15)	0.023*	1.03 (0.96-1.10)	0.373	1.11 (1.02-1.21)	0.021*

Clinical variables are listed as factors and include tumor stage, subtype, patient gender, age and treatment options.
* Significant *p*-values.
NA^ = sample sizes are too small for regression analysis.

Supplementary Table 26. Multivariate Cox regression analysis using breast cancer meta-data in K-M Plotter database.

Factor	breast cancer OS (n=1117)		breast cancer RFS (n=3554)		breast cancer DMFS (n=1609)	
	P value	HR (95% CI)	P value	HR (95% CI)	P value	HR (95% CI)
MKI67 (High vs Low)	0.763	1.04 (0.81 - 1.34)	0.188	1.09 (0.96 - 1.23)	0.951	0.99 (0.77 - 1.28)
ER (ER+ vs ER-)	0.363	0.88 (0.67 - 1.16)	<0.0001*	0.74 (0.64 - 0.85)	0.199	0.84 (0.65 - 1.09)
HER2 (HER2+ vs HER2-)	0.047*	1.32 (1.00 - 1.73)	0.012*	1.20 (1.04 - 1.38)	0.015*	1.35 (1.06 - 1.73)
CES (High vs Low)	<0.0001*	2.20 (1.58 - 3.06)	<0.0001*	2.01 (1.70 - 2.38)	<0.0001*	1.76 (1.39 - 2.24)

Clinical variables are listed as factors and include CES, ER status, HER2 status and MKI67 level. CES is treated as categorical variable. MKI67 expression was treated as a continuous variable. OS = overall survival, RFS = relapse-free survival, DMFS = distant metastasis-free survival.
* Significant *p*-values.

Supplementary Table 27. Multivariate Cox regression analysis using NSCLC meta-data in K-M Plotter database.

Factor	NSCLC Overall Survival (n=587)		NSCLC First Progression (n=453)	
	P value	HR (95% CI)	P value	HR (95% CI)
Tumor Stage (1-4)[&]	<0.0001*	1.78 (1.46 - 2.18)	<0.0001*	2.02 (1.55 - 2.64)
Gender (M vs F)	0.113	1.29 (0.94 - 1.78)	0.189	1.29 (0.88 - 1.89)
Smoking history (Yes vs No)	0.130	0.70 (0.44 - 1.11)	0.354	1.23 (0.80 - 1.89)
CES (High vs Low)	0.0002*	1.80 (1.32 - 2.46)	0.0001*	2.28 (1.53 - 3.40)

Factor	Lung ADC Overall Survival (n=387)		Lung ADC First Progression (n=384)	
	P value	HR (95% CI)	P value	HR (95% CI)
Tumor Stage (1-4)[&]	<0.0001*	2.33 (1.69 - 3.23)	<0.0001*	2.00 (1.49 - 2.70)
Gender (M vs F)	0.726	0.92 (0.58 - 1.45)	0.832	1.05 (0.68 - 1.61)
Smoking history (Yes vs No)	0.044	0.58 (0.34 - 0.98)	0.931	0.98 (0.62 - 1.55)
CES (High vs Low)	0.018*	1.79 (1.11 - 2.88)	0.0001*	2.30 (1.53 - 3.48)

Factor	Lung SCC Overall Survival (n=189)		Lung SCC First Progression (n=189)	
	P value	HR (95% CI)	P value	HR (95% CI)
Tumor Stage (1-4)[&]	0.035*	1.34 (1.02 - 1.75)	0.039*	1.33 (1.01 - 1.74)
Gender (M vs F)	0.012*	1.83 (1.14 - 2.94)	0.013*	1.82 (1.13 - 2.93)
Smoking history (Yes vs No)	0.107	2.65 (0.81 - 8.63)	0.104	2.67 (0.82 - 8.71)
CES (High vs Low)	0.005*	1.90 (1.21 - 2.97)	0.008*	1.84 (1.18 - 2.89)

Clinical variables are listed as factors and include CES, tumor stage, patient gender and smoking history. CES is treated as a categorical variable.

[&] NSCLC stage is treated as a continuous variable.

* Significant *p*-values.

Supplementary Table 28. Cox regression analysis on breast cancer PAM50 molecular subtypes using TCGA breast adenocarcinoma dataset.

TCGA Breast Adenocarcinoma			
	OS (n=515)		
Molecular Subtypes	HR	95% CI	p-value
HER2 vs. Basal-like	4.72	(1.72 - 12.94)	0.003*
Luminal A vs. Basal-like	0.62	(0.26 - 1.47)	0.282
Luminal B vs. Basal-like	1.29	(0.52 - 3.25)	0.582
Normal-like vs. Basal-like	2.21	(0.27 - 18.15)	0.460

* Significant *p*-values.

Supplementary Table 29. Multivariate Cox regression analysis using TCGA lung ADC dataset.

TCGA lung ADC							
	OS (n=185)						
Factor	HR	95% CI				p-value	
CES (tertile 2 vs. tertile 1)	1.74	(	0.74	-	4.07)	0.203	
CES (tertile 3 vs. tertile 1)	2.50	(	1.20	-	5.17)	0.014	*
Age	1.03	(	1.00	-	1.07)	0.030	*
Gender (Male vs Female)	0.84	(	0.45	-	1.59)	0.601	
Smoking (Yes vs No)	0.88	(	0.38	-	2.04)	0.760	
Tumor Stage (II vs I)	1.95	(	0.93	-	4.07)	0.075	#
Tumor Stage (III vs I)	3.56	(	1.75	-	7.22)	4E-04	*
Tumor Stage (IV vs I)	3.20	(	1.11	-	9.23)	0.032	*

Clinical variables are listed under factor and include CES, tumor stage, patient age, gender and smoking history.
* Significant *p*-values.
[#] Borderline *p*-values.

Supplementary Table 30. Affymetrix probes of the 14 CES genes for HG-U133 PLUS 2.0 microarray.

Gene Name	Affy Probe
CENPA	204962_s_at
CENPK	222848_at
CENPL	232065_x_at
CENPM	218741_at
CENPN	219555_s_at
CENPW	226936_at
HJURP	218726_at
MLF1IP	218883_s_at
NDC80	204162_at
NUF2	223381_at
OIP5	213599_at
SPC24	235572_at
SPC25	209891_at
ZWINT	204026_s_at

Supplementary Data 1. Differential expression of 15 CEN/KT genes is significant in cancer progression across cancer types.

Supplementary Note 1. Misregulation of a subset of CEN/KT genes in cancers.

To better understand CEN/KT gene misregulation in cancers, we analyzed TCGA RNA-seq data for different types of cancer. A recent study demonstrated a strong correlation between the FoxM1 transcription factor and kinetochore gene expression, and proposed that CEN/KT genes are simultaneously up-regulated by FoxM1 in cancers[1]. Consistent with this observation, we used gene expression correlation network analyses and also detected strong correlations among many CEN/KT genes in diverse cancer types (Spearman's *rho*, r_s=0.4, p<0.05) (Supplementary Fig. 2). However, the number of genes and correlation coefficients in this network vary greatly among different cancers, suggesting significantly different strength of gene expression correlation within and between cancer types. For example, in several cancers such as bladder, cervical and uterine cancers, this sub-network contains many fewer components and significant correlations than cancers such as acute myeloid leukemia (AML), lung adenocarcinoma or lower grade brain cancer (Supplementary Fig. 2). We conclude that overexpression of CEN/KT genes can be coordinated by FoxM1 and other factors, but regulatory relationships differ significantly among cancer types and even among individuals within the same type.

Supplementary Note 2. A subset of CEN/KT genes have significant prognostic value in multiple cancers.

We determined if individual CEN/KT gene misregulation has prognostic value for cancer patients by performing meta-analyses on expression microarray datasets for multiple cancer types. These analyses were first performed on >3,000 human breast cancer clinical samples using BC-GenExMiner 3.0[2], then using K-M Plotter database for breast, lung, gastric and ovarian cancers[3]. For breast cancers using BC-GenExMiner, overexpression of 22 individual CEN/KT genes and reduced expression of *CENP-C* are significantly associated with poor any event (AE)-free survival (p<0.05) and poor metastatic relapse-free survival (MRFS) (p<0.05) (Supplementary Table 3). Eleven of these 22 identified genes (*CENP-A, -C, -N, -H, -I, -M, -K, -L, HJURP, MIS18A* and *MIS18B*) are required for new CENP-A assembly, implying an important role in breast cancer progression. Notably, misexpression of nine essential CEN/KT genes (*CENP-T, -S, -P, -Q, -R, M18BP1, PMF1, MIS12* and *NSL1*), including *M18BP1* which is known to be essential for CENP-A assembly, demonstrated lack of overall prognostic value in the meta-analysis using BC-GenExMiner. Analysis using K-M Plotter database identified many of the same genes (Supplementary Table 4)[3]. We conclude that the prognostic value of individual CEN/KT gene misexpression can vary, even when their functions are intimately related, suggesting distinct roles and regulations in cancer progression.

Moreover, we analyzed prognostic values of CEN/KT gene expression for overall survival and disease progression in over 1,600 lung cancer patients, over 350 gastric cancer patients, and a smaller number (n<150) of stage I and stage II ovarian cancer patients, using K-M Plotter[3]. We identified 20 CEN/KT genes whose misexpression impacts lung cancer prognosis when up- or down-regulated (p<0.05) (Supplementary Table 5), 20 for early stage ovarian cancers (p<0.05) (Supplementary Table 6), and 23 for gastric cancer prognosis (p<0.05) (Supplementary Table 7). Most CEN/KT genes significantly associated with prognosis of different types of cancers overlap (Supplementary Table 8). These results suggest that expression levels of many CENK/T genes are effective predictors of breast, lung, gastric, and early stage ovarian cancer prognosis.

Supplementary Note 3. CES signature in breast cancer ILCs.

We examined breast invasive lobular carcinoma (ILC) using the TCGA breast adenocarcinoma dataset. Briefly, we found that ILCs have significantly lower CES than IDCs, and detected significant correlation between CES and both fraction of CNA and mutation frequency within the ILC subcohort (Supplementary Fig. 6A and Supplementary Table 10). ILCs are predominantly luminal A subtype[4], which has the lowest average CES among all molecular subtypes (Supplementary Fig. 5). Because most ILCs belong to luminal A subtype (65/77), we also compared ILCs and IDCs within luminal A subtype. There is no significant difference in CES values between IDCs and ILCs within luminal A subtype (Supplementary Fig. 6B). Within ILCs, we did not detect significant association between high CES and any particular ILC subtype (Supplementary Fig. 6C). We also detected significant correlations between CES and mutation frequency and fraction of CNA for both ILCs and IDCs within luminal A subtype (Supplementary Table 10).

Supplementary Note 4. Prognostic performance of the CES for TCGA datasets.

We evaluated the prognostic value of the CES signature using TCGA breast adenocarcinoma and lung cancer datasets (Supplementary Tables 12 and 13). For breast cancer, we observed significant difference in overall survival across CES tertiles, but the low CES group appears to have worse survival than the intermediate CES group, and has similar survival to the high CES group (Supplementary Fig. 23A). We note that the TCGA dataset at this time suffers from very short follow-up times (median follow-up is 1.8 years for overall survival, Supplementary Table 12). This problem significantly affects survival analyses since most breast cancer patients are expected to live longer than 5-10 years after initial diagnosis under the current standards of care. Indeed, even though we detected highly significant differences in overall survival across PAM50 molecular subtypes (Supplementary Fig. 23B), Kaplan-Meier graph and Cox regression analysis on PAM50 subtypes did not show a significant difference even between basal-like and luminal A subtypes (Supplementary Fig. 23B and Supplementary Table 28), indicating that there are short follow-up or other problems with the dataset, even when it is tested against a well-established marker.

For TCGA lung ADC dataset, CES is a significant prognostic factor in both Kaplan-Meier survival analysis and multivariate Cox regression (Supplementary Fig. 23C and Supplementary table 29), even though the dataset also has short follow-up times (Supplementary Table 13). This is probably because lung ADC patients have significantly shorter median survival after initial diagnosis. Because high CES is also a predictive marker for better response to adjuvant chemotherapy for lung cancer patients (Figure 7), we removed all samples treated with chemotherapy before we analyzed the prognostic value of the CES signature.

For TCGA lung SCC dataset, the CES signature does not significantly prognosticate overall survival (Supplementary Fig. 23D), similar to the result from meta-data analysis (Figure 5C and Supplementary Fig. 16C). However, the CES signature is not only a prognostic marker but also a predictive marker for lung cancer patient outcome after adjuvant chemotherapy or radiotherapy. As we pointed out earlier, it is likely that adjuvant chemotherapy improved survival for high CES patients in the dataset. Unfortunately, the TCGA lung SCC dataset at this time does not provide chemotherapy information (Supplementary Table 13), so we cannot address this issue.

In summary, the CES signature shows significant prognostic value for TCGA breast cancer and lung ADC datasets, but not for lung SCC dataset. However, more careful analyses raised concerns about short follow-up times or lack of treatment information for breast cancer and lung SCC datasets.

Supplementary Note 5. CES predicts NSCLC patient outcome after adjuvant chemotherapy.

Kaplan-Meier analysis of the UT SPORE NSCLC dataset (GSE41274)[5] revealed a lack of significance for the effect of ACT (cisplatin and mainly taxanes) on high CES patients (HR=0.233, log-rank *p*=0.110), likely due to small sample size and short follow-up time for the ACT arm (Supplementary Table 13). Analysis combining the UT SPORE and JBR.10 clinical trials revealed that adjuvant therapy specifically and significantly improved poor OS associated with high CES in early stage NSCLC (HR=0.432, *p*=0.016 for high CES group and HR=1.075, *p*=0.783 for low CES group) (Supplementary Fig. 26). The result confirmed the effectiveness of the CES in predicting NSCLC patient response to adjuvant chemotherapy, including cisplatin.

Supplementary References

1. Thiru, P.*, et al.* Kinetochore genes are coordinately up-regulated in human tumors as part of a FoxM1-related cell division program. *Mol Biol Cell* **25**, 1983-1994 (2014).
2. Jezequel, P.*, et al.* bc-GenExMiner 3.0: new mining module computes breast cancer gene expression correlation analyses. *Database (Oxford)* **2013**, bas060 (2013).
3. Gyorffy, B.*, et al.* An online survival analysis tool to rapidly assess the effect of 22,277 genes on breast cancer prognosis using microarray data of 1,809 patients. *Breast Cancer Res Treat* **123**, 725-731 (2010).
4. Ciriello, G.*, et al.* Comprehensive Molecular Portraits of Invasive Lobular Breast Cancer. *Cell* **163**, 506-519 (2015).
5. Tang, H.*, et al.* A 12-gene set predicts survival benefits from adjuvant chemotherapy in non-small cell lung cancer patients. in *Clin Cancer Res*, Vol. 19 1577-1586 (2013).
